# A pictorial key for identification of the hoverflies (Diptera: Syrphidae) of the Madeira Archipelago

**DOI:** 10.3897/BDJ.10.e78518

**Published:** 2022-03-21

**Authors:** Carla Rego, John Smit, António Franquinho Aguiar, Délia Cravo, Andreia Penado, Mário Boieiro

**Affiliations:** 1 Centre for Ecology, Evolution and Environmental Changes, Azorean Biodiversity Group, Faculty of Sciences, University of Lisbon, Lisboa, Portugal Centre for Ecology, Evolution and Environmental Changes, Azorean Biodiversity Group, Faculty of Sciences, University of Lisbon Lisboa Portugal; 2 European Invertebrate Survey - the Netherlands / Naturalis Biodiversity Center, Leiden, Netherlands European Invertebrate Survey - the Netherlands / Naturalis Biodiversity Center Leiden Netherlands; 3 Laboratório de Qualidade Agrícola, Secretaria Regional de Agricultura e Desenvolvimento Rural, Camacha, Madeira, Portugal Laboratório de Qualidade Agrícola, Secretaria Regional de Agricultura e Desenvolvimento Rural Camacha, Madeira Portugal; 4 Centre for Ecology, Evolution and Environmental Changes, Azorean Biodiversity Group, University of Azores, Angra do Heroísmo, Portugal Centre for Ecology, Evolution and Environmental Changes, Azorean Biodiversity Group, University of Azores Angra do Heroísmo Portugal

**Keywords:** Flower flies, Macaronesia, Madeira endemics, photographic guide, species identification, syrphids, taxonomic key

## Abstract

**Background:**

Syrphid flies are important ecological indicators and provide crucial ecosystem services, being important pollinators and biological control agents of insect pests. These charismatic insects are conspicuous and, due to their size and colourful patterns, are relatively easy to identify. However, the lack of user-friendly literature (e.g. photographic guides) for most areas may hamper its wider selection as a study group in biodiversity and ecological studies. The syrphid fauna of Madeira Archipelago comprises 26 species, including four endemics (*Eumerushispidus* Smit, Aguiar & Wakeham-Dawson, 2004; *Melanostomawollastoni* Wakeham-Dawson, Aguiar, Smit, McCullough & Wyatt, 2004; *Myathropausta*, Wollaston, 1858 and *Xanthandrusbabyssa*, Walker, 1849), but, despite the current good taxonomic knowledge on this group, information on species distribution, ecology and conservation is still lacking. Here, we provide a pictorial key to the adult hoverflies of Madeira Archipelago highlighting diagnostic characteristics and present photographs of both males and females (in dorsal and lateral views) in colour plates. The key and plates will help researchers to differentiate these species, thus encouraging the use of this insect group in future bioindication studies. In addition, this study also aims to engage a broader audience of non-experts in improving the knowledge on the distribution and ecology of Madeira syrphids.

**New information:**

We provide a checklist for the hoverflies of Madeira Archipelago and a pictorial key to help on species identification.

## Introduction

Syrphids, commonly known as hoverflies or flower flies, belong to a large family of flies (Diptera: Syrphidae) with over 6,000 known species ([Bibr B7483146][Bibr B7639703]). These flies are conspicuous and easy to distinguish from other insects due to their colour patterns, morphology and flying behaviour (Ball and Morris 2015). Adult hoverflies feed on honeydew, pollen and nectar and are amongst the most important flower visiting insects in many ecosystems (Wackers et al. 2008, Inouye et al. 2015, Doyle et al. 2020). Larvae, on the other hand, exploit a wide variety of food resources, including fungal fruiting bodies (mycophagous), dung, tree sap, nests of social insects, decaying vegetation and wood (saprophagous), whereas other larvae mine the leaves and stems of plants (phytophagous) or predate other insects (zoophagous), including leafhoppers, coccids and aphids (Rotheray 1993). For these reasons, hoverflies are considered important pollinators and biological control agents of insect pests, providing crucial ecosystem services (Ankersmit et al. 1986, Nelson et al. 2012, Dunn et al. 2020, Pekas et al. 2020). More recently, the role of hoverflies as ecological indicators has also been stressed in many studies since they are easy to sample and identify, their life cycle is well-known and the larvae from different species have distinct environmental requirements for their development (Sommaggio 1999, Sommaggio and Burgio 2014, Ball and Morris 2015, Dunn et al. 2020).

The syrphid fauna of Madeira has been studied since the mid-nineteenth century by several authors who contributed to a better understanding of species diversity and distribution in this Archipelago (Walker 1849, Wollaston 1858, Loew 1860, Schiner 1868, Thomson 1869, Bigot 1884, Osten-Sacken 1884, Becker 1908, Becker 1921, Frey 1939, Frey 1949). Frey (1949) updated the syrphid fauna checklist and reported 21 species for Madeira Archipelago. During this first century of reports on the Madeiran syrphid fauna, the number of recorded species increased considerably, showing a similar pattern to most of the other groups of terrestrial arthropods in the Archipelago (Borges et al. 2008). More recently, other authors made significant contributions to the knowledge of this group of flies in Madeira Archipelago (Gomes and Baez 1990, Barkemeyer 1999, Pita and Gomes 2003, Smit et al. 2004, Wakeham-Dawson et al. 2004, Aguiar et al. 2005, Pita et al. 2009). For instance, both Smit et al. (2004) and Wakeham-Dawson et al. 2004 described new endemic species, clarified the identity of ambiguous taxa and provided a thorough revision of Madeiran Syrphidae nomenclature. The current knowledge on the taxonomic diversity of Madeiran syrphids was updated by Smit (2008) and it was included in a comprehensive reference work on Madeira Archipelago biodiversity (Borges et al. 2008). Smit (2008) listed 26 species of hoverflies including four endemics (*Eumerushispidus* Smit, Aguiar & Wakeham-Dawson, 2004; *Melanostomawollastoni* Wakeham-Dawson, Aguiar, Smit, McCullough & Wyatt, 2004; *Myathropausta*, Wollaston, 1858 and *Xanthandrusbabyssa*, Walker, 1849) and provided general information on species distribution in the Archipelago. Despite the current good taxonomic knowledge on Madeiran syrphids, there is still a significant gap regarding species distribution, ecology and conservation.

During the last decades, there has been a growing interest in biodiversity conservation by the general public that has extended to several charismatic invertebrate groups, such as dragonflies and butterflies. In oceanic islands, like Madeira, invertebrate conservation needs to be fostered by engaging researchers, decision-makers and common citizens in knowing, valuing, protecting and making public the unique diversity of life forms of these ecosystems. This interplay is urgent since the biodiversity of oceanic islands worldwide is under threat due to various factors (e.g. land-use change, invasive species, climate change) and, jointly with significant declines in endemic species abundance, many human-driven extinctions have been documented in these unique ecosystems, including in Madeira Archipelago (Goodfriend et al. 1994, Gardiner 2003, Fontaine et al. 2007, Régnier et al. 2009, Rando et al. 2012, Terzopoulou et al. 2015). Halting biodiversity loss in oceanic islands is mandatory and, to accomplish this goal, a multidisciplinary strategy needs to be implemented. This strategy should include monitoring programmes targeting specific invertebrate groups and the use of expeditious, user-friendly and reliable techniques (Borges et al. 2018).

Here, we aim to provide a user-friendly pictorial key for the identification of Madeira’s hoverflies, a charismatic bioindicator and ecologically-important insect group. The key was designed for use by non-experts and, altogether with the photos of male and female specimens of all known species occurring in the Archipelago, aims to engage a diverse audience in improving current knowledge on these conspicuous flies.

## Materials and methods

### Study area

Madeira Archipelago is located in the Atlantic Ocean, nearly 600 km from the African coast (Morocco) and 450 km north from the Canary Islands, between latitudes 32°24′ and 33°07′N and longitudes 16°16′ and 17°16′W. The Archipelago is formed by three groups of volcanic islands and islets: Madeira, Porto Santo and the Desertas Islands (Boieiro et al. 2015). Madeira is the largest island of the group (~ 740 km^2^) and is characterised by a rugged topography with a steep coastline combined with deep ravines, high peaks and an altitudinal plateau in the central part of the Island (the highest mountaintop is Pico Ruivo at 1862 m). Madeira harbours various habitats, from coastal dry areas to humid laurel forests (Laurisilva) and heathland at higher altitudes, that support a diverse fauna and flora (Menezes et al. 2011, Boieiro et al. 2013, Boieiro et al. 2015). Porto Santo, the second largest island, lies ~ 40 km NE of Madeira, has several islets surrounding it, all included in a network of protected areas (Alves et al. 2015). Natural vegetation cover was severely destroyed by human activities following colonisation and, currently, is mostly composed of herbaceous plants which are scarce in some areas. Further, in some mountaintops, pine forests were planted to mitigate soil erosion effects. The Desertas Islands is a group of three islands (Ilhéu Chão, Deserta Grande and Bugio) that include a Nature Reserve. Both Bugio and Deserta Grande (the largest ones) are crest-like islands with steep slopes and have large areas deprived of vegetation or are covered by herbaceous plants while Ilhéu Chão, with a flat surface, has a well-preserved herbaceous vegetation cover (Menezes et al. 2005).

### Laboratory work

Specimens, both males and females, from all the known species reported to Madeira Archipelago were studied under a stereomicroscope. Most of the specimen’s images were taken with a Leica M125 motorised stereomicroscope, equipped with a IC80 HD digital camera and LAS-Leica Application Suite 3.8 Software. For image stacking, we used the LAS Module “Multifocus” and post-processed the images in ®Adobe Photoshop CC. We also used a Canon 7D digital slr camera with a Canon EF 100 mm 2.8 L Is USM macro lens to capture the habitus of some specimens. The study specimens are deposited in the entomological collections of the Laboratório de Qualidade Agrícola (ICLAM) (Madeira, Portugal) and Naturalis Biodiversity Center, Natural (RMNH) (Leiden, the Netherlands).

## Identification Keys

### Key to the hoverflies of the Madeira Archipelago

**Table:** 

1	Face entirely yellow (i); scutellum always yellow, clearly lighter than scutum (ii) (Fig. 1)	2
–	Face dark or yellow with median dark stripe, sometimes obscured by dense pollinosity (*); scutellum never yellow, sometimes orange-brown (**) (Fig. 1)	15
2	Very large species, over 18 mm, with hornet-like appearance; scutum with yellow markings on anterior half and orange-red colouration on posterior half; metafemur with a small tooth apicoventrally (i); wings with a yellow tinge along the costa (ii) (Fig. 2)	***Milesiacrabroniformis***
–	Smaller species, at most 15 mm, never with a hornet-like appearance; if yellow markings present on scutum, then restricted to lateral margins and scutum never with orange-red markings; metafemur never with a small tooth apicoventrally; wings without yellow tinge along costa	3
3	Thoracic pleura with distinct yellow markings (i); scutum with distinct yellow bands laterally (ii) (Fig. 3)	4
–	Thoracic pleura without yellow markings (*); scutum without distinct yellow bands laterally (**) (Fig. 3)	6
4	Abdomen distinctly margined (i); male with tooth-like protuberance on metatrochanter (ii) (Fig. 4)	***Ischiodonaegyptius***
–	Abdomen not margined (*); male without tooth-like protuberance on metatrochanter (Fig. 4)	5
5	Scutum with yellow lateral band restricted to the anterior part of wing base (i); abdomen of males about as long as wings when folded; smaller species: 5-8 mm (Fig. 5)	***Sphaerophoriarueppelli***
–	Scutum with yellow lateral band uninterrupted, continuing posteriorly of wing base (*); abdomen of males clearly longer than wings when folded (**); larger species: 7-12 mm (Fig. 5)	***Sphaerophoriascripta***
6	Abdomen more or less parallel-sided, as broad as scutum (i) (Fig. 6)	7
–	Abdomen clearly broadening and oval shaped, clearly broader than scutum (*) (Fig. 6)	8
7	Abdomen with ‘double bands’ on t3, t4 (i) (Fig. 7)	***Episyrphusbalteatus***
–	Abdomen without ‘double bands’, with oblique yellow spots (*), sometimes connected to form bands (Fig. 7)	***Meliscaevaauricollis***
8	Wing vein R4+5 in basal half of cell r4+5 almost parallel to M, curving upwards in apical part (i); eyes pilose (ii); frons distinctly swollen, more obvious in males (iii); larger species: 10-15 mm (Fig. 8)	9
–	Wing vein R4+5 more or less straight, converging from vein M from the base of cell r4+5 (*); eyes bare, except *S.torvus* (**); frons not swollen (***); smaller species: 7-13 mm (Fig. 8)	11
9	T3 and t4 with slender yellow or white lunulate maculae, clearly constricted in the middle (i), yellow or white markings covering less than half the length of t3 (Fig. 9)	10
–	T3 and t4 with larger yellow maculae, which are at most slightly constricted in the middle (+), yellow markings covering more than half the length of t3 (Fig. 9)	***Scaevaalbomaculata***
10	Abdominal spots yellow in live specimens; spots on t3 with hind edges curved, their outer and inner corners equally close to anterior edge of the tergite (*) (Fig. 9)	***Scaevaselenitica***
–	Abdominal spots almost white in live specimens; spots on t3 with hind edges straight and oblique, their outer corners distinctly further removed from anterior edge of tergite than inner corner (ii) (Fig. 9)	***Scaevapyrastri***
11	Scutum pollinose and dull (i); abdomen with relatively slender yellow bands on t3-t4 (ii); ventral calypter with long erect pili on dorsal surface (iii) (Fig. 10)	12
–	Scutum pollinose, but clearly shining (*); abdomen typically with yellow spots on t3-t4, sometimes connected to form bands (**); ventral calypter lacking long erect pili on dorsal surface (***) (Fig. 10)	13
12	Eyes pilose, sparse and short in females (i); wing cell BM entirely covered by microtrichia (ii) (Fig. 11)	***Syrphustorvus***
–	Eyes bare (*); wing cell BM basal ¼ bare (**) (Fig. 11)	***Syrphusvitripennis***
13	Face in frontal view at least as broad as one eye (i); femora at least partially black at the base (ii) (Fig. 12)	14
–	Face in frontal view clearly narrower than one eye (*); femora entirely yellow (**); abdomen normally with broad yellow maculae, sometimes connected to form bands (Fig. 12)	***Eupeodesnuba***
14	Scutellum predominantly yellow pilose (i); abdominal maculae reaching lateral margins of tergites, normally with spots, but frequently connected to form bands (ii); male with larger genitalia (iii) (Fig. 13)	***Eupeodescorollae***
–	Scutellum predominantly black pilose (*); abdominal maculae not reaching lateral margins of tergites, usually with spots, only rarely connected to form bands (**); male with smaller genitalia (***) (Fig. 13)	***Eupeodesluniger***
15	Wing vein R4+5 with a strong dip in the cell below (i); larger species (10-16 mm), sometimes with metallic bronze luster, but often with a bee-like appearance (Fig. 14)	16
–	Wing vein R4+5 without a strong dip in the cell below (*); smaller species (4-12 mm), never with a bee-like appearance (Fig. 14)	19
16	Eyes spotted (i). Entire body largely with metallic bronze luster (Fig. 15)	***Eristalinusaeneus***
–	Eyes never spotted, but either striped or concolorous. Body without bronze luster	17
17	Eyes striped (*) (Fig. 15)	***Eristalinustaeniops***
–	Eyes concolorous, without stripes (+) (Fig. 15)	18
18	Eyes with bands of pili (i); wing cell R1 closed (ii) (Fig. 16)	***Eristalistenax***
–	Eyes without bands of pili; wing cell R1 open (**) (Fig. 16)	***Myathropausta***
19	Face in profile with a facial tubercule (i) (Fig. 17)	20
–	Face in profile more or less straight (*), sometimes mouth-edge clearly protruding (**) (Fig. 17)	23
20	Face entirely black (i); abdomen either entirely dark or with orange-yellow spots; larger species: 5-12 mm (Fig. 18)	21
–	Face creamy yellow with a black facial stripe (*); abdomen black or partially red; very small species: 4-6 mm (Fig. 18)	***Paragusmundus***
21	Abdomen oval, clearly broader than scutum (i); female with abdomen entirely black or with very small, rounded spots (i); male with broad orange-yellow spots on t3 and t4 (ii), which are sometimes connected (Fig. 19)	***Xanthandrusbabyssa***
–	Abdomen slender and parallel-sided, as broad as scutum (*) (Fig. 19)	22
22	Abdomen entirely black in males (**), at most with reduced orange markings in the female (*); larger species: 7-10 mm (Fig. 19)	***Melanostomawollastoni***
–	Abdomen with clear orange markings, triangular on t3 and t4 in females (+), rectangular in males (++); smaller species: 5-8 mm (Fig. 19)	***Melanostomamellinum***
23	Abdomen clearly petiolate, t2 constricted in basal half (i); smaller species: 5-6 mm (Fig. 20)	***Neoasciapodagrica***
–	Abdomen never that clearly petiolate, t2 never constricted in basal half, abdomen more or less parallel-sided. Larger species: 6-13 mm (Fig. 20)	24
24	Abdomen with a broad orange band (*); larger species: 10-13 mm (Fig. 20)	***Xylotasegnis***
–	Abdomen without orange; smaller species 6-10 mm (+) (Fig. 20)	25
25	Thoracic pleura not dusted (i) and scutum with a pair of longitudinal pollinose vittae, almost reaching scutellum (ii); legs entirely black (iii); abdomen with pollinose spots on t2-t4 (iiii) (Fig. 21)	*Eumerushispidus*
–	Thoracic pleura heavily dusted, continuing on the frontal half of the lateral side of scutum (*); legs bicoloured (**); abdomen with yellow spots on the lateral sides of t2 and t3, t4 with dusted areas (Fig. 21)	*Syrittapipiens*

The taxonomic key to the adult stages of hoverfly species of the Madeira Archipelago relies on pictorial information to ease interpretation of characters and includes information on morphological differences between males and females. Additionally, photos of male and female specimens of all species (in dorsal and lateral views) are presented in colour plates. In the dichotomous key, the couplet leads present one or more morphological characteristics indicated with symbols (e.g. asterisks for one lead and Roman numerals for the other) which help to easily identify those characteristics in the associated figures. The terminology of morphological characters used in this key follows Thompson (1999). Abdominal tergites and sternites are abbreviated with a ‘t’ or ‘s’, respectively.

## Analysis

### The hoverfly species of Madeira Archipelago

The syrphid fauna of Madeira Archipelago comprises 26 species, all considered to be native to these islands (Table 1). Four of them are endemics (*Eumerushispidus*, *Melanostomawollastoni*, *Myathropausta* and *Xanthandrusbabyssa*) which occur mostly in native laurel forests (Laurisilva) and altitudinal heathlands in Madeira Island (except for *E.hispidus* which occurs in coastal and drier habitats of different islands) (Smit et al. 2004, Smit 2008). There are significant differences on the syrphid fauna between island groups since 25 species were recorded in Madeira, 14 in Porto Santo and seven in Desertas Islands. Differences in island area, altitude and habitat heterogeneity are the main drivers of species richness differences between islands, with some species being restricted to native forest areas in Madeira Island. We believe that the syrphid species inventory of Madeira Archipelago is near complete, but additional sampling should be carried out in Porto Santo and Desertas since these smaller islands were less sampled than Madeira proper. Furthermore, taking in consideration the increase in trade and tourism to Madeira in recent decades, it is expected that new species will arrive at the Archipelago; therefore, it is important to implement a monitoring scheme for early detection of introduced species in the short term.

## Figures and Tables

**Figure 1.**
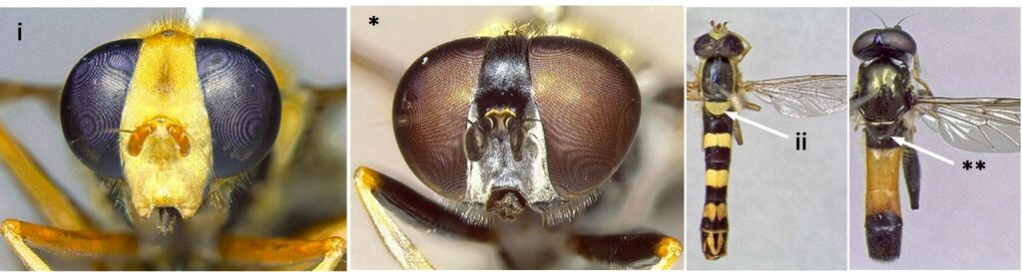
Differences in face and scutellum colouration patterns.

**Figure 2.**
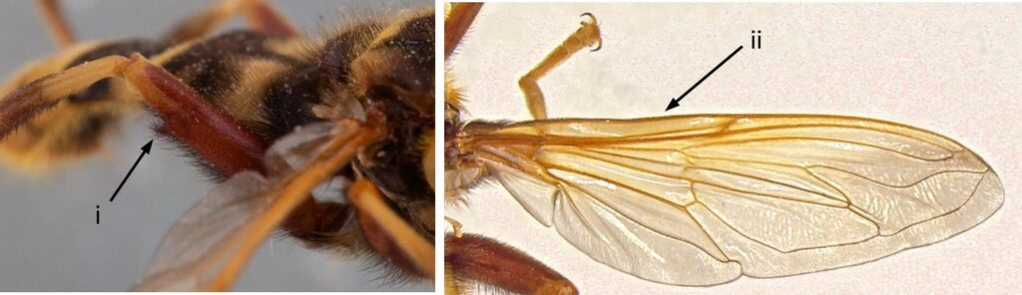
Small apicoventral tooth in metafemur and yellow tinge along the costa.

**Figure 3.**
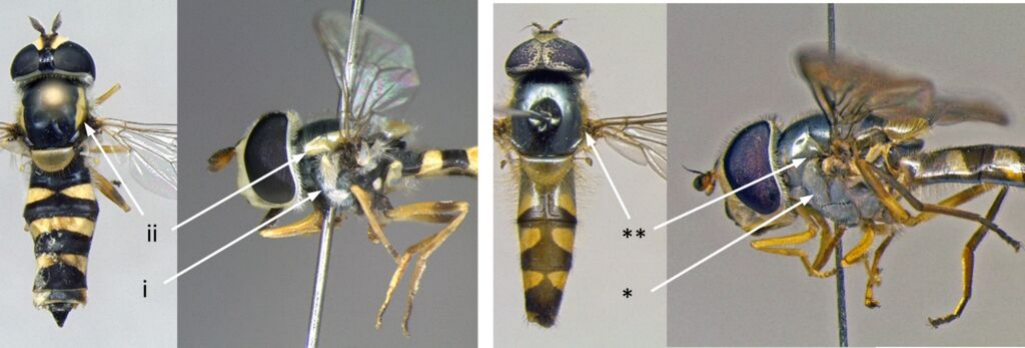
Differences in scutum and thoracic pleura colouration.

**Figure 4.**
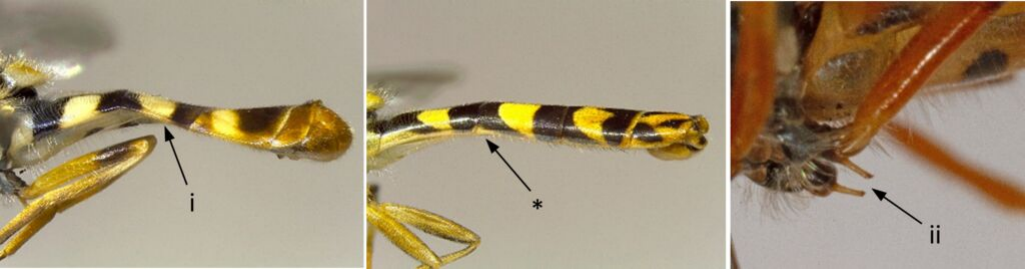
Differences in abdomen margin and metatrochanter protuberance.

**Figure 5.**
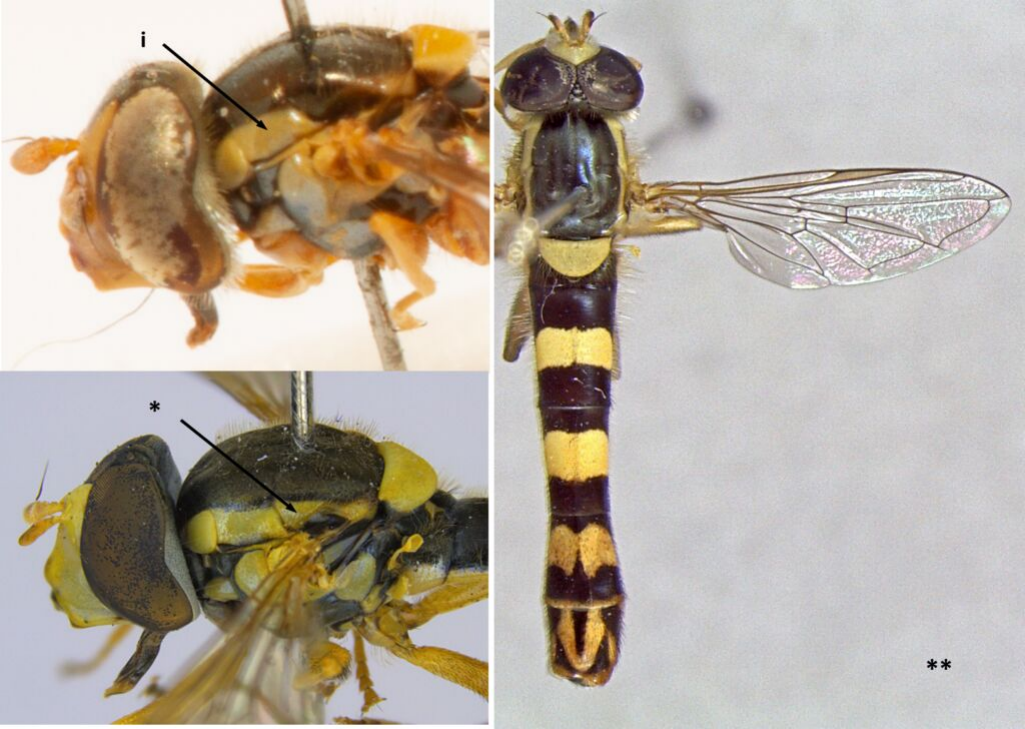
Differences in scutum colouration and abdomen morphology.

**Figure 6.**
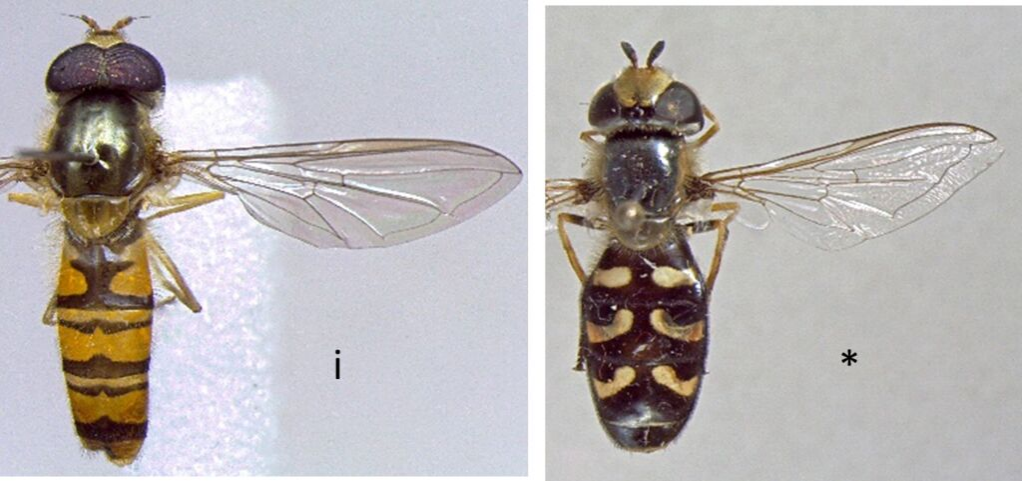
Differences in abdomen morphology.

**Figure 7.**
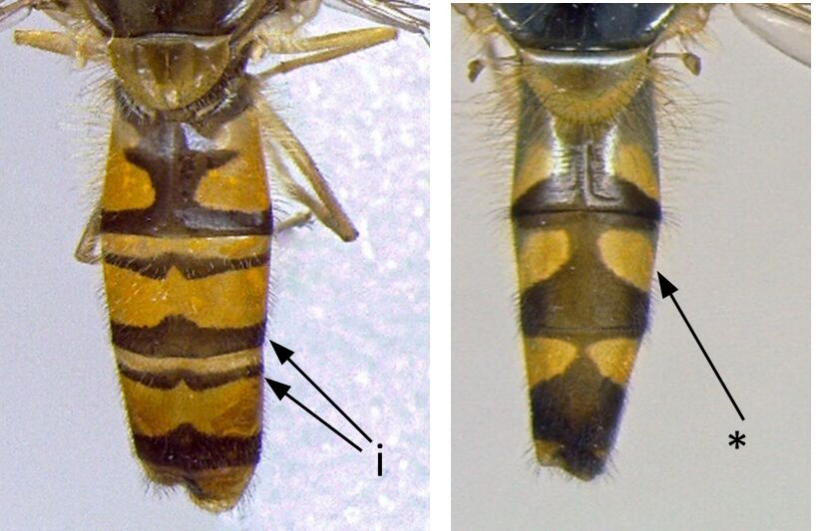
Differences in abdomen colouration patterns.

**Figure 8.**
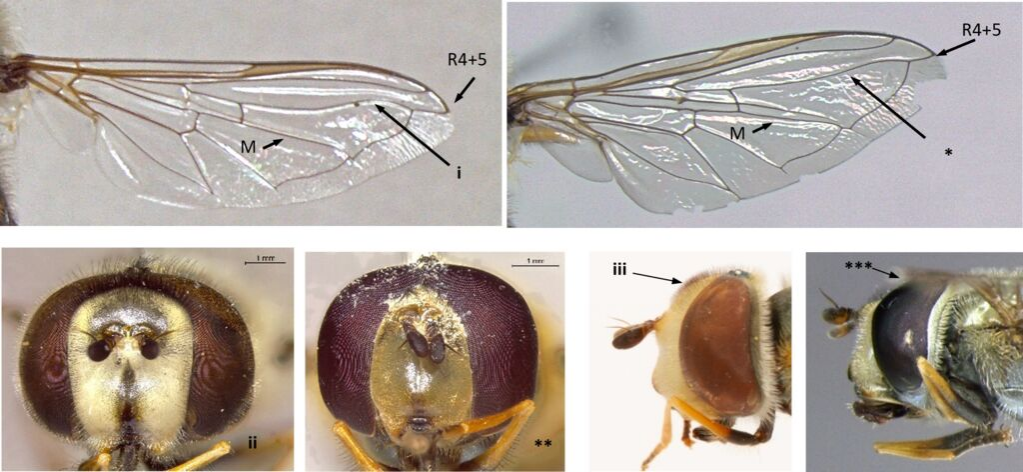
Differences in eye pilosity, frons and vein R4+5 morphology.

**Figure 9.**
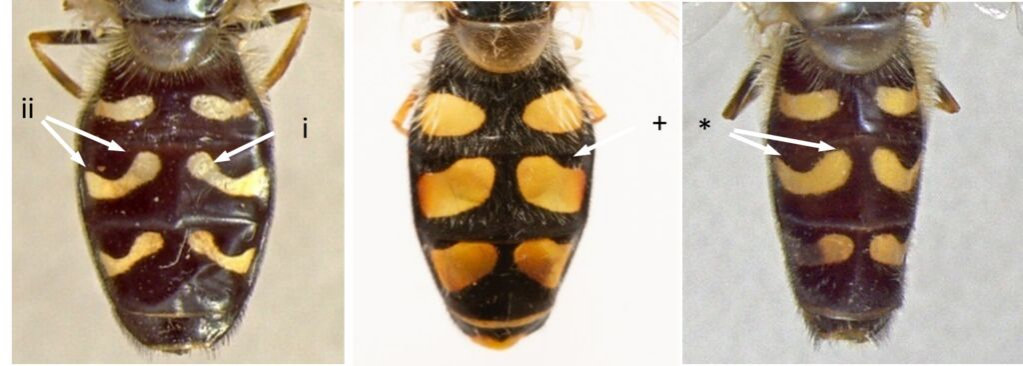
Differences in colouration pattern of t3 and t4.

**Figure 10.**
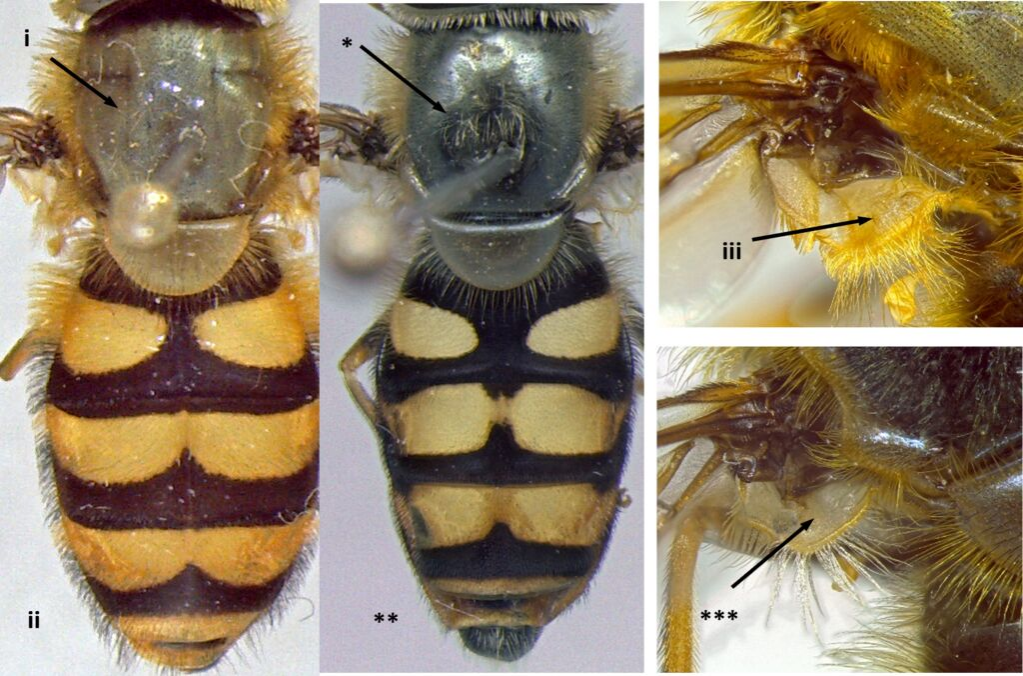
Differences in scutum shine, colouration pattern of t3-t4 and in the presence of long erect pili on dorsal surface of ventral calypter.

**Figure 11.**
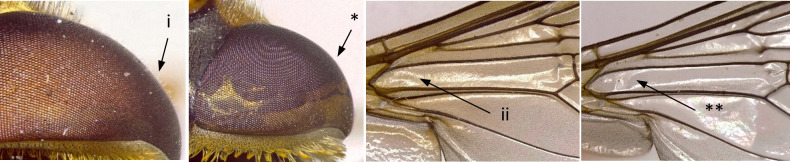
Differences in eye and wing cell BM pilosity.

**Figure 12.**
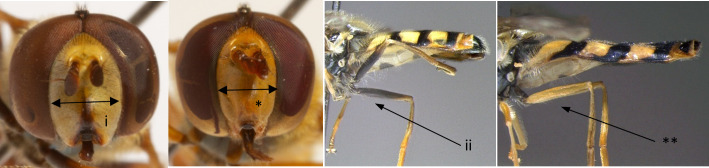
Differences in face morphology and femora colour.

**Figure 13.**
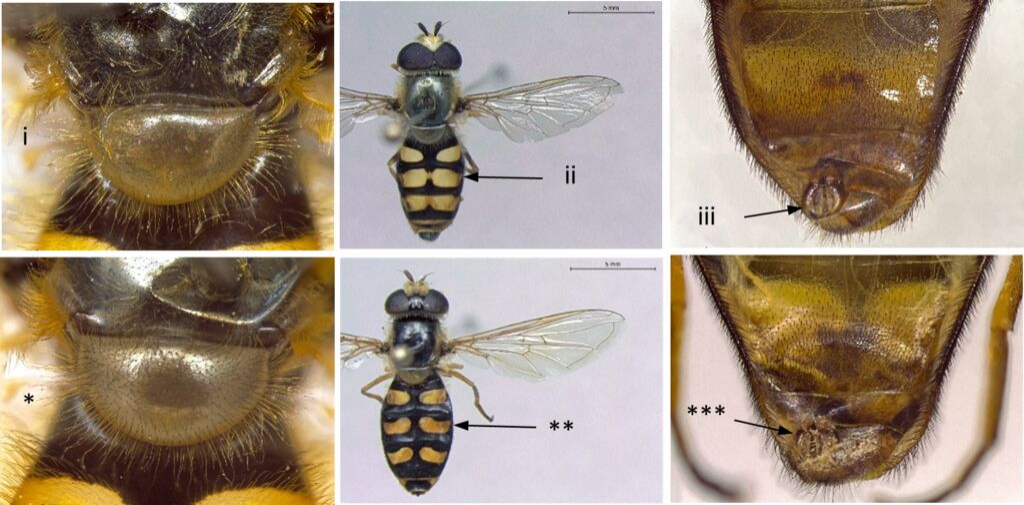
Differences in scutellum pilosity, abdominal colouration pattern and male genitalia.

**Figure 14.**
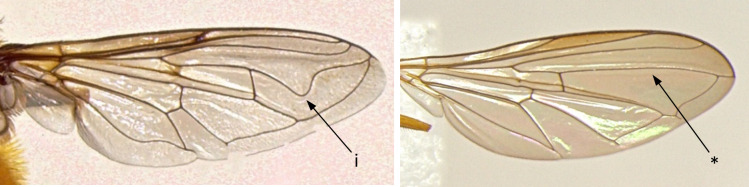
Differences in wing vein R4+5 morphology.

**Figure 15.**
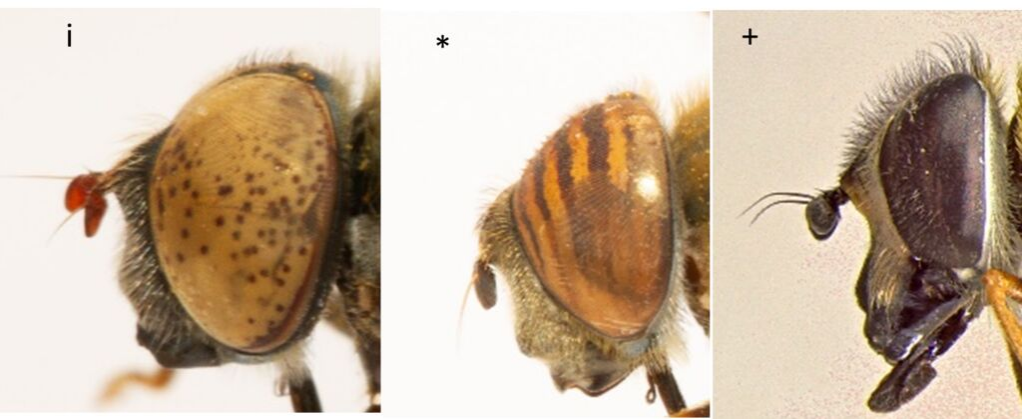
Differences in eye colour patterns.

**Figure 16.**
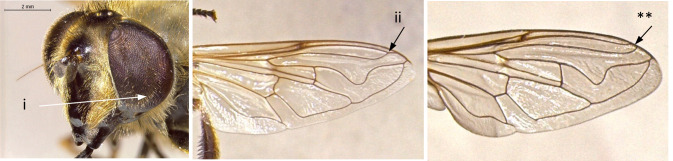
Differences in eye pili bands and wing cell R1 morphology.

**Figure 17.**
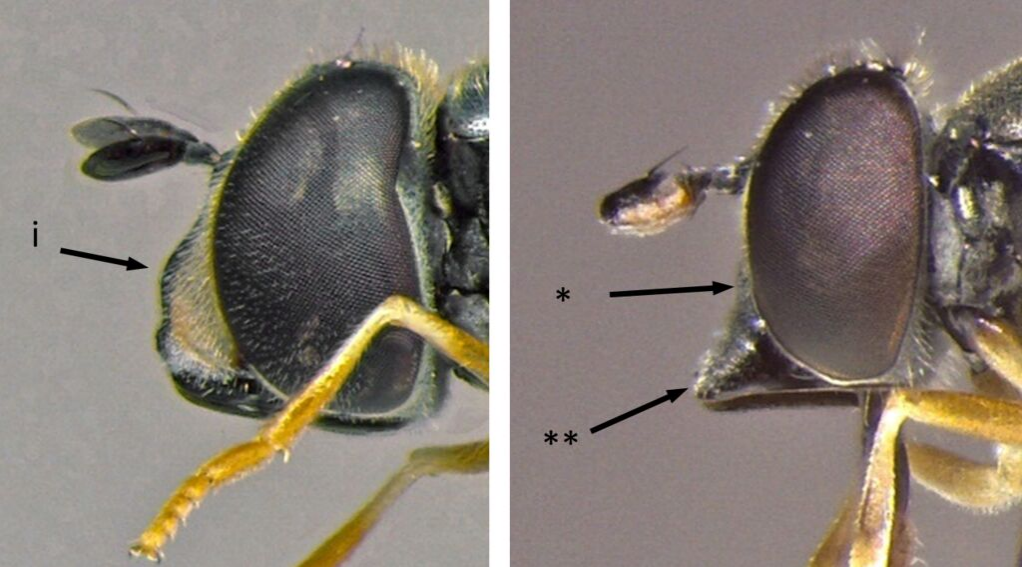
Differences in face profile.

**Figure 18.**
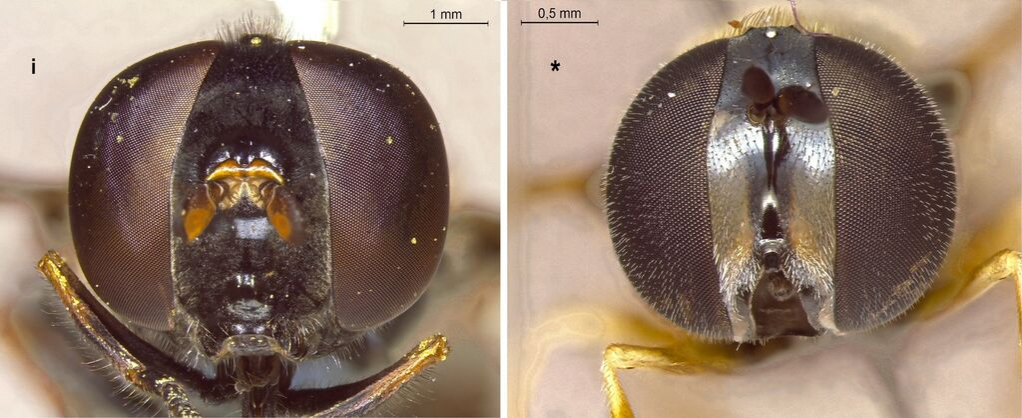
Differences in face colour.

**Figure 19.**
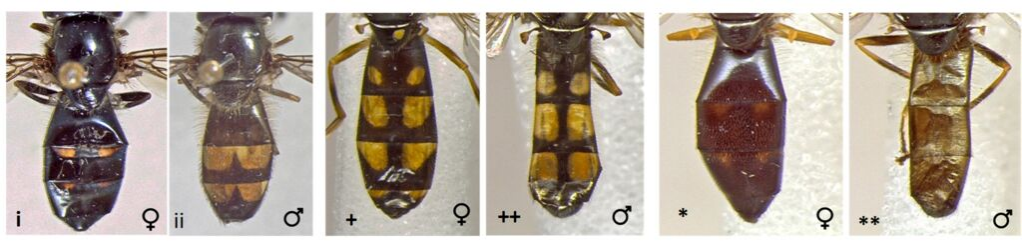
Differences in abdomen morphology and colour patterns.

**Figure 20.**
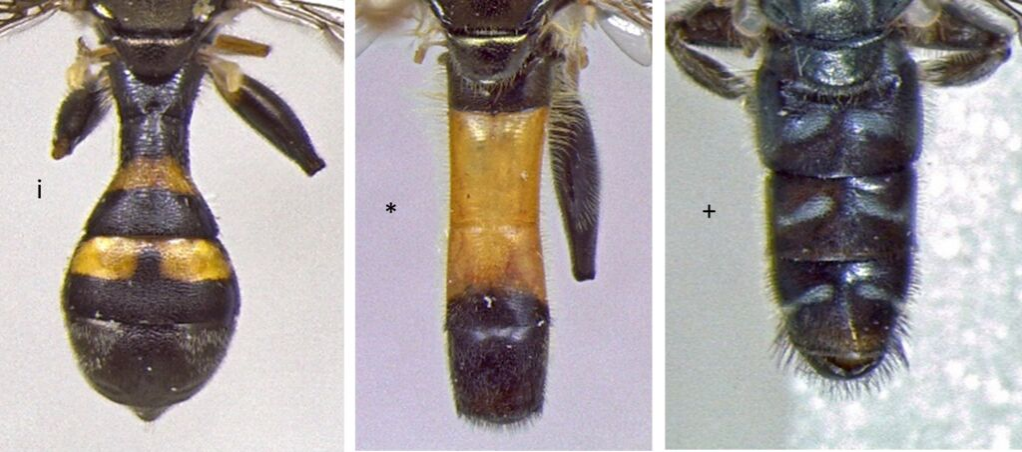
Differences in abdomen morphology and colouration.

**Figure 21.**
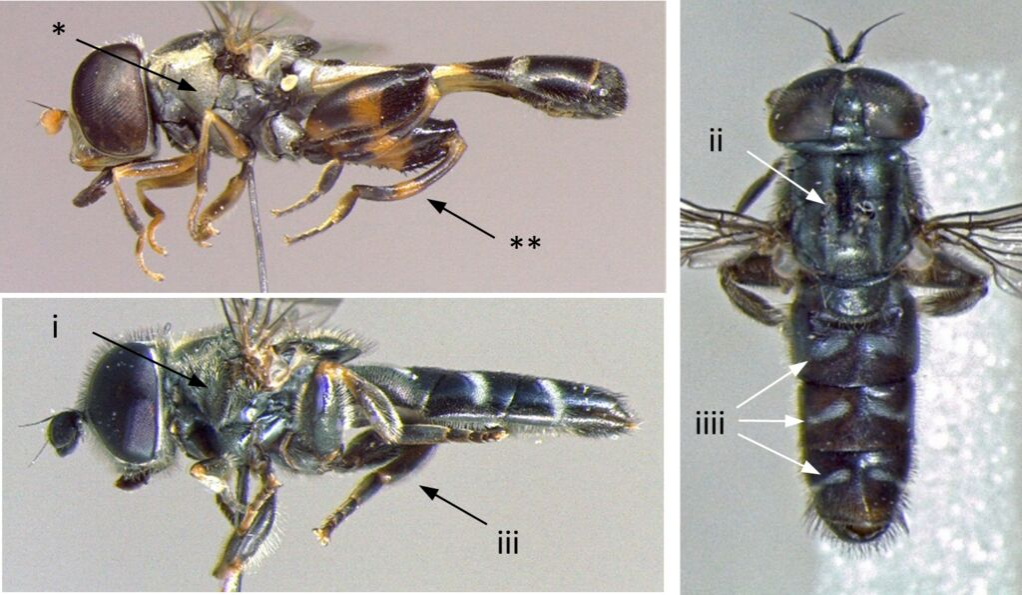
Differences in leg colouration and body pollinosity.

**Figure 22a.**
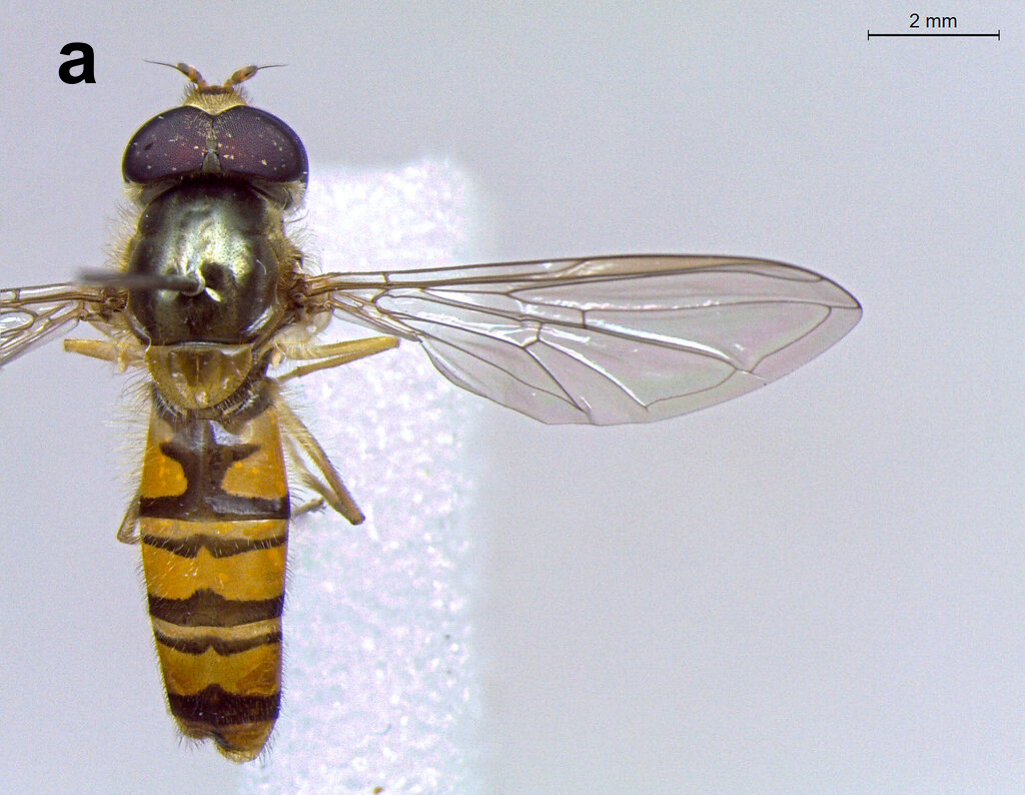
male in dorsal view

**Figure 22b.**
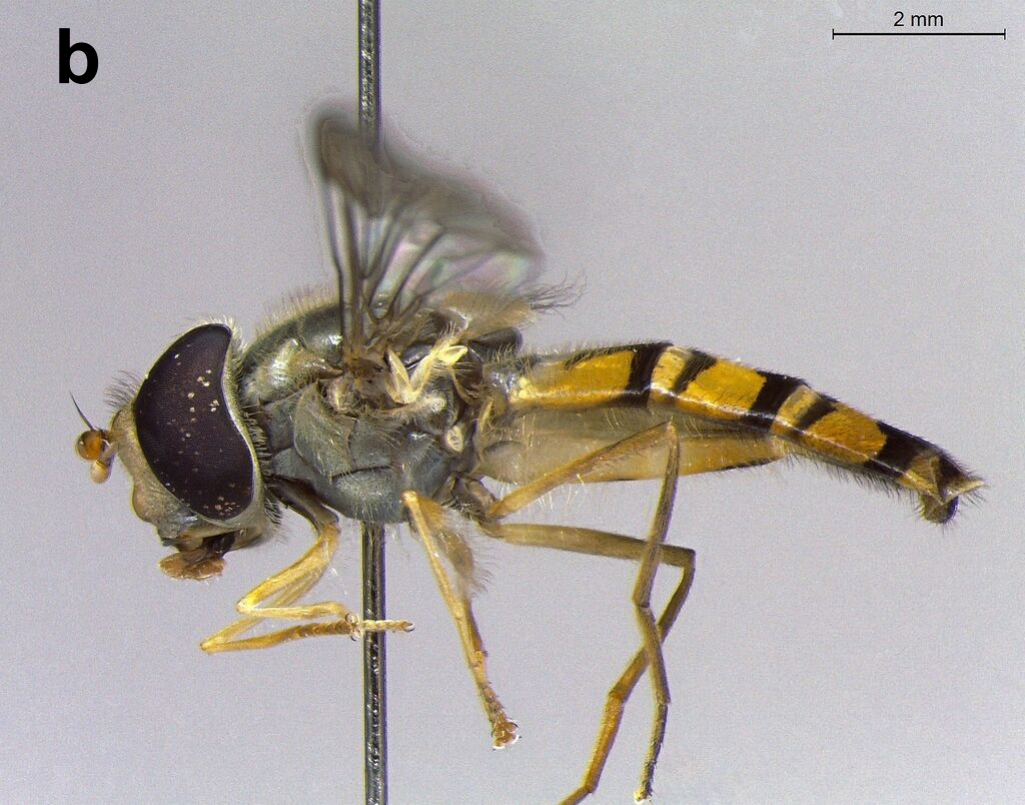
male in profile

**Figure 22c.**
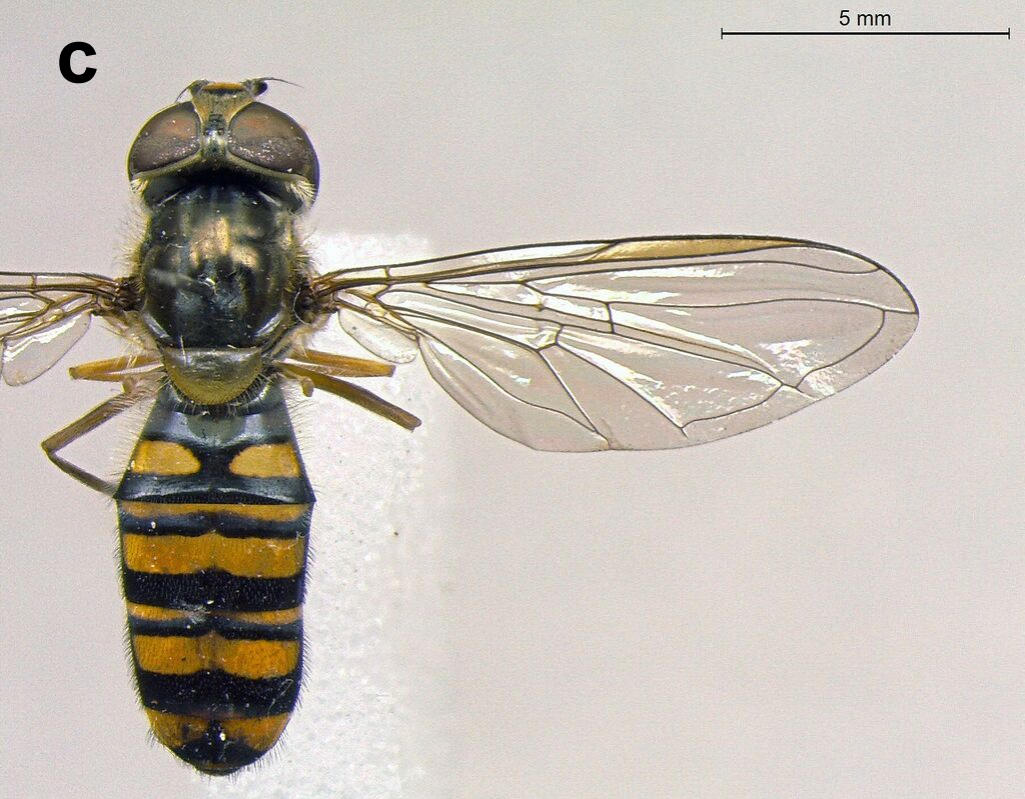
female in dorsal view

**Figure 22d.**
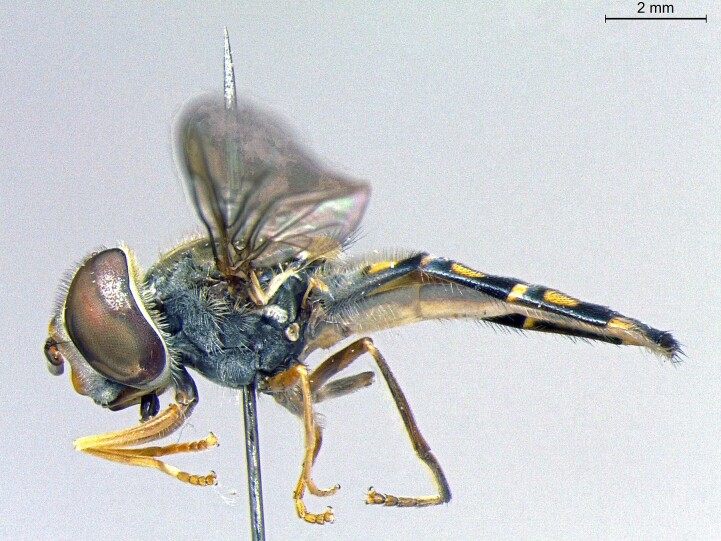
female in profile

**Figure 23a.**
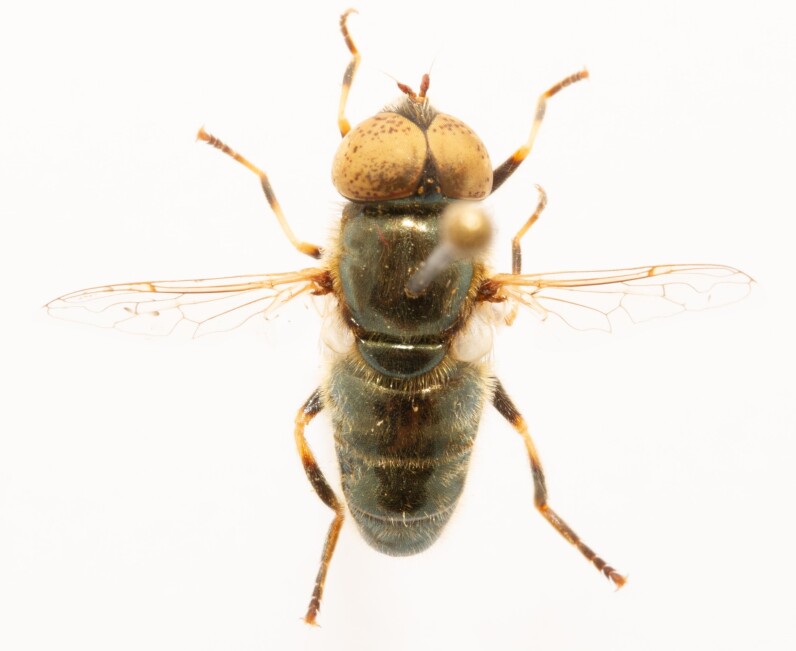
male in dorsal view

**Figure 23b.**
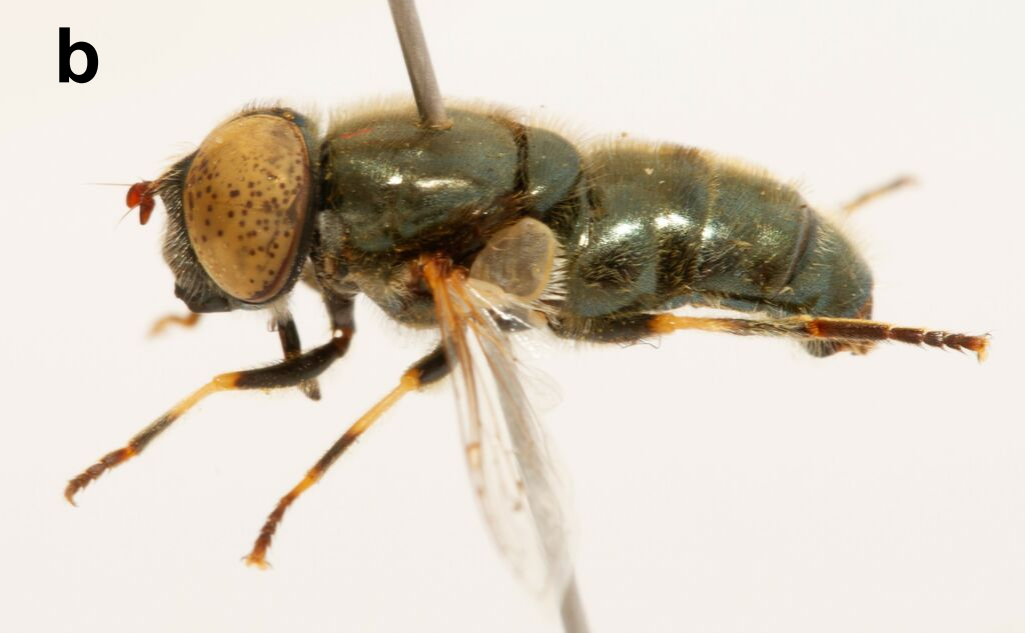
male in profile

**Figure 23c.**
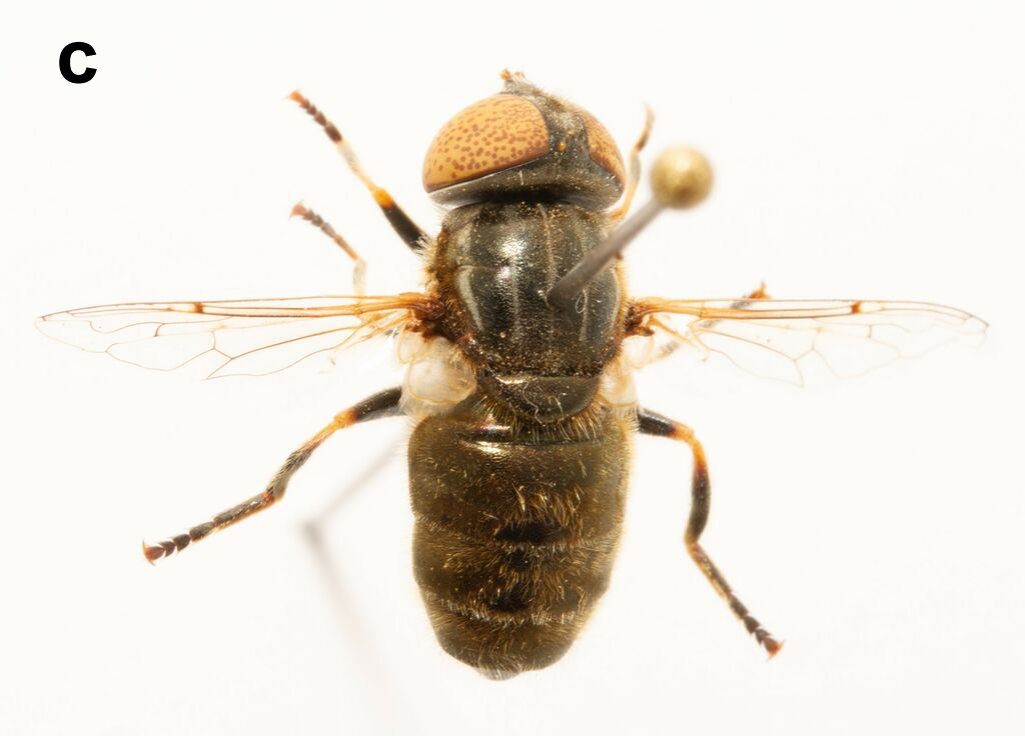
female in dorsal view

**Figure 23d.**
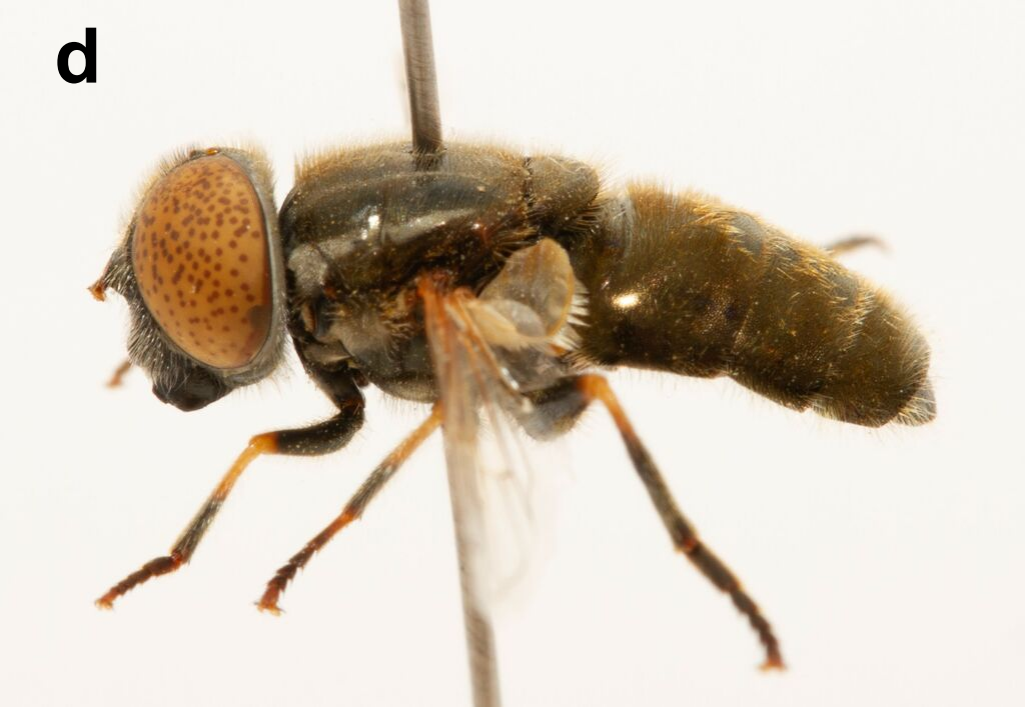
female in profile

**Figure 24a.**
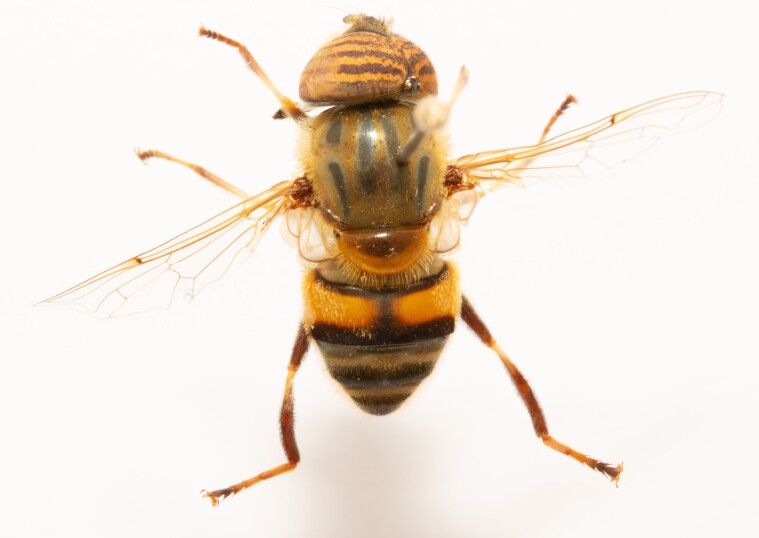
male in dorsal view

**Figure 24b.**
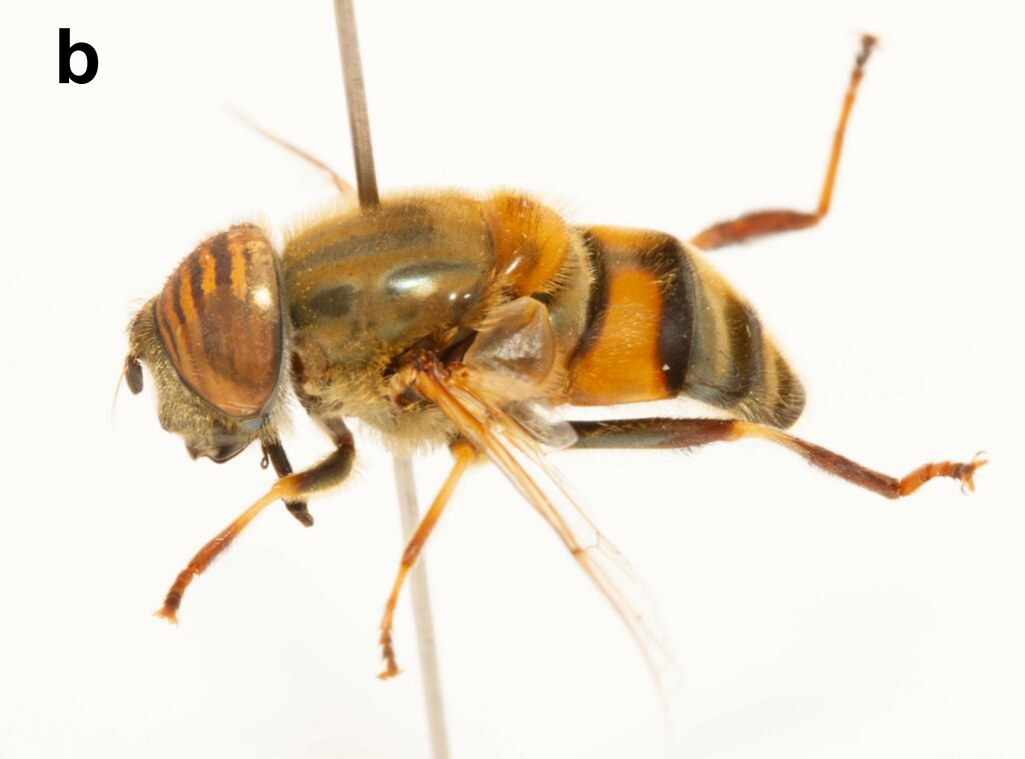
male in profile

**Figure 24c.**
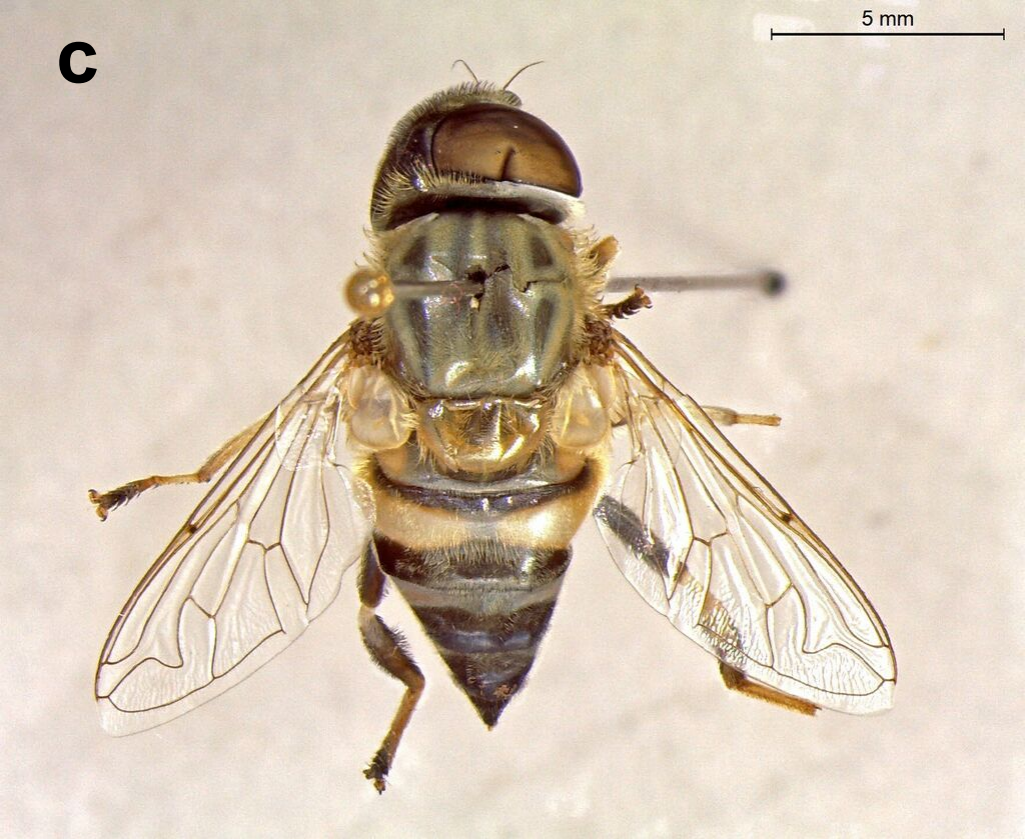
female in dorsal view

**Figure 24d.**
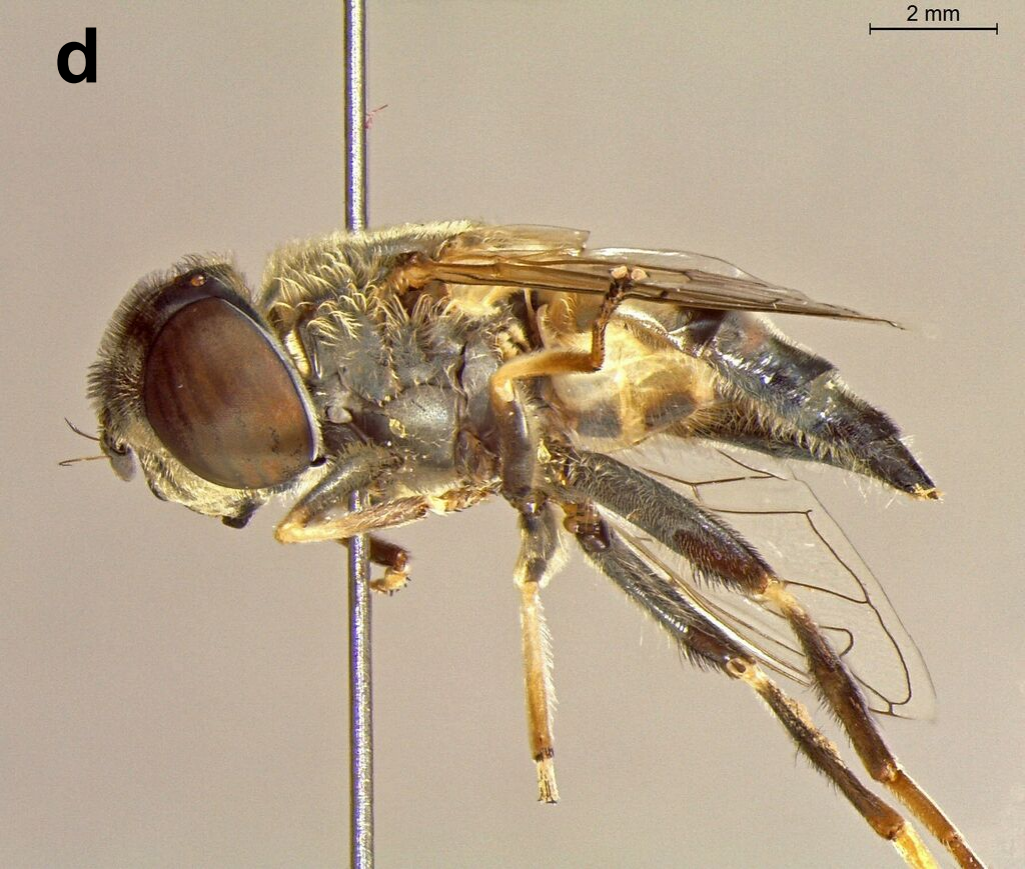
female in profile

**Figure 25a.**
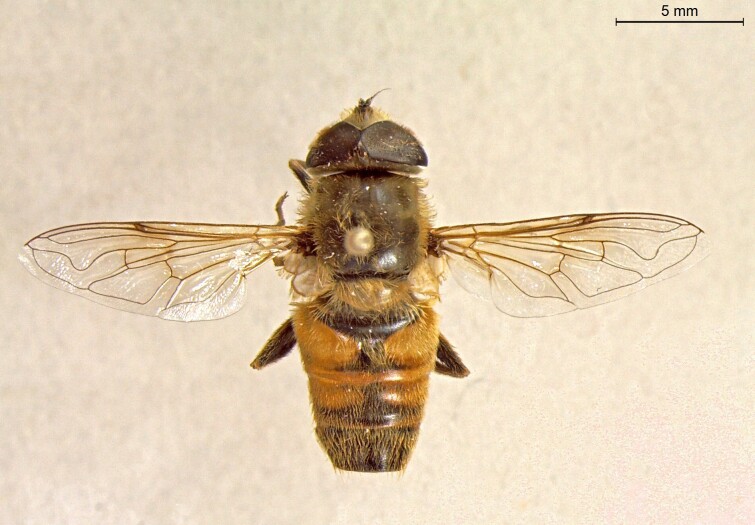
male in dorsal view

**Figure 25b.**
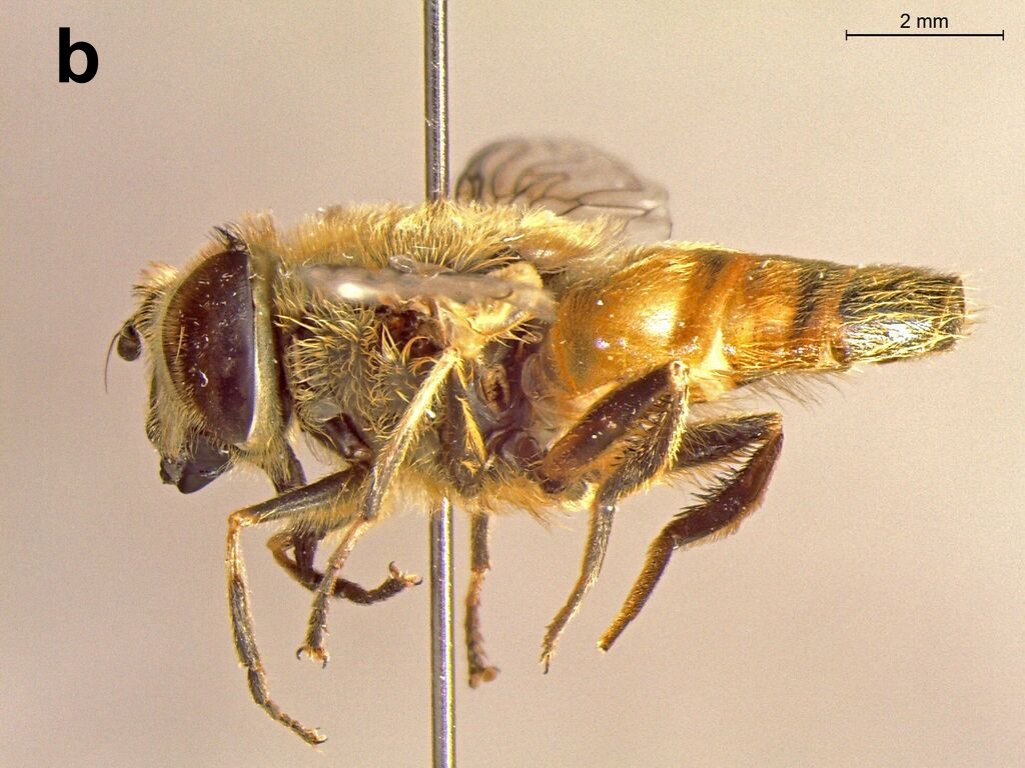
male in profile

**Figure 25c.**
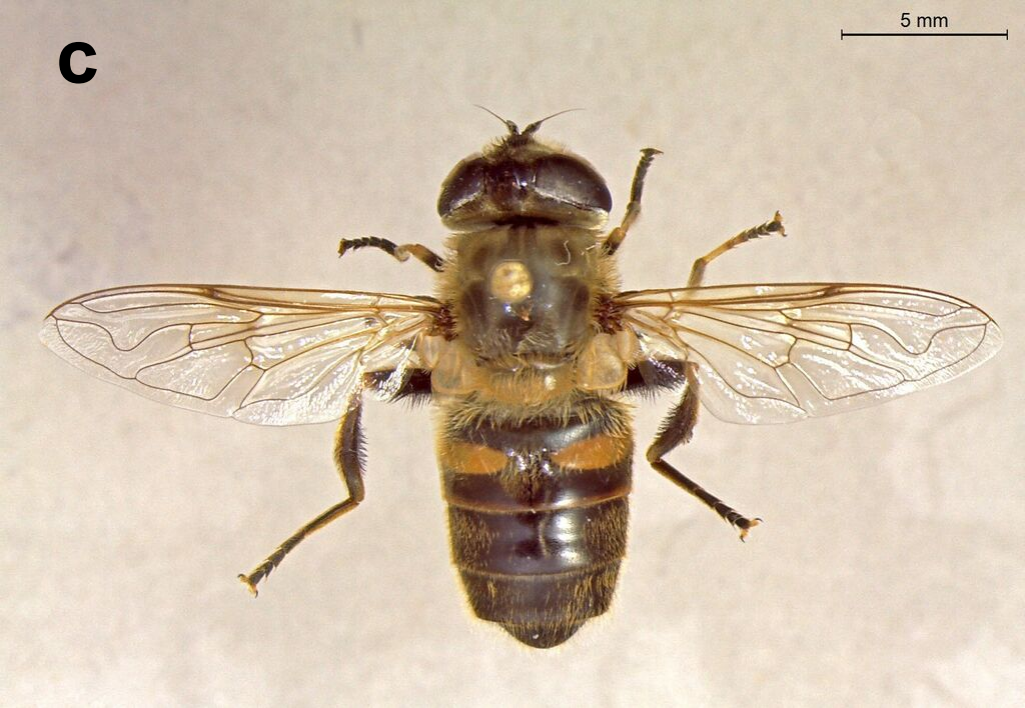
female in dorsal view

**Figure 25d.**
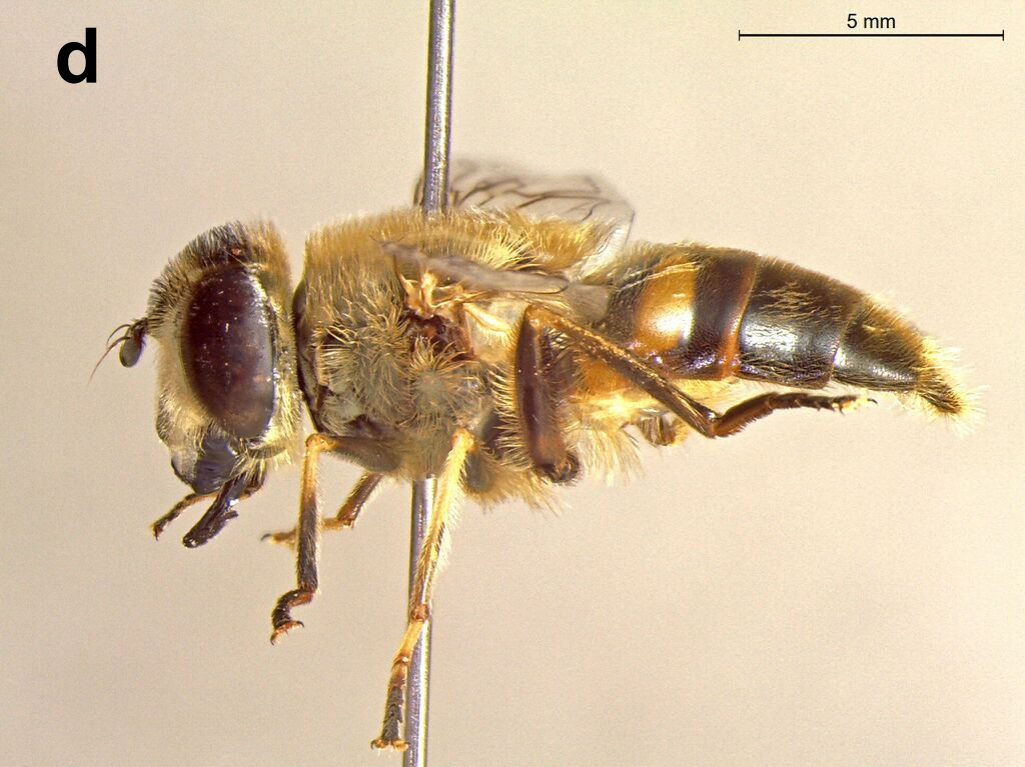
female in profile

**Figure 26a.**
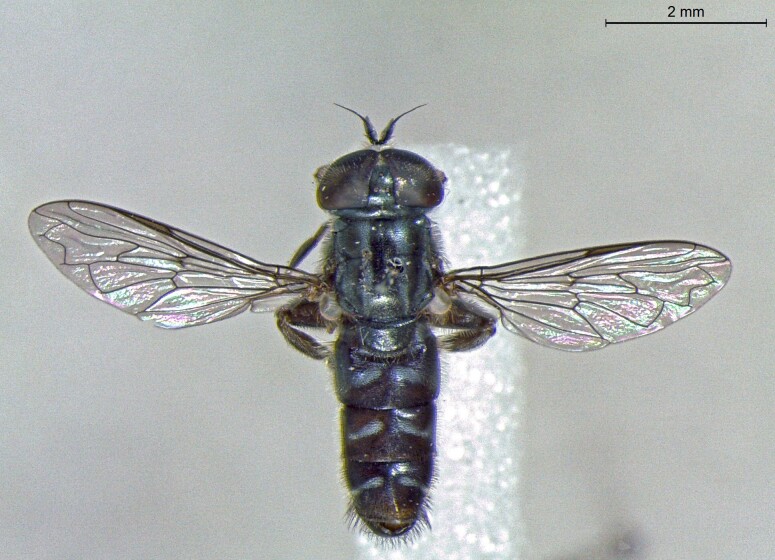
male in dorsal view

**Figure 26b.**
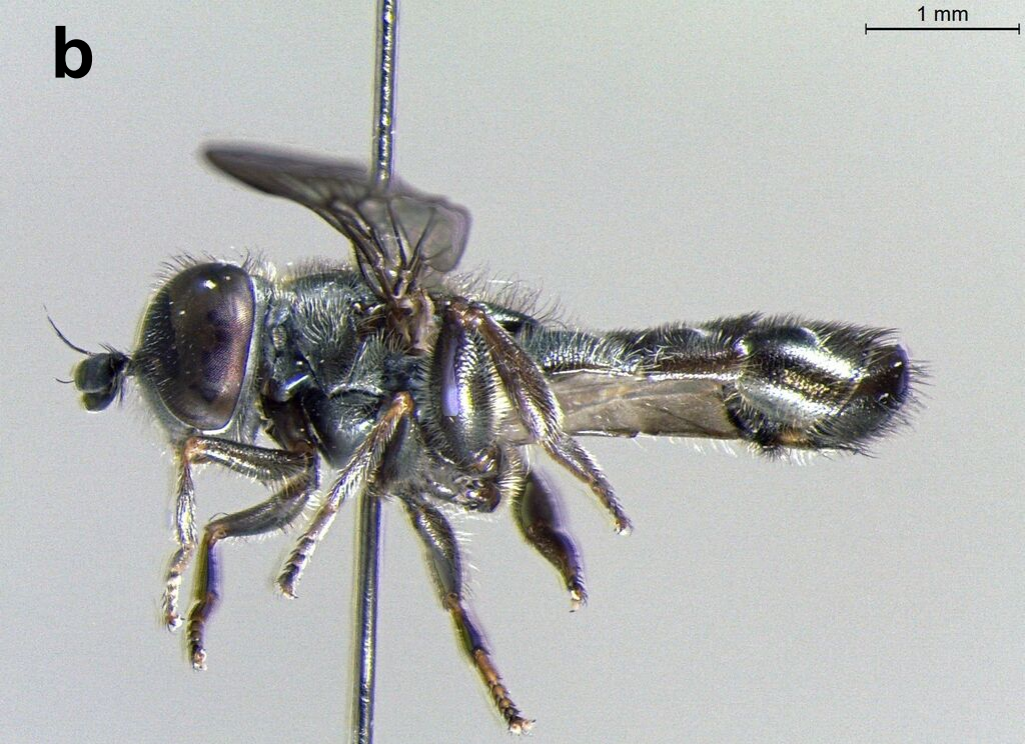
male in profile

**Figure 26c.**
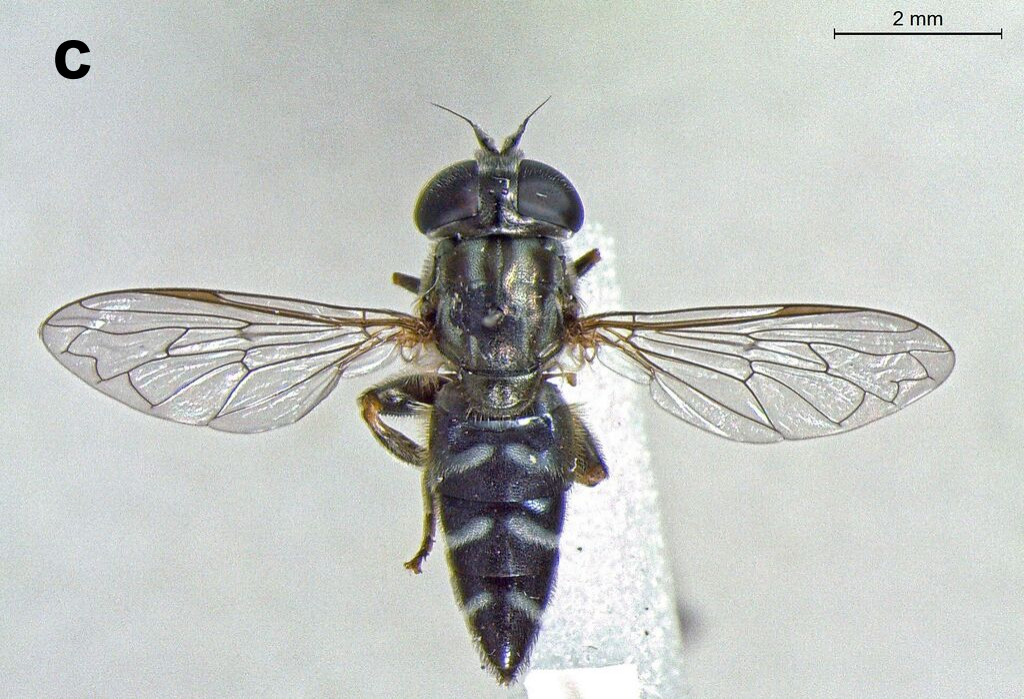
female in dorsal view

**Figure 26d.**
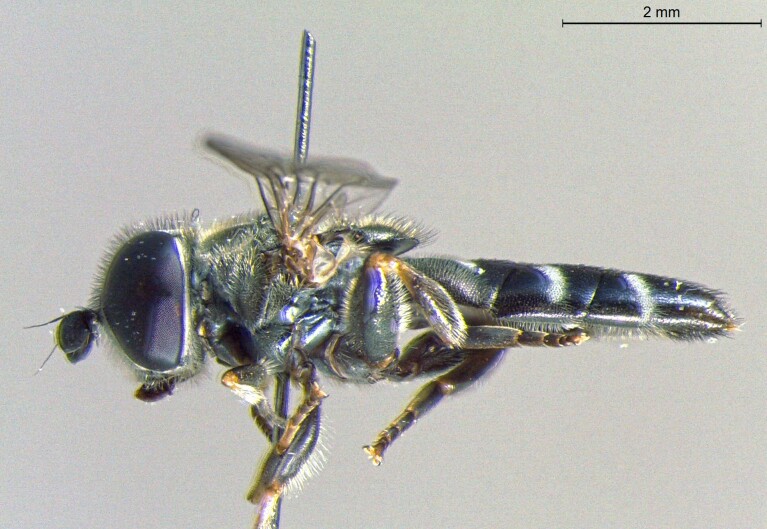
female in profile

**Figure 27a.**
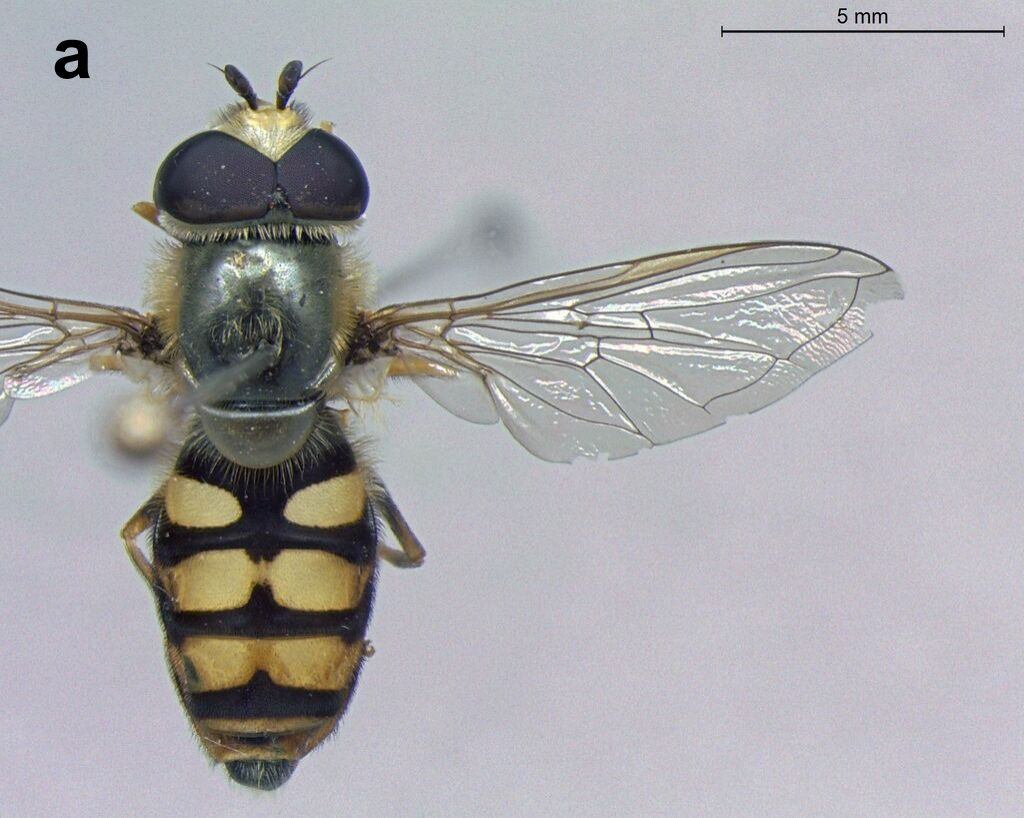
male in dorsal view

**Figure 27b.**
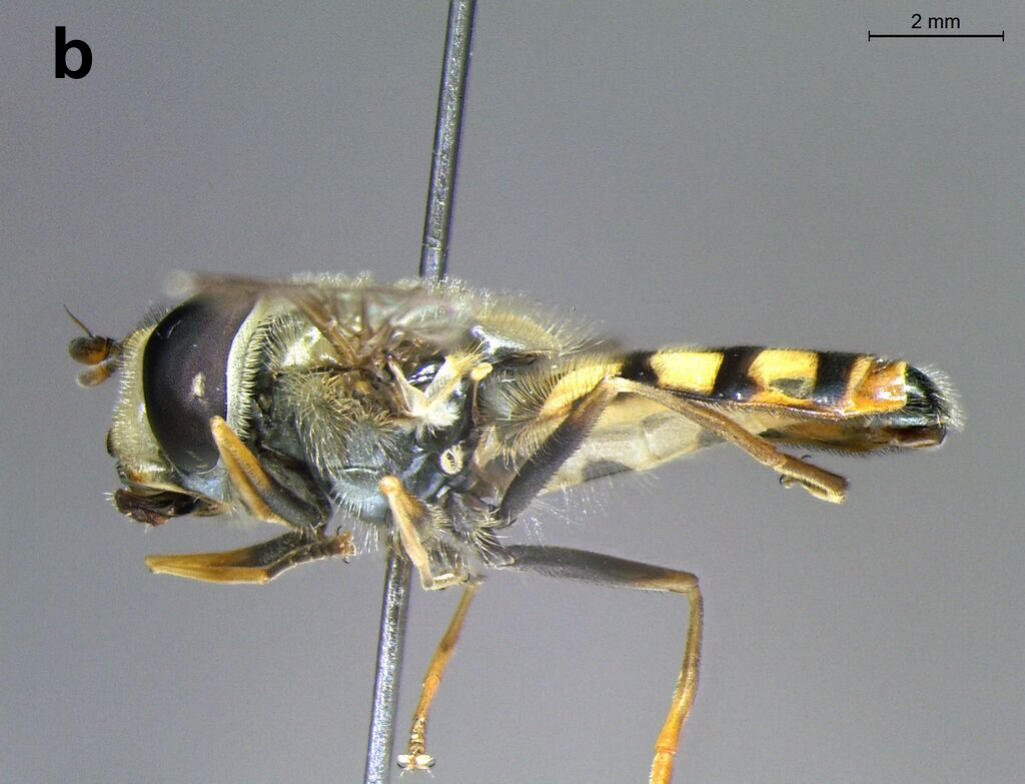
male in profile

**Figure 27c.**
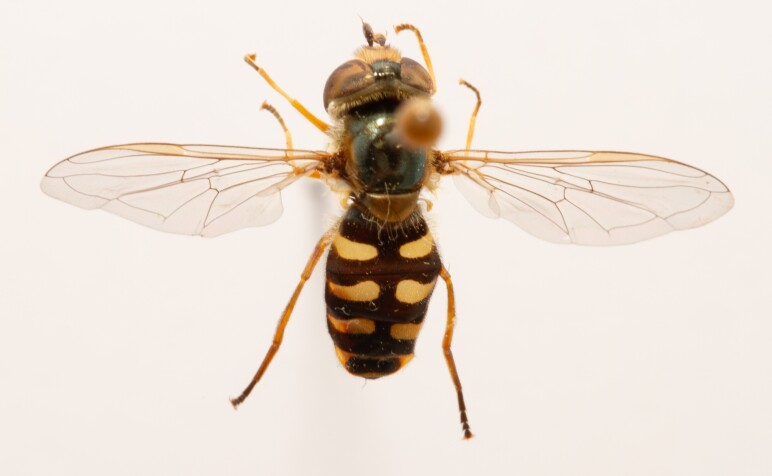
female in dorsal view

**Figure 27d.**
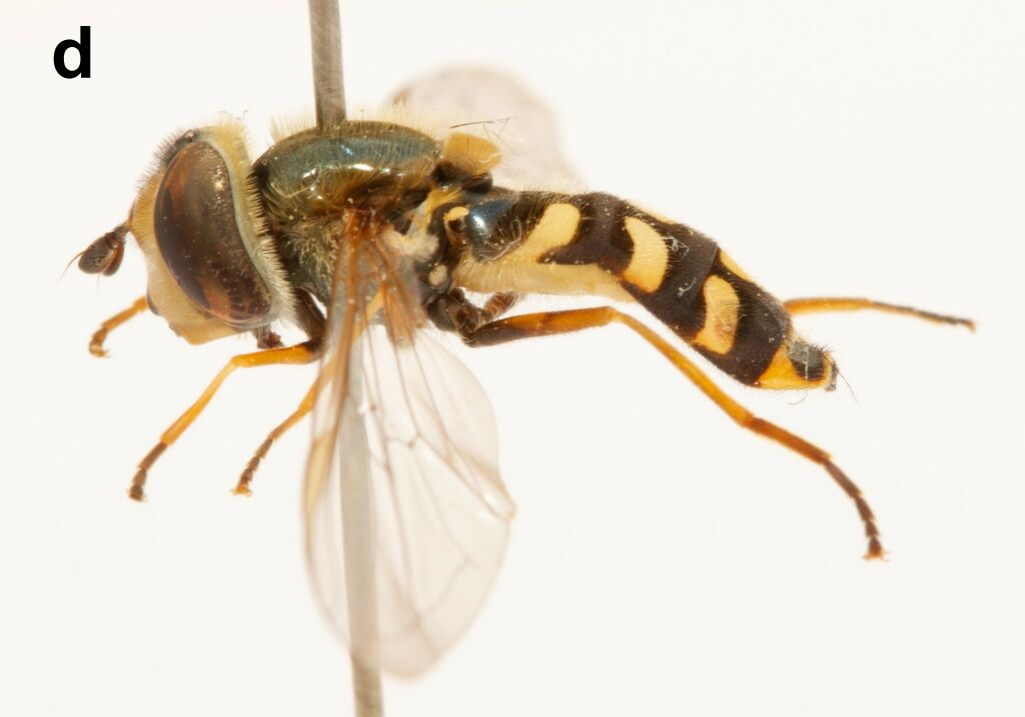
female in profile

**Figure 28a.**
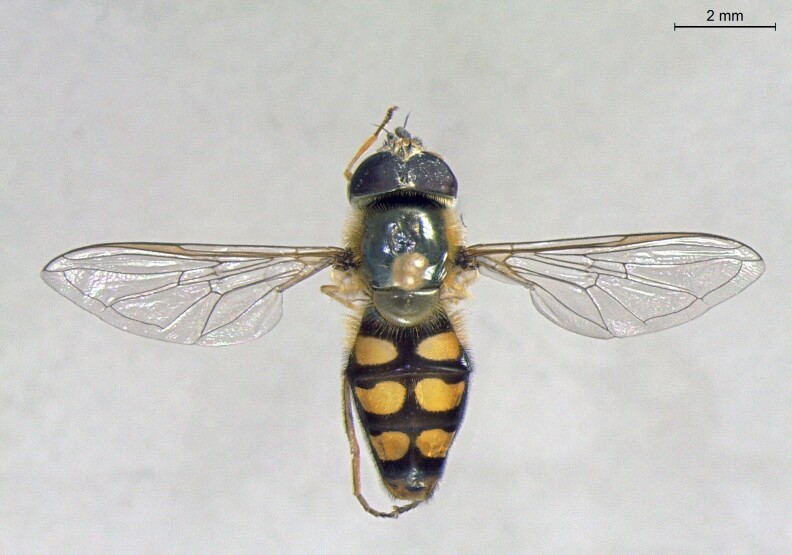
male in dorsal view

**Figure 28b.**
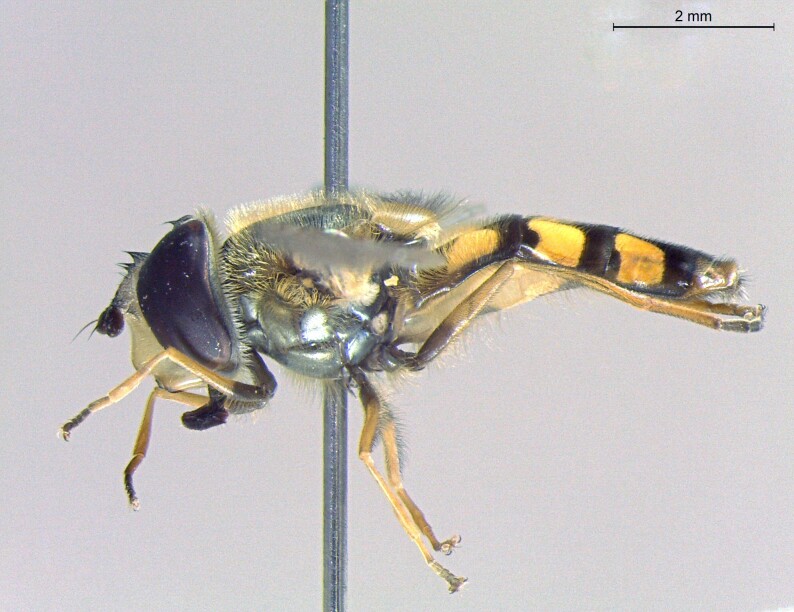
male in profile

**Figure 28c.**
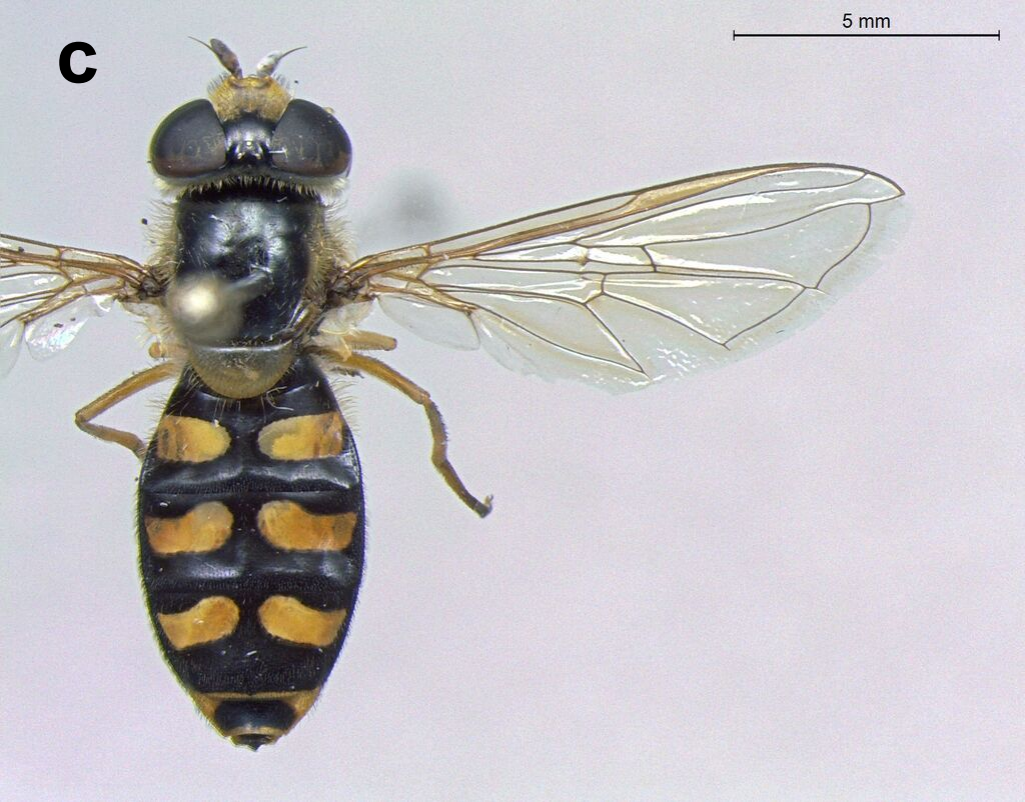
female in dorsal view

**Figure 28d.**
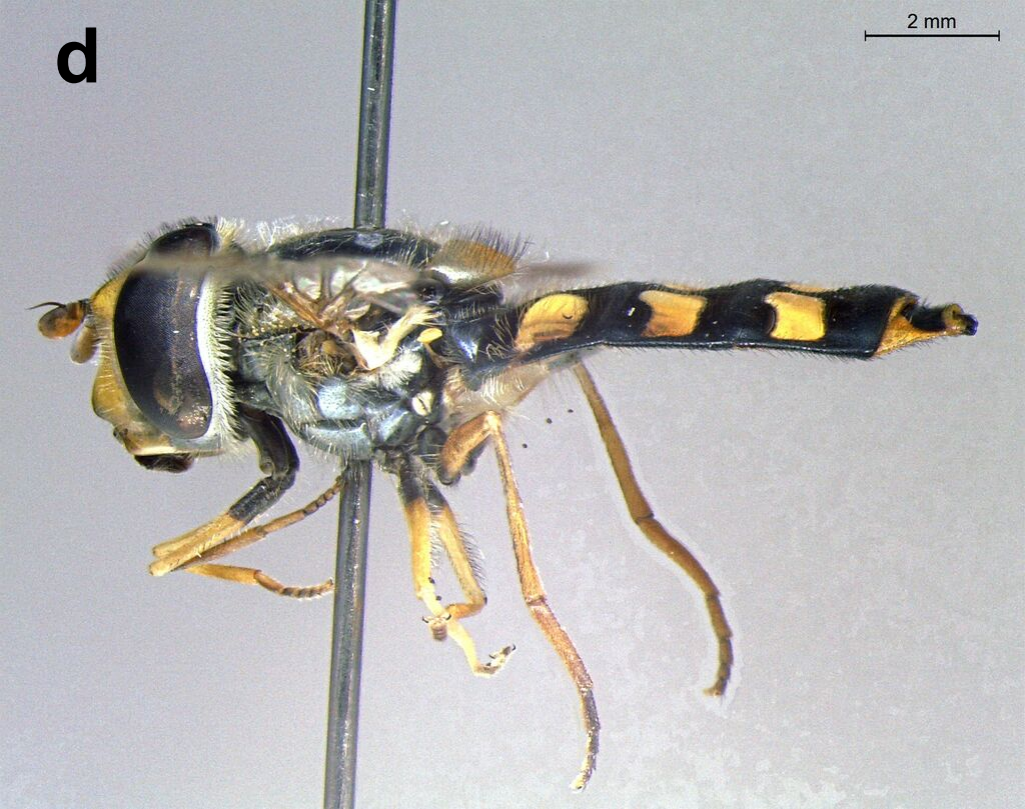
female in profile

**Figure 29a.**
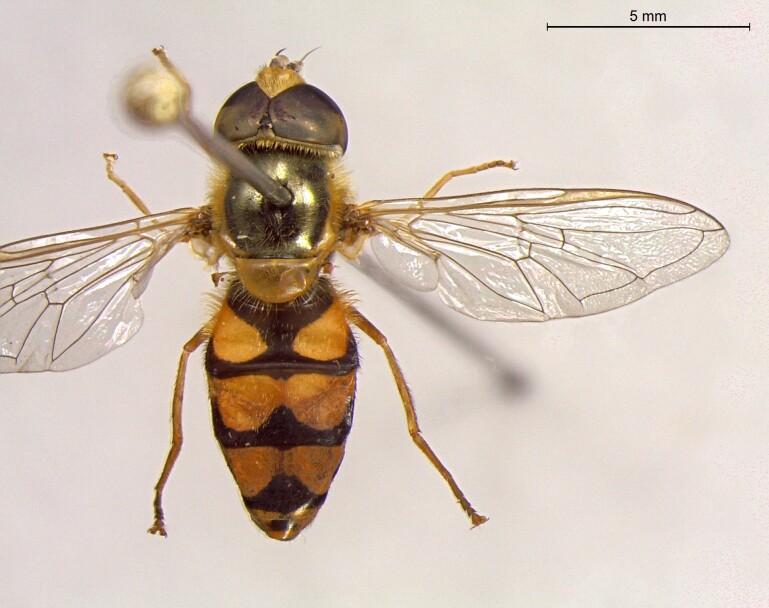
male in dorsal view

**Figure 29b.**
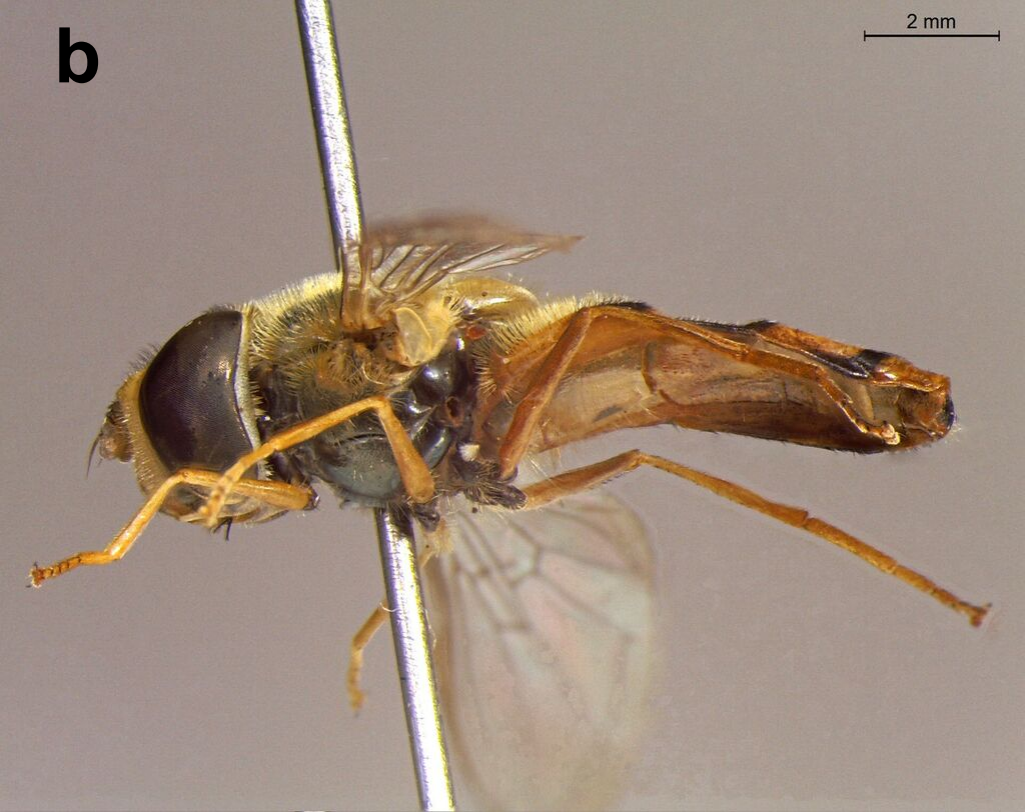
male in profile

**Figure 29c.**
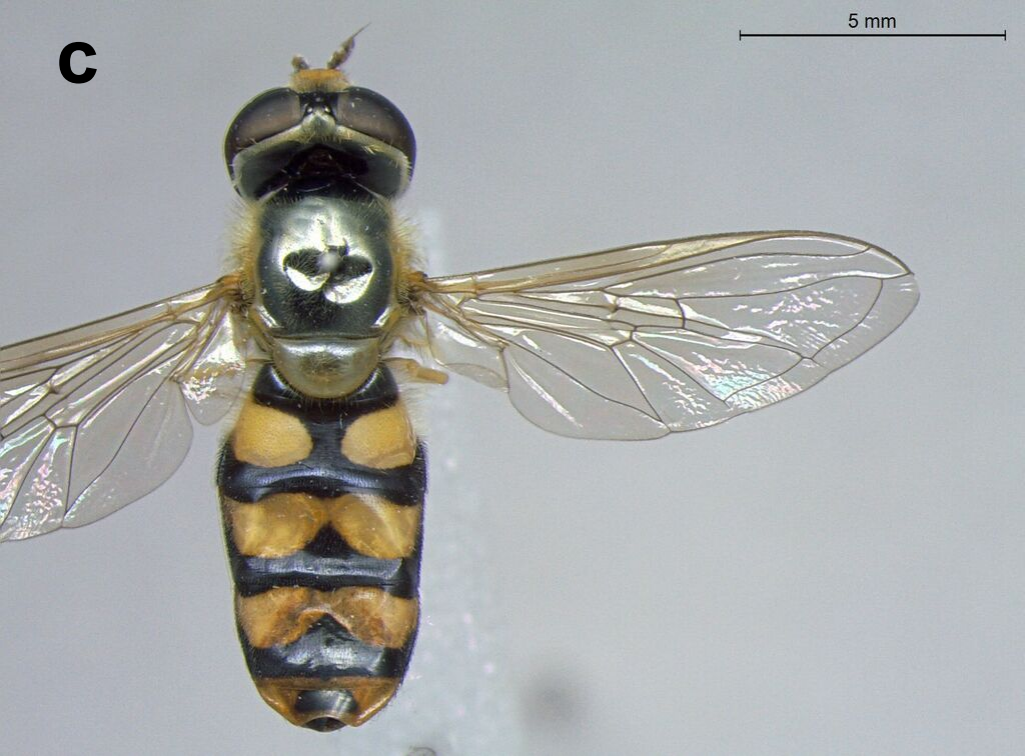
female in dorsal view

**Figure 29d.**
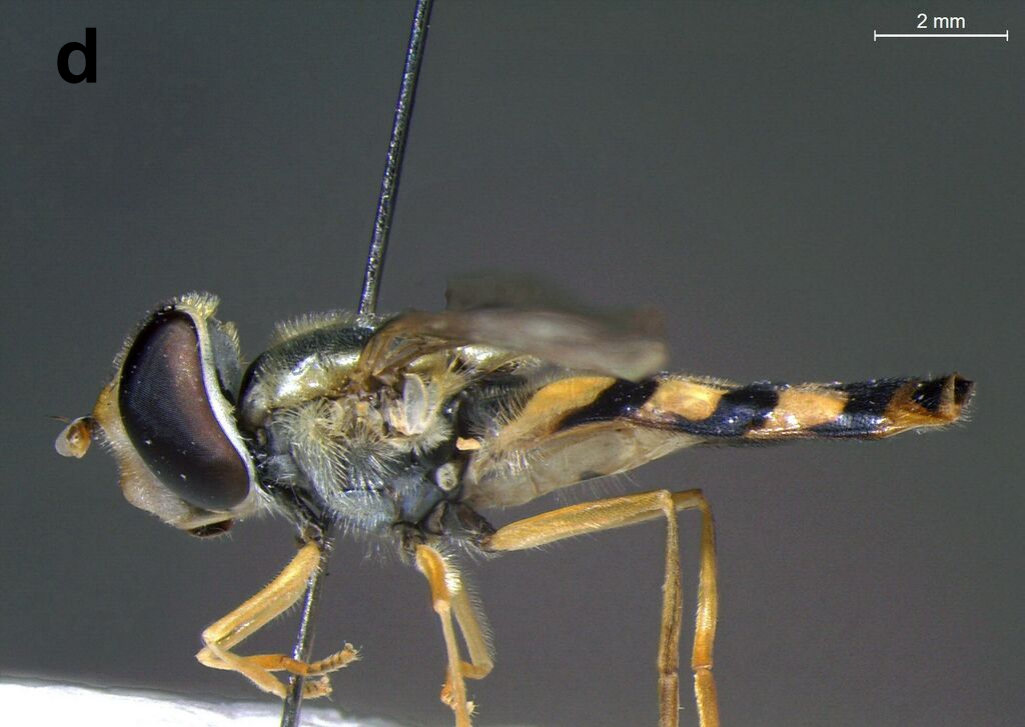
female in profile

**Figure 30a.**
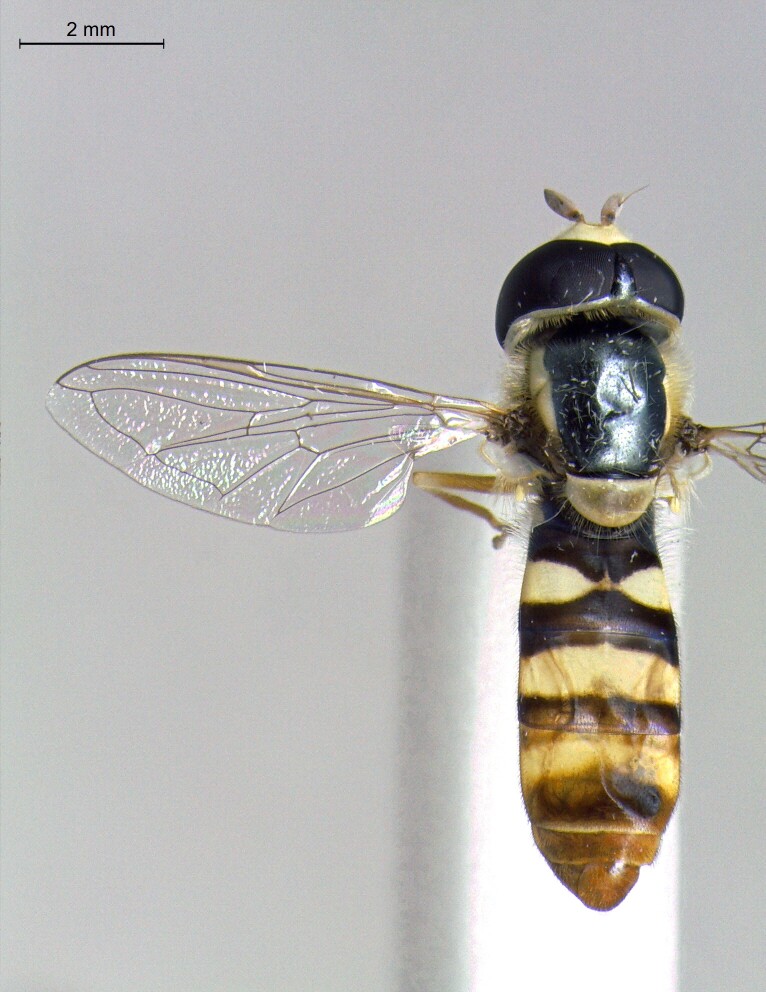
male in dorsal view

**Figure 30b.**
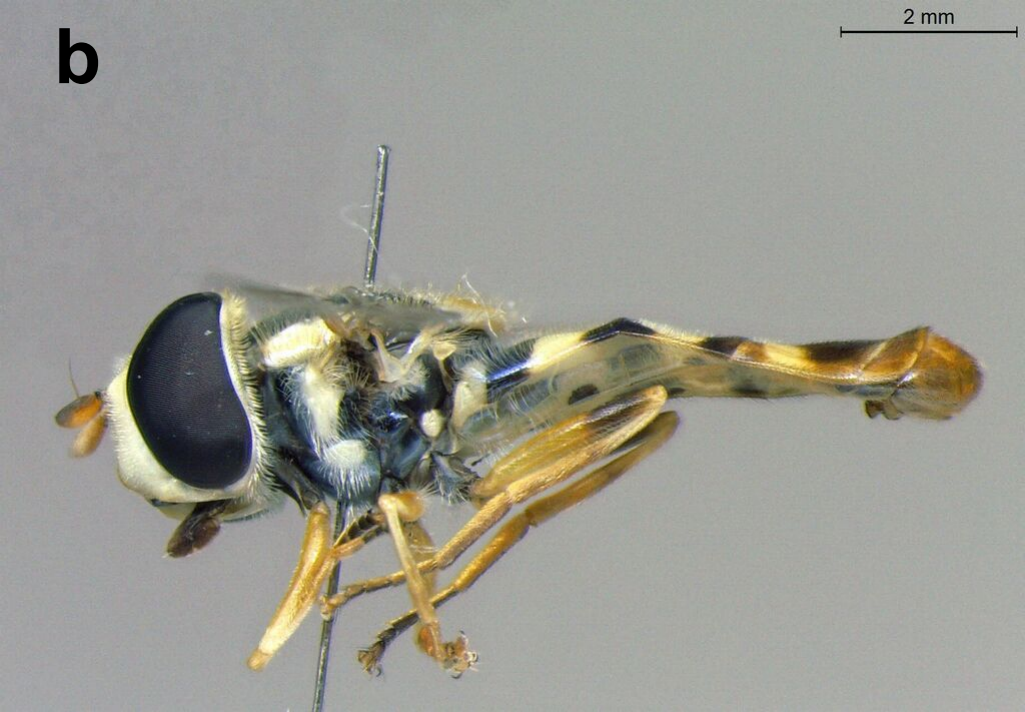
male in profile

**Figure 30c.**
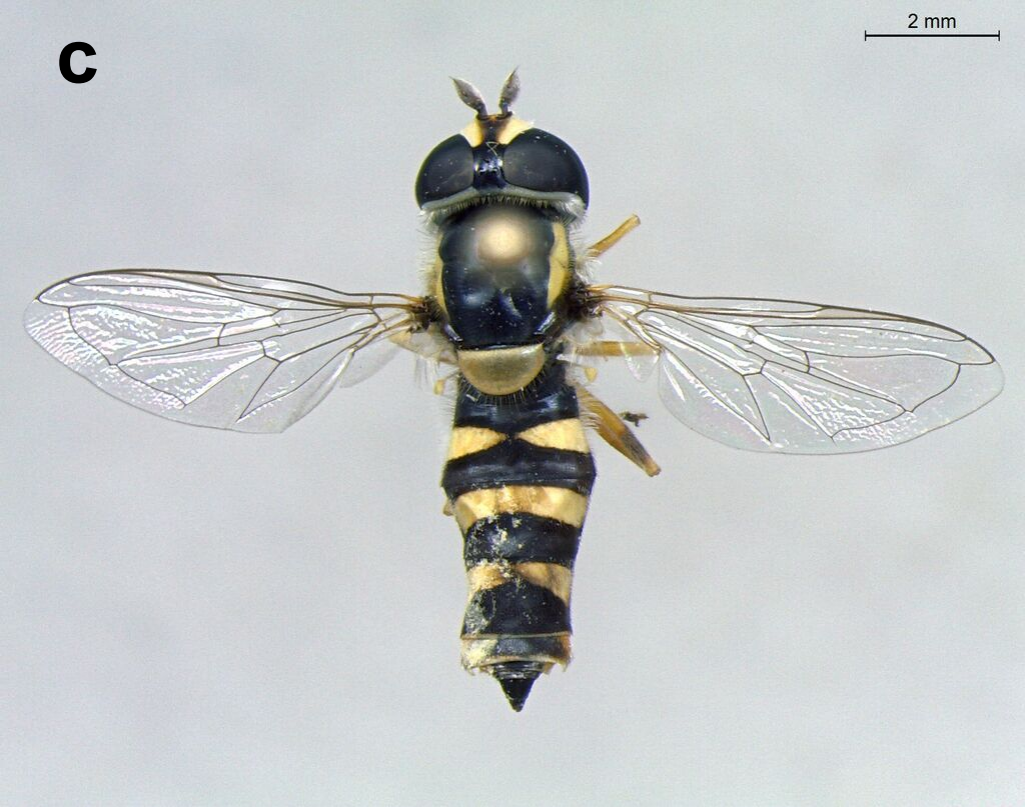
female in dorsal view

**Figure 30d.**
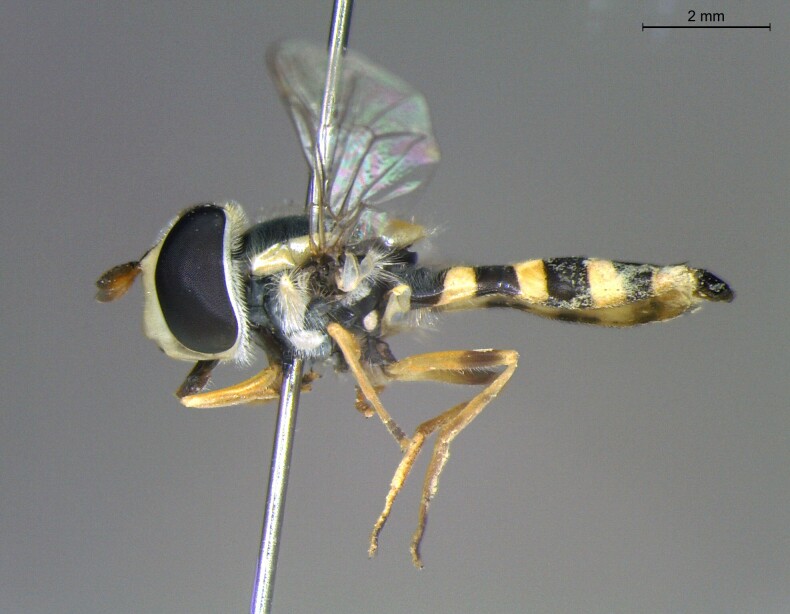
female in profile

**Figure 31a.**
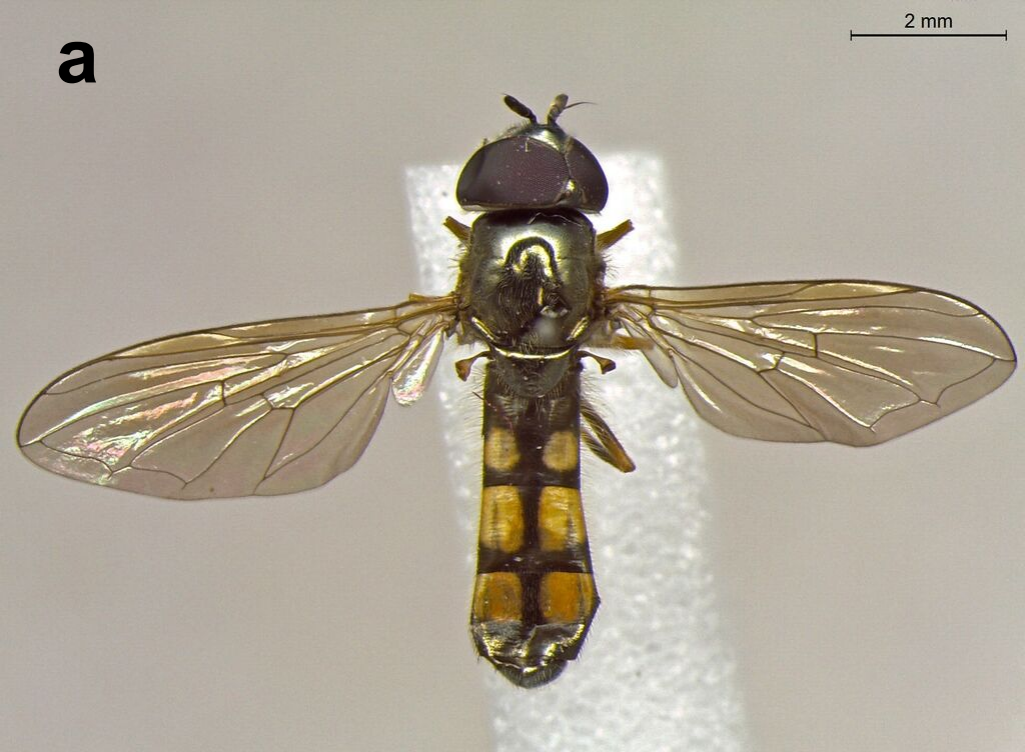
male in dorsal view

**Figure 31b.**
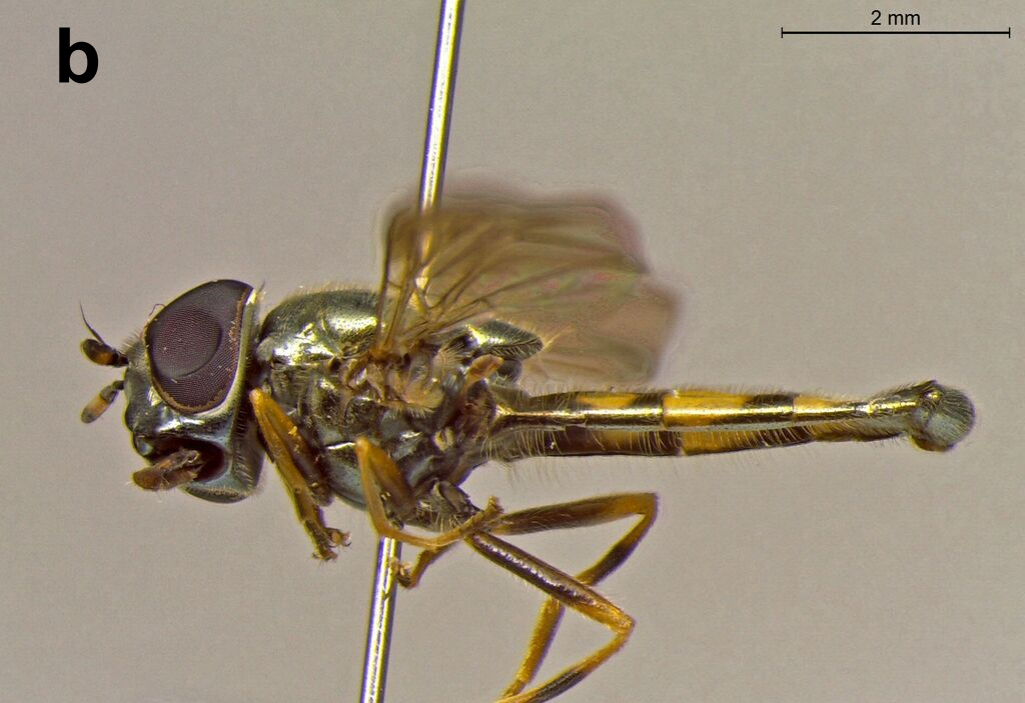
male in profile

**Figure 31c.**
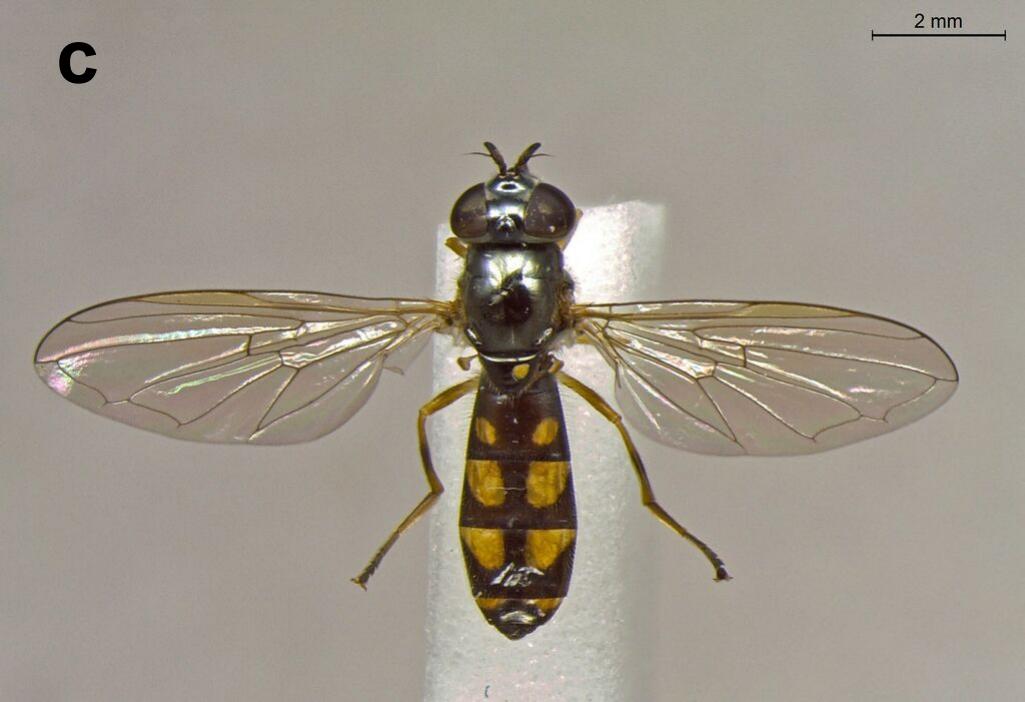
female in dorsal view

**Figure 31d.**
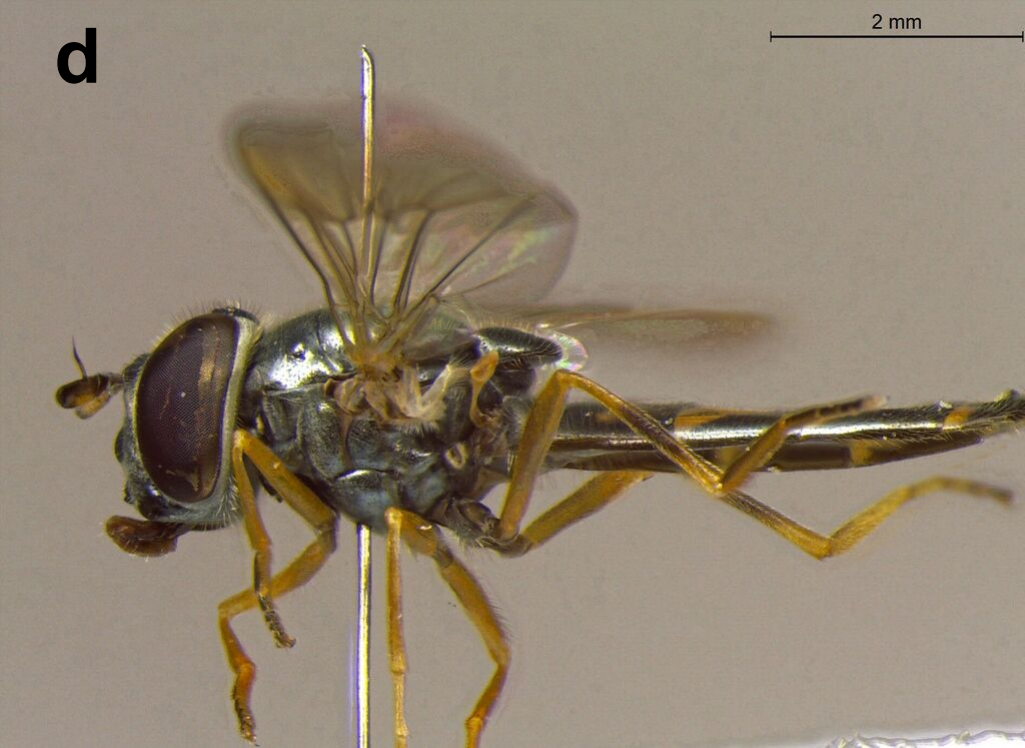
female in profile

**Figure 32a.**
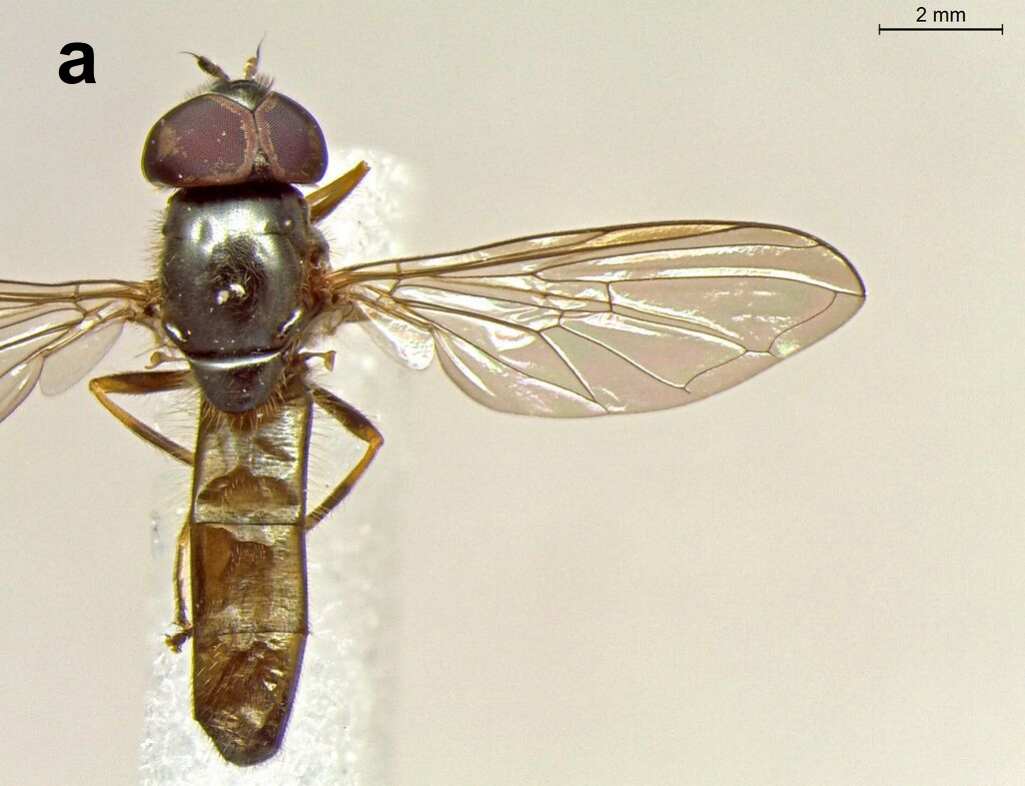
male in dorsal view

**Figure 32b.**
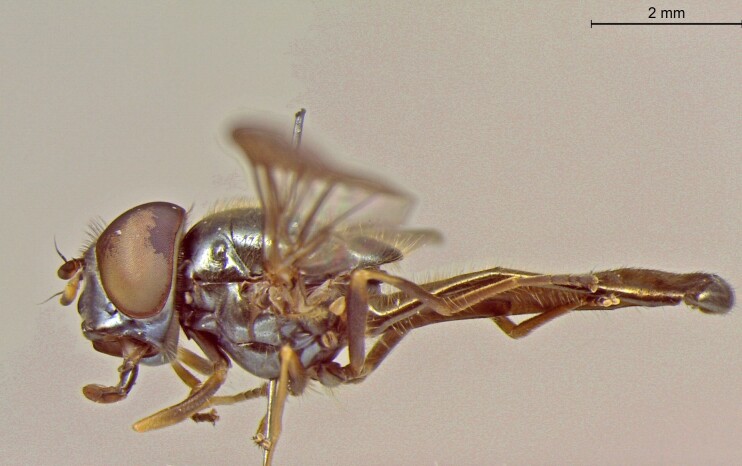
male in profile

**Figure 32c.**
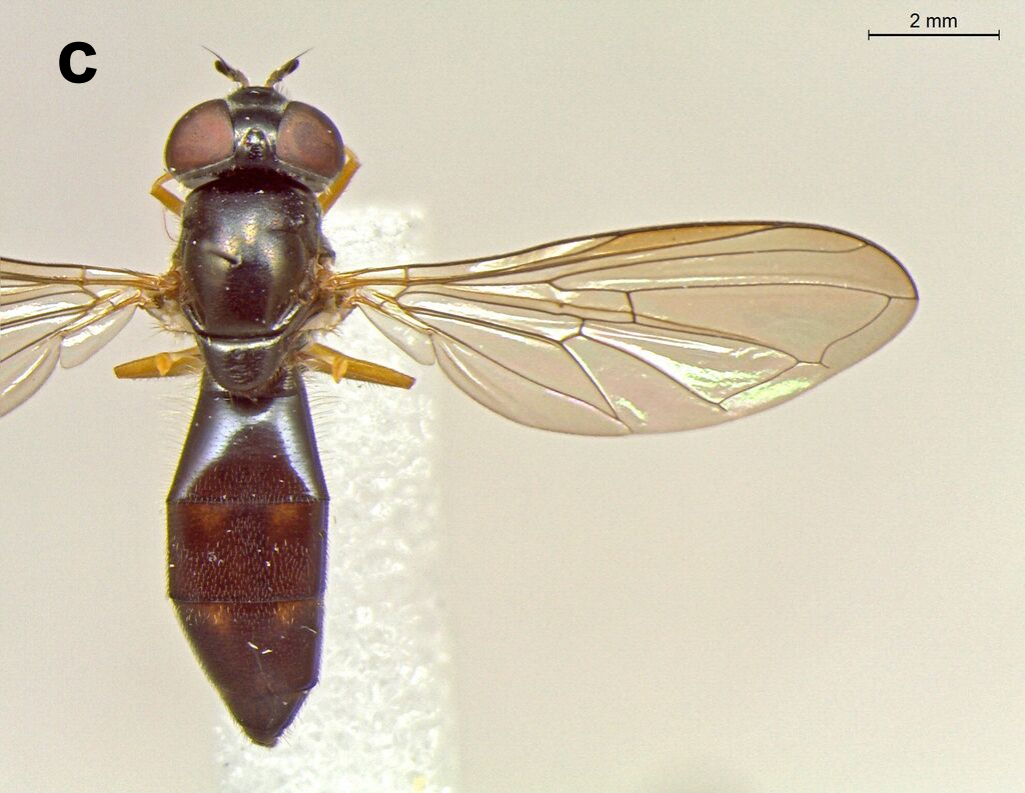
female in dorsal view

**Figure 32d.**
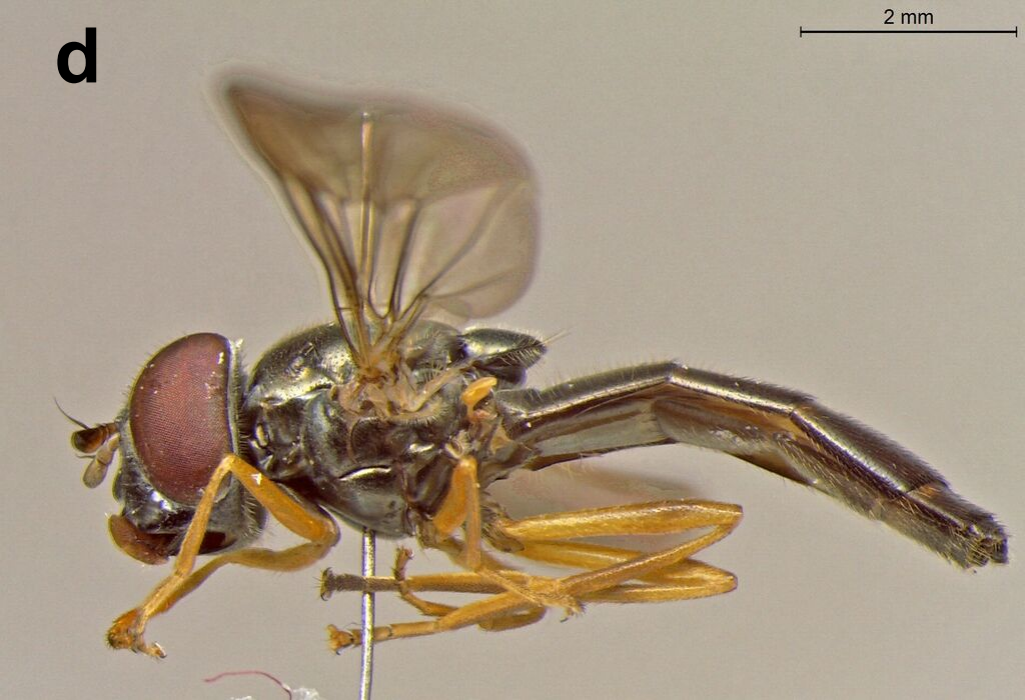
female in profile

**Figure 33a.**
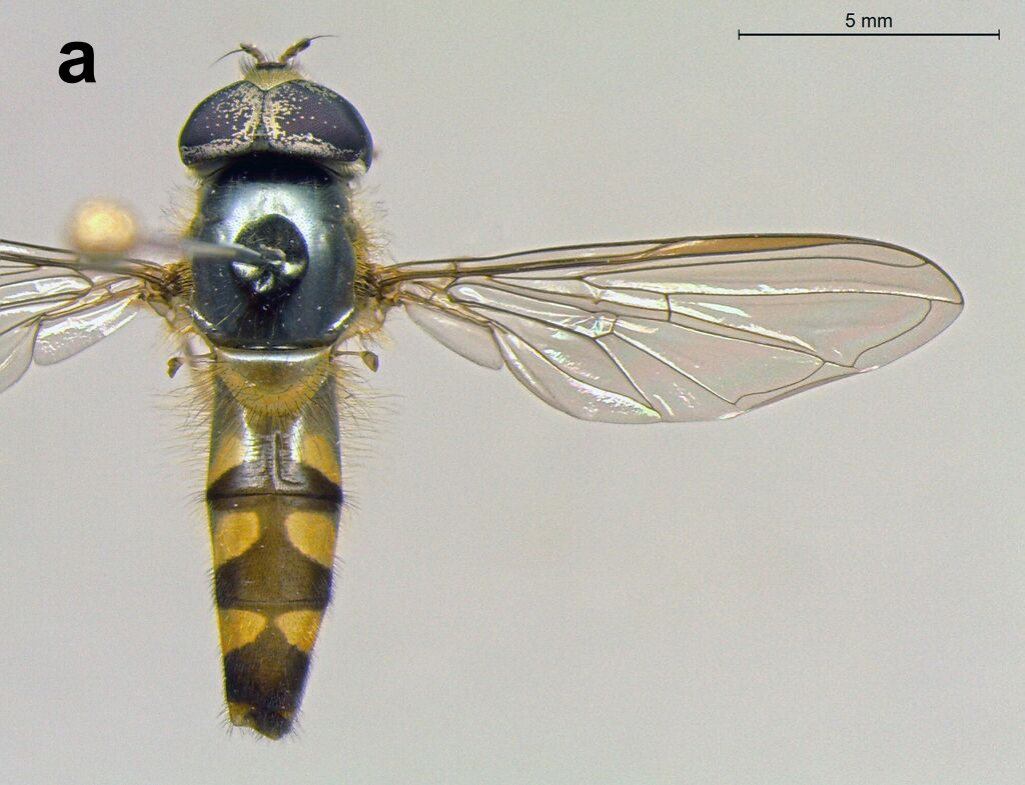
male in dorsal view

**Figure 33b.**
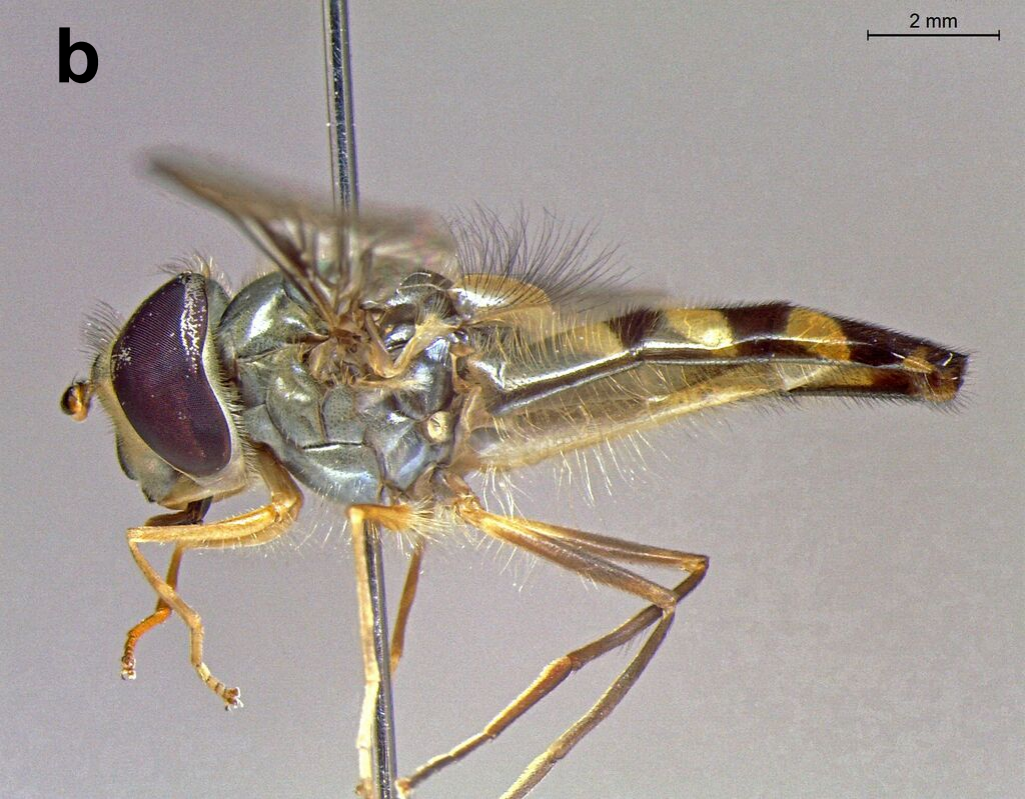
male in profile

**Figure 33c.**
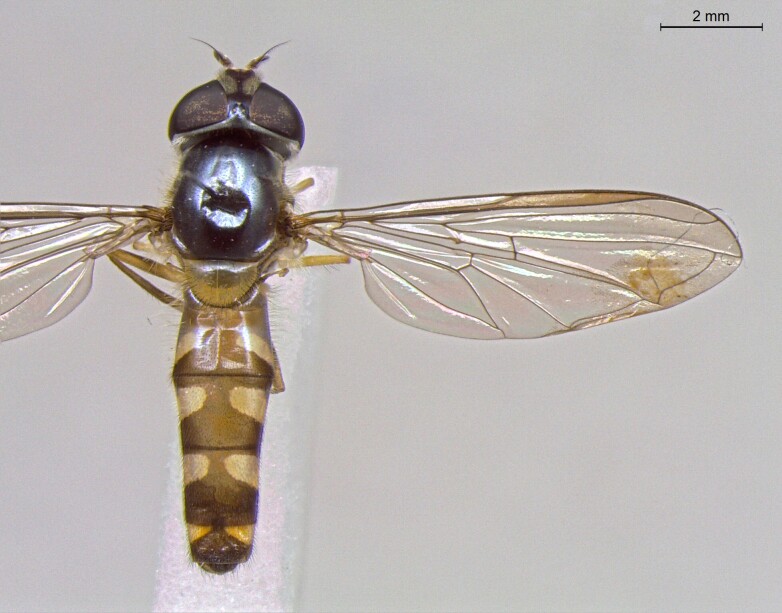
female in dorsal view

**Figure 33d.**
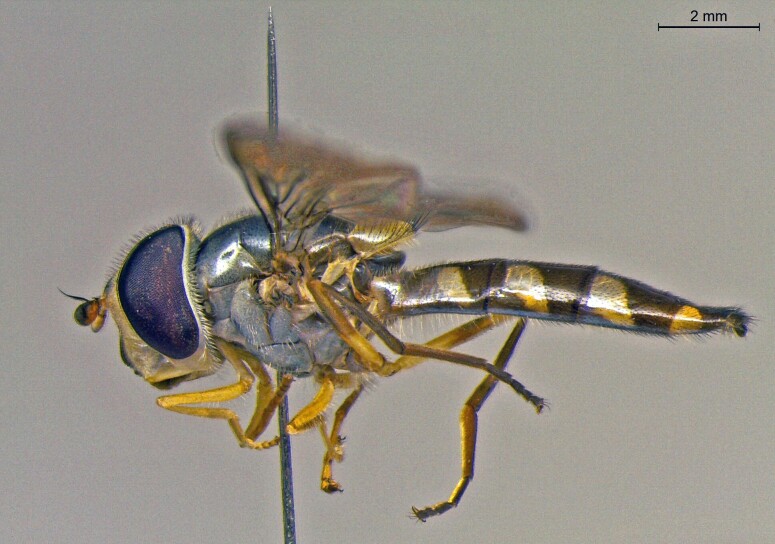
female in profile

**Figure 34a.**
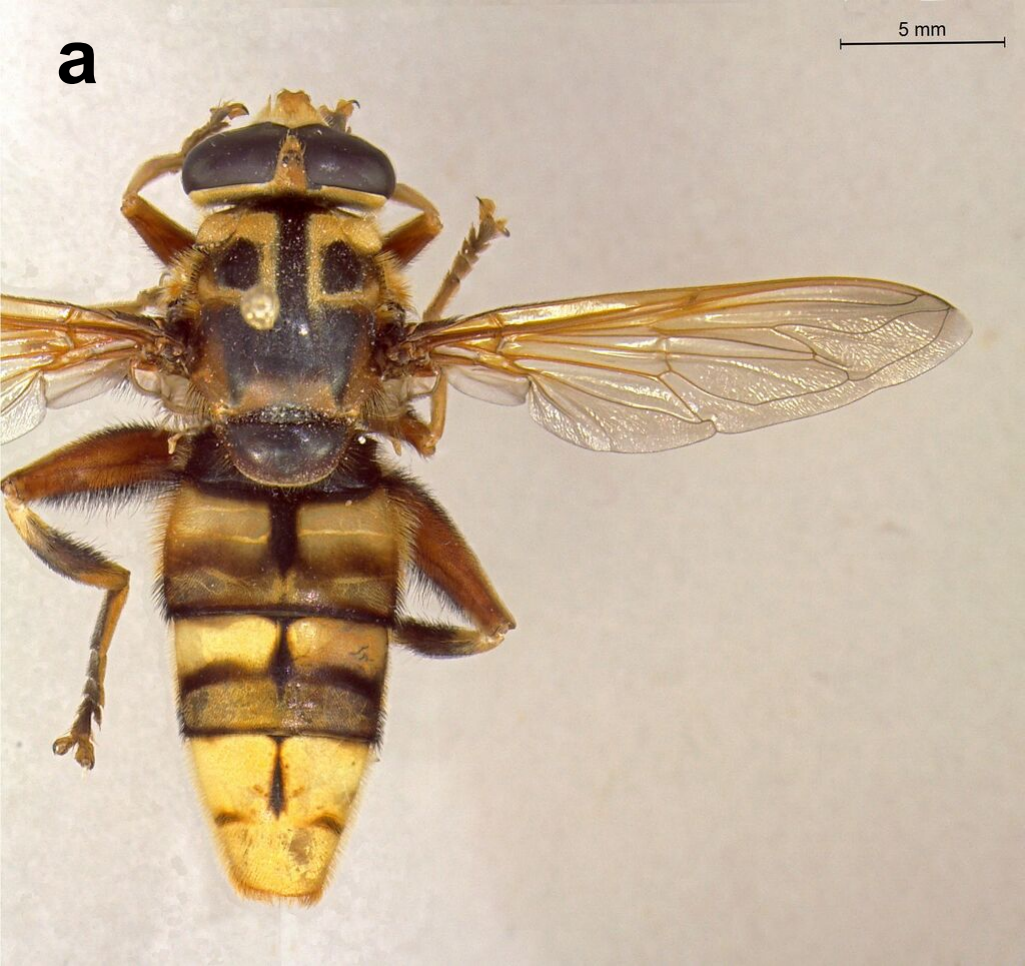
male in dorsal view

**Figure 34b.**
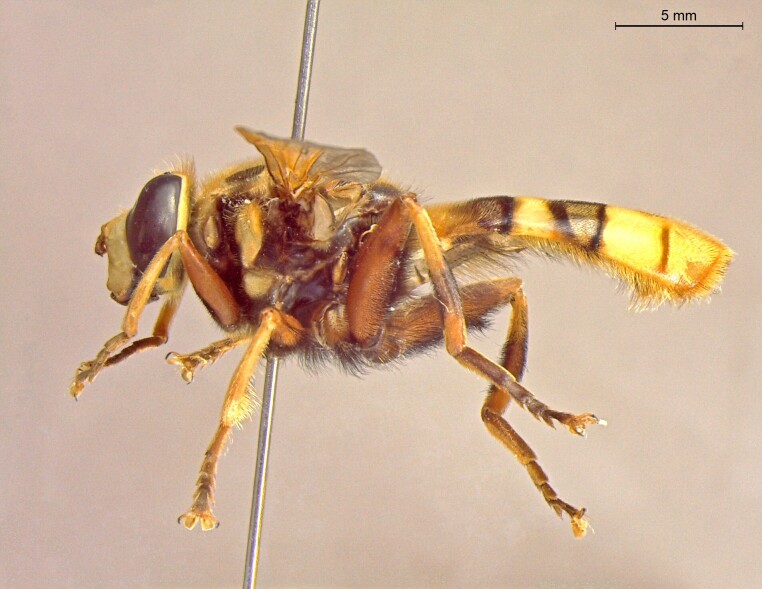
male in profile

**Figure 34c.**
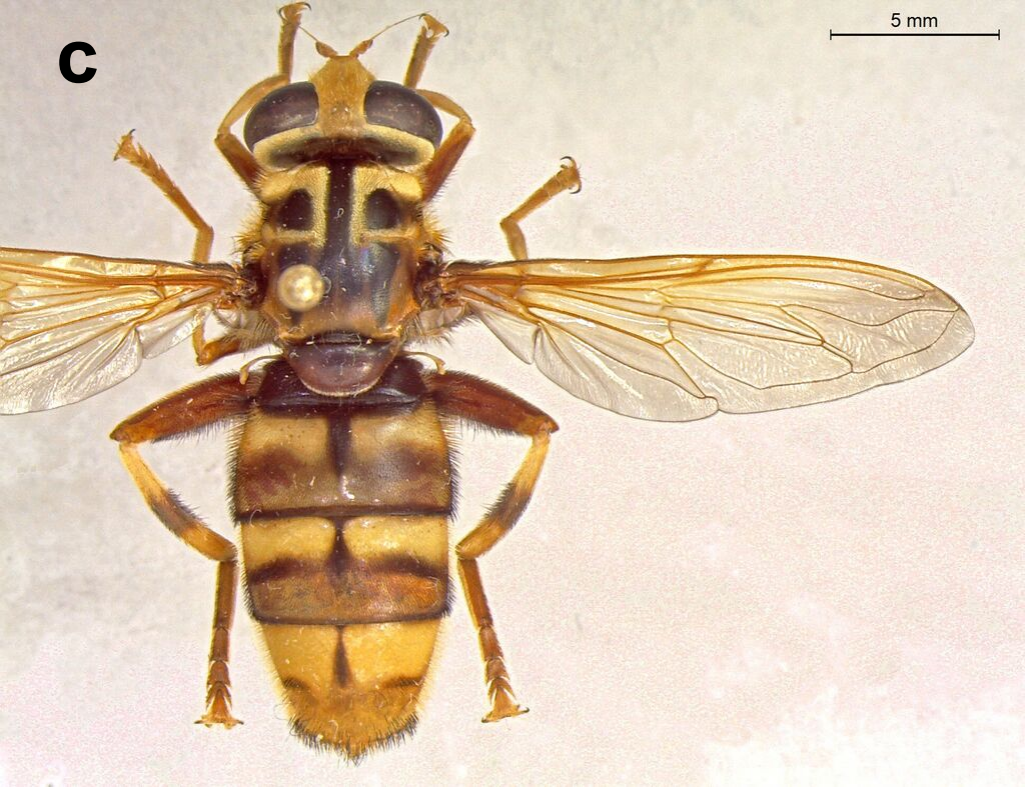
female in dorsal view

**Figure 34d.**
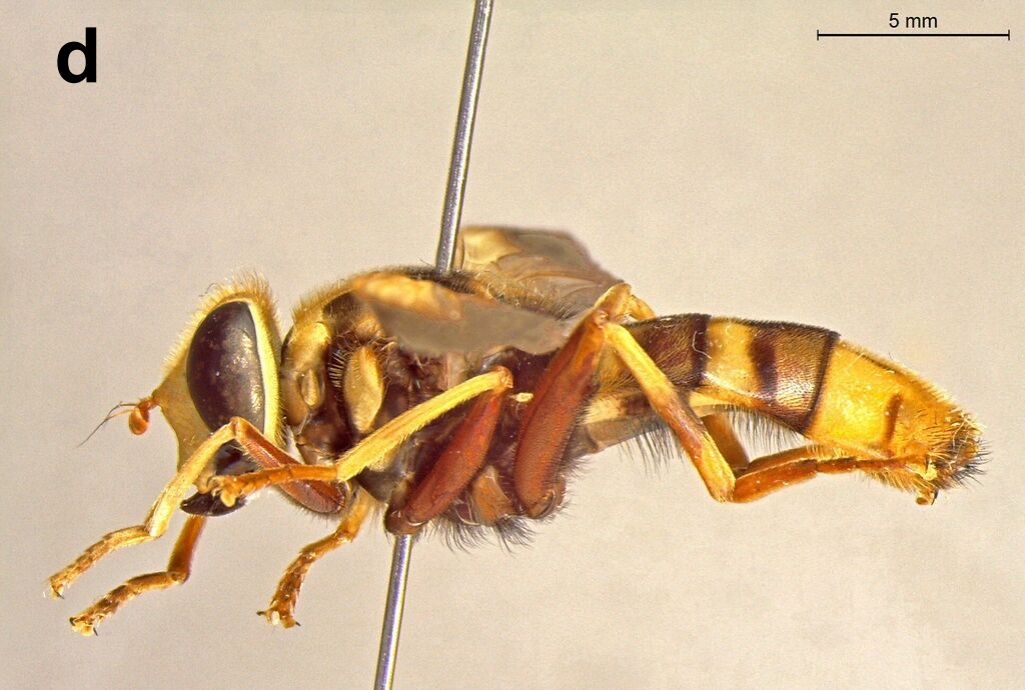
female in profile

**Figure 35a.**
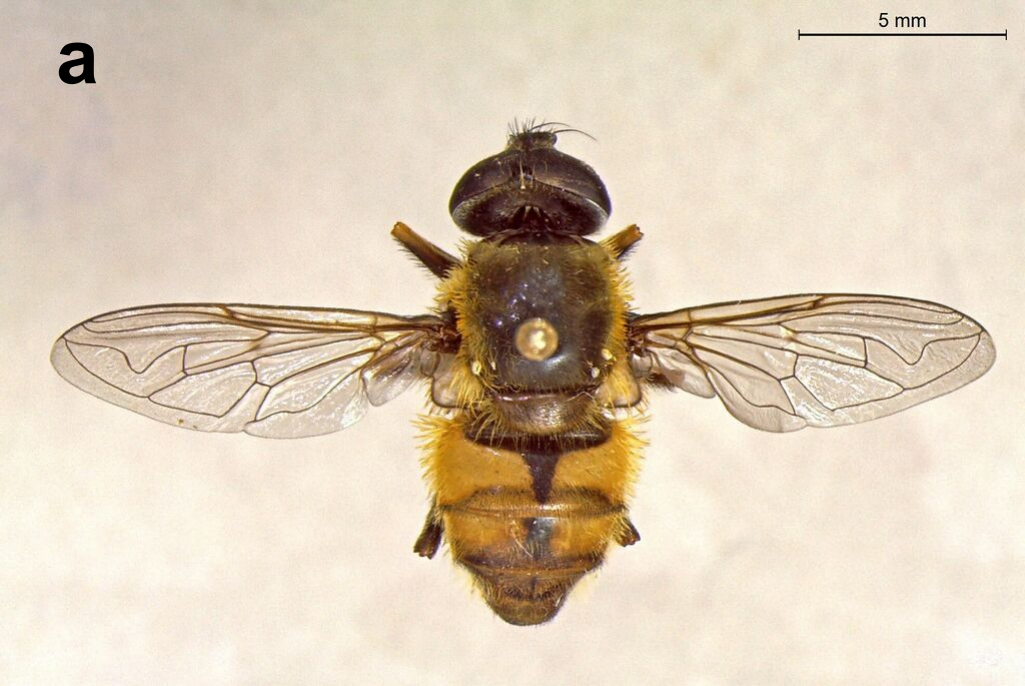
male in dorsal view

**Figure 35b.**
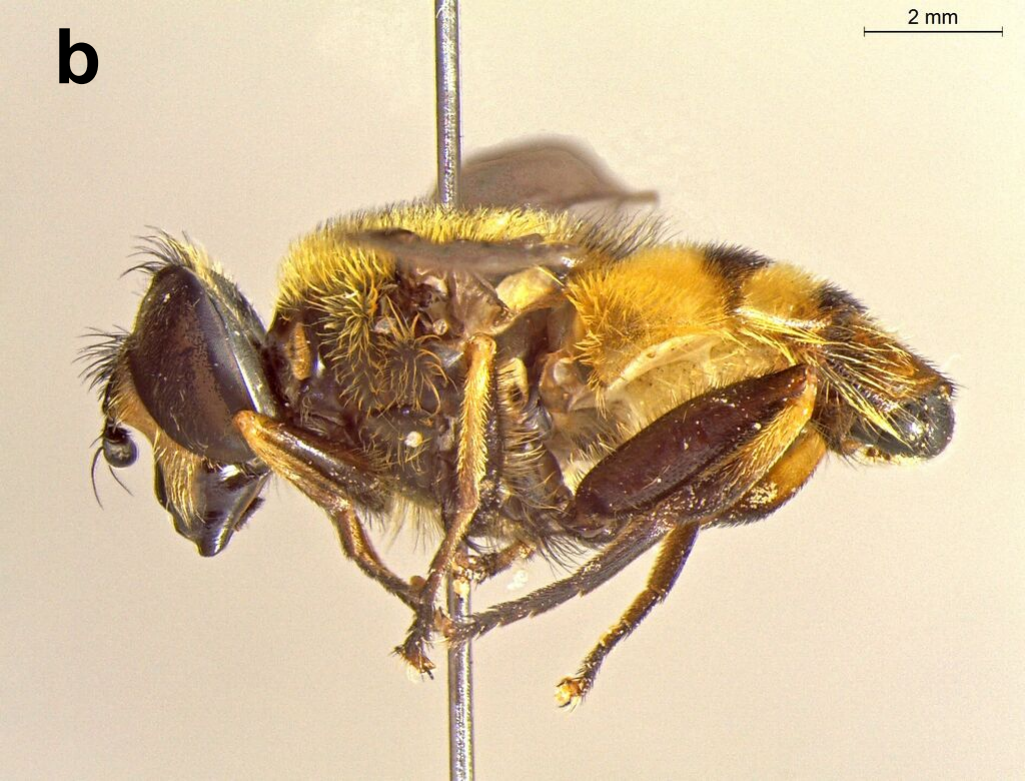
male in profile

**Figure 35c.**
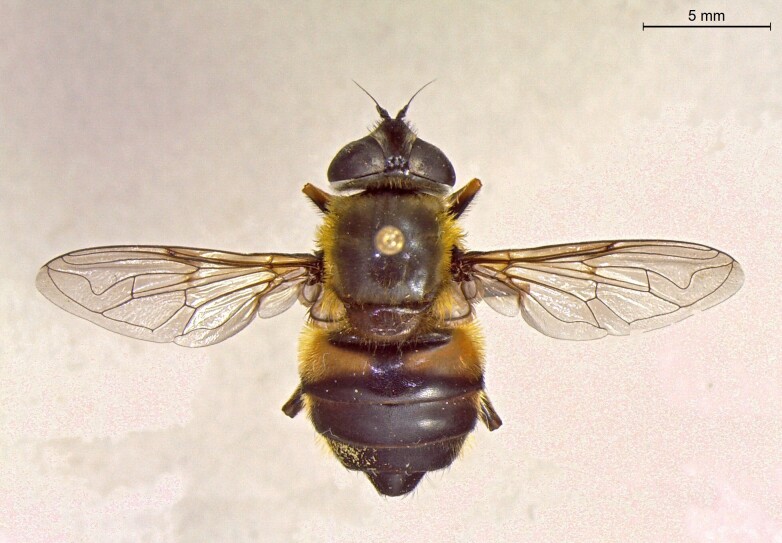
female in dorsal view

**Figure 35d.**
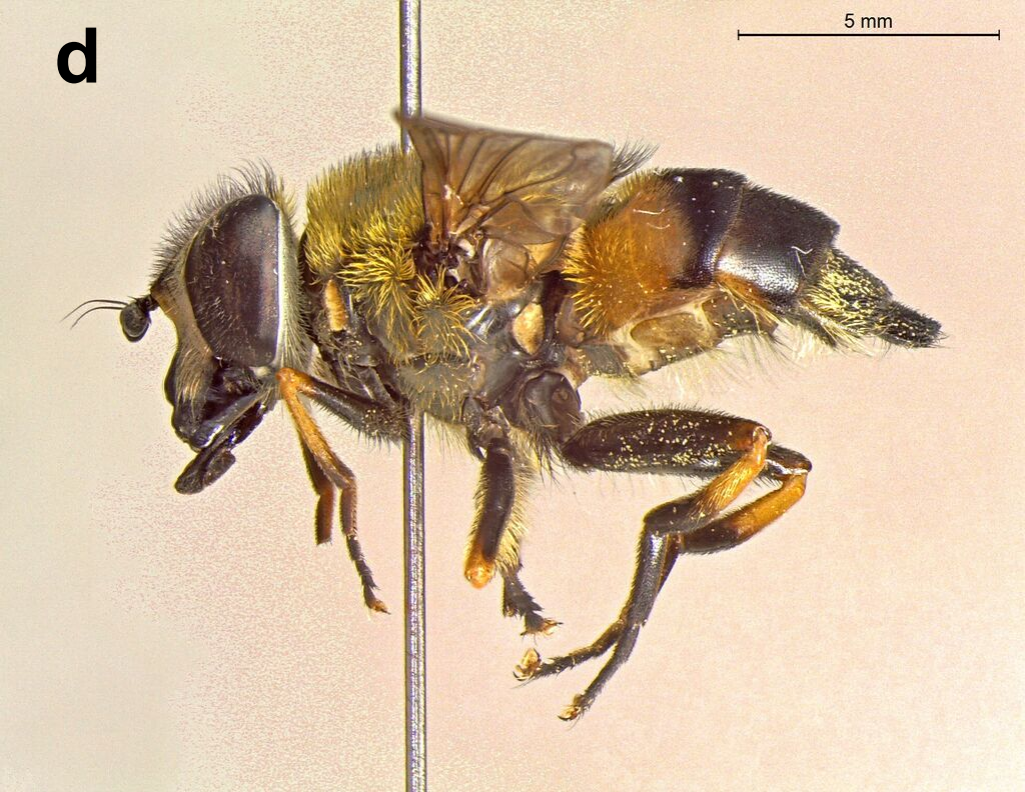
female in profile

**Figure 36a.**
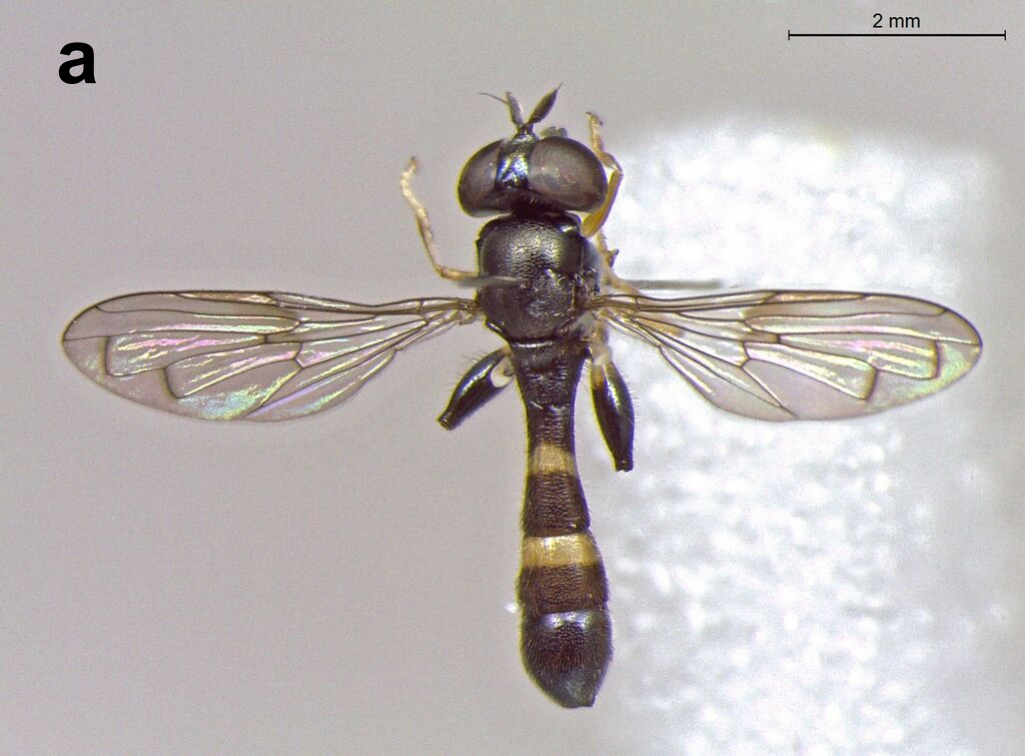
male in dorsal view

**Figure 36b.**
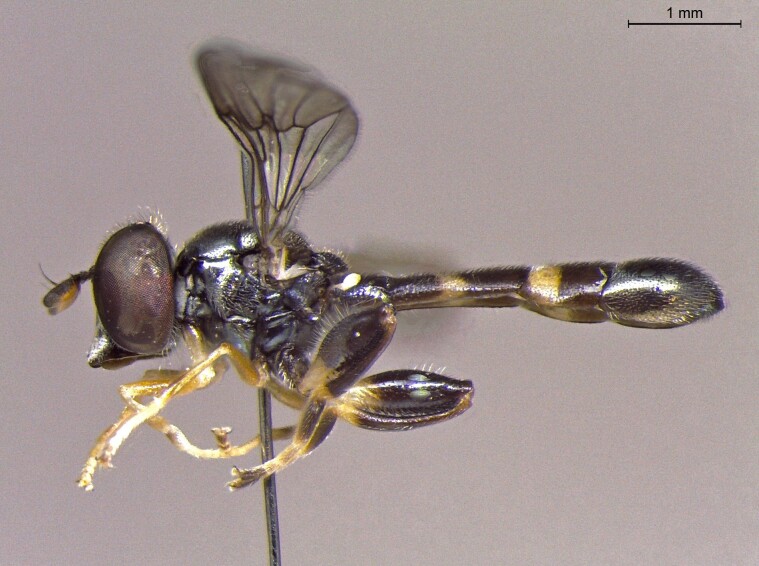
male in profile

**Figure 36c.**
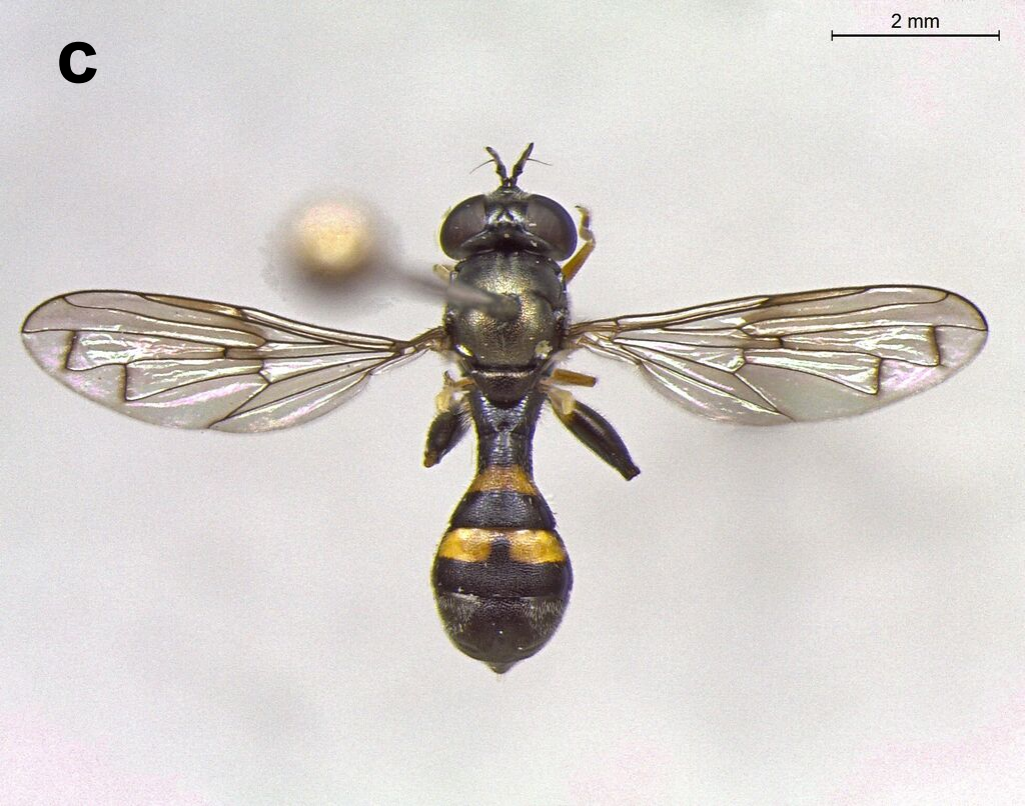
female in dorsal view

**Figure 36d.**
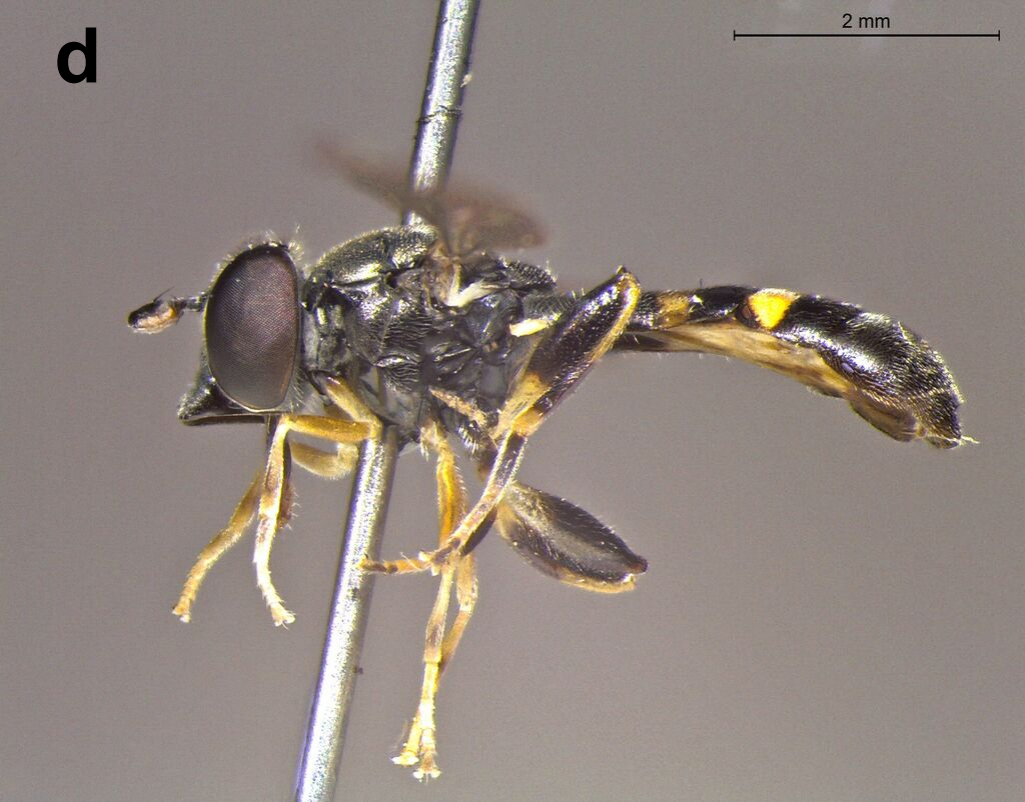
female in profile

**Figure 37a.**
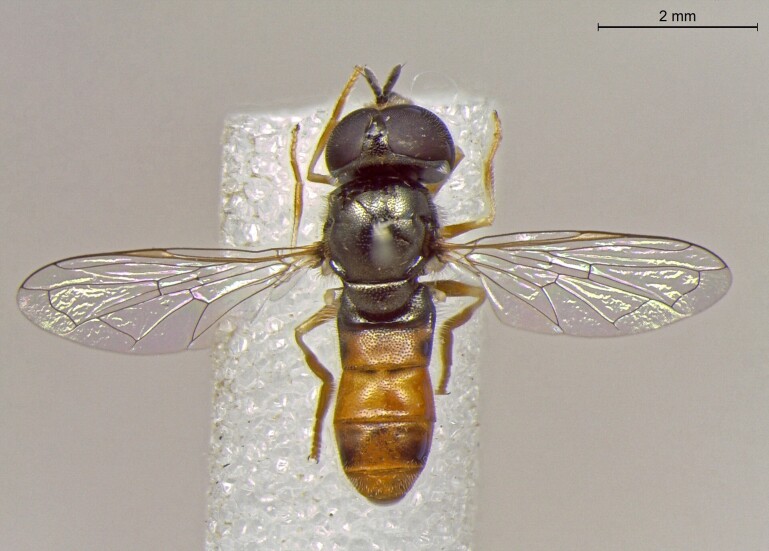
male in dorsal view

**Figure 37b.**
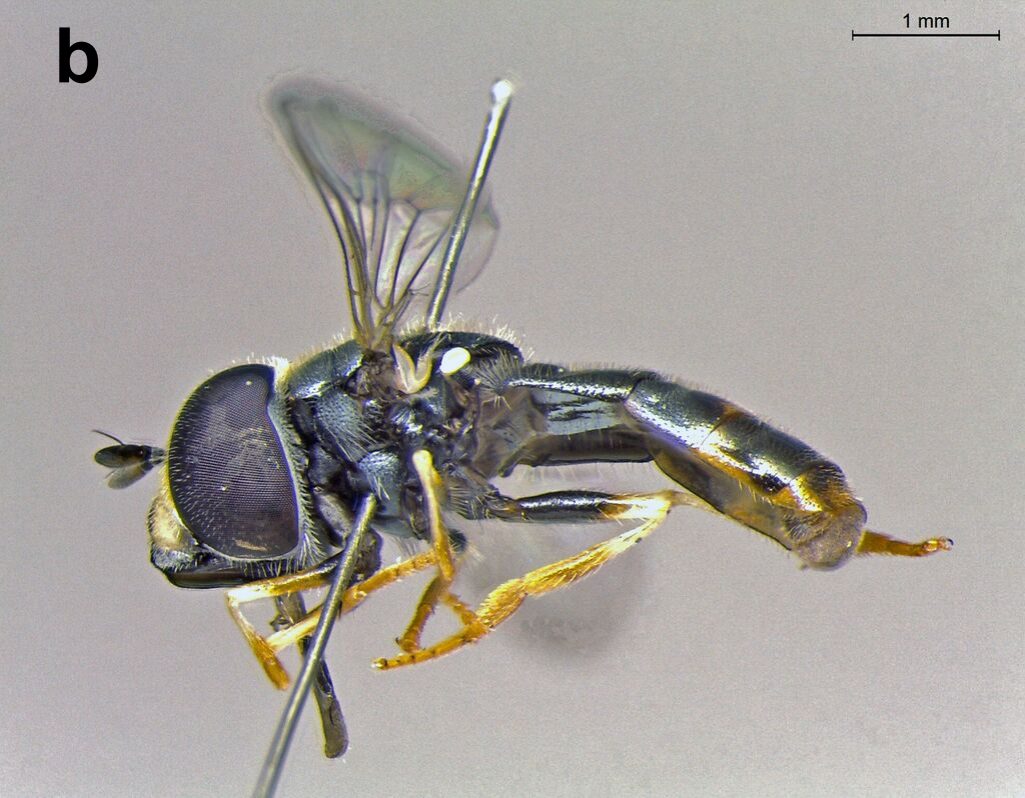
male in profile

**Figure 37c.**
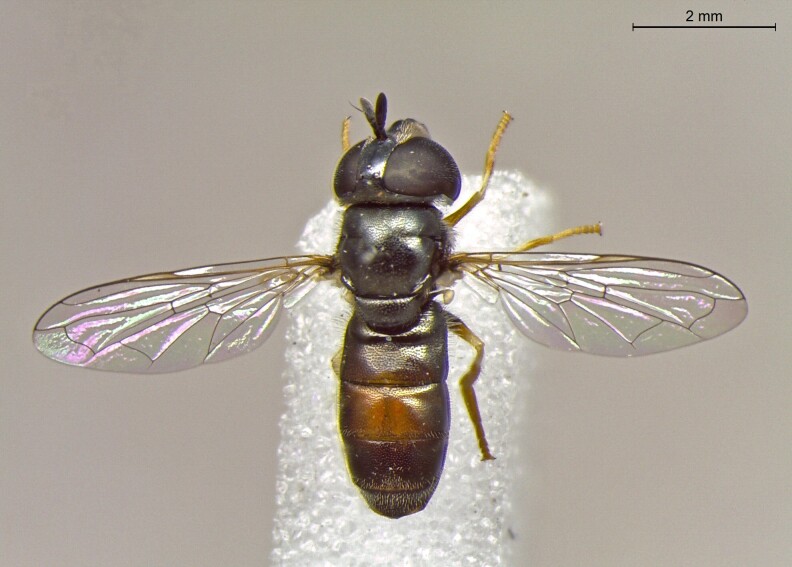
female in dorsal view

**Figure 37d.**
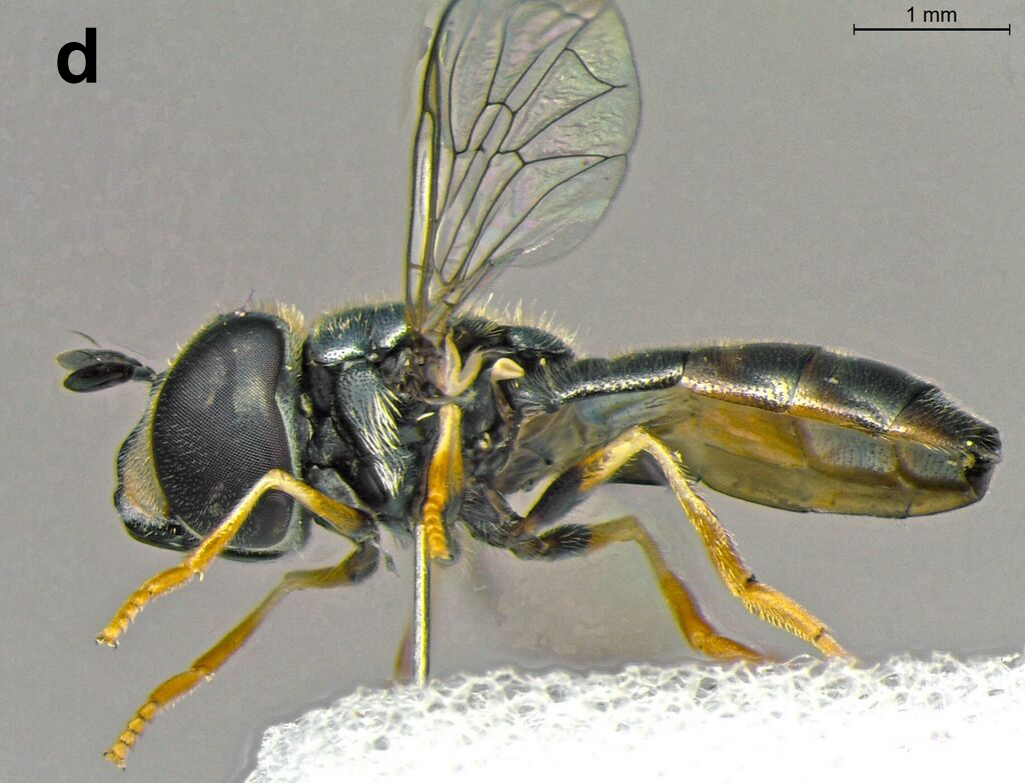
female in profile

**Figure 38a.**
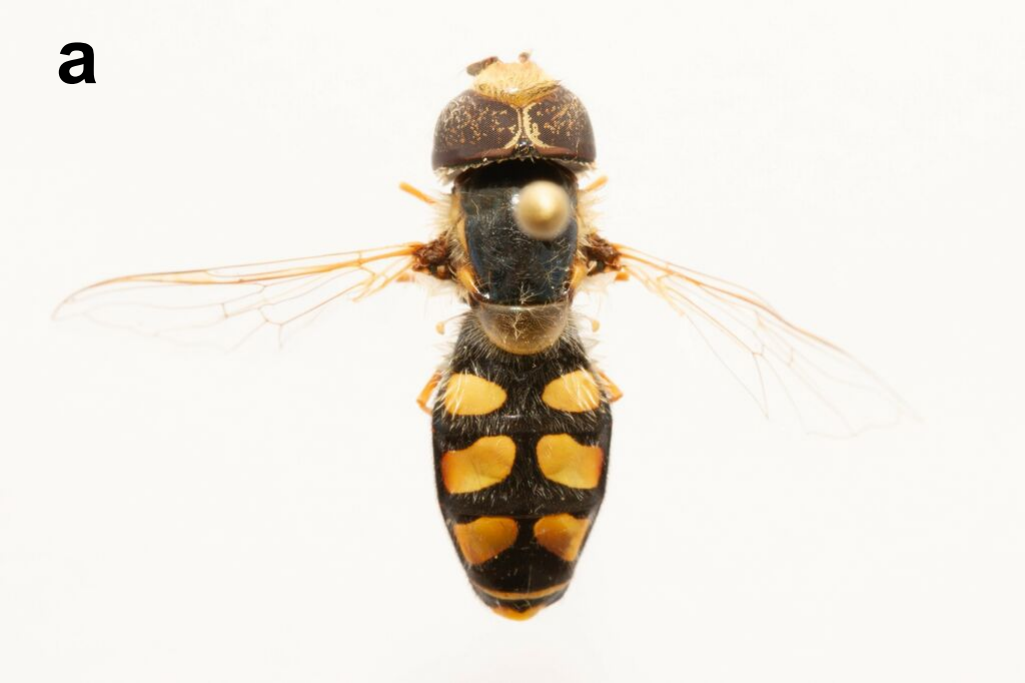
male in dorsal view

**Figure 38b.**
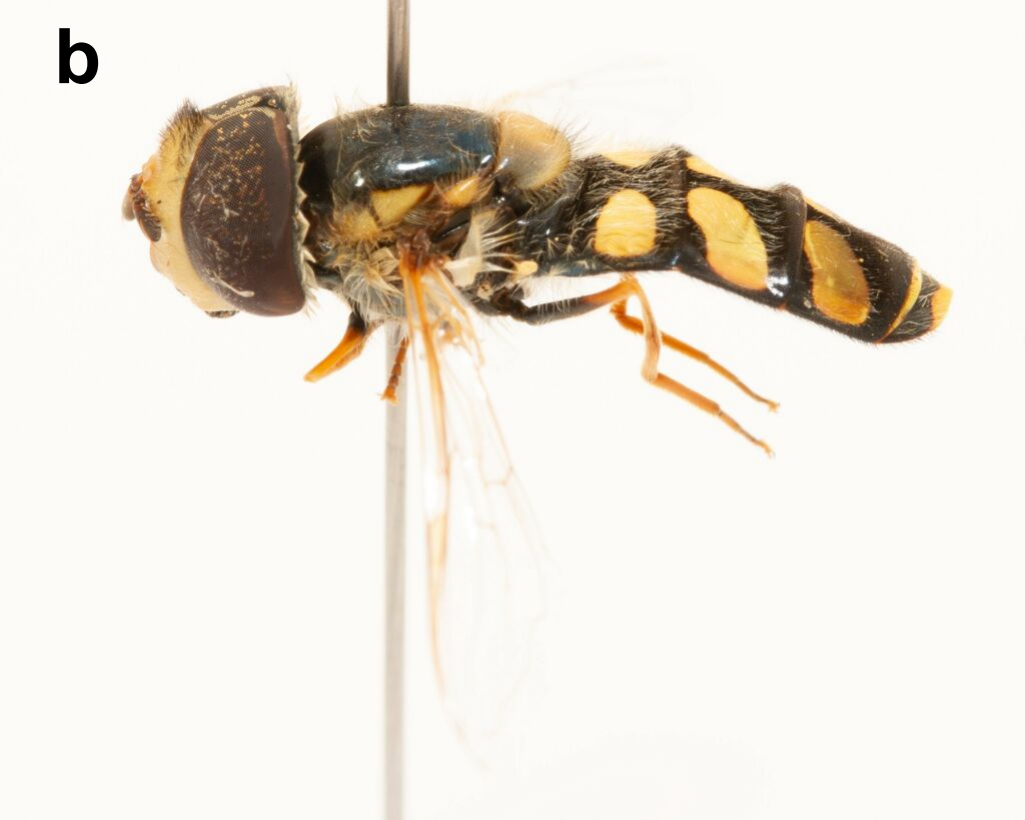
male in profile

**Figure 38c.**
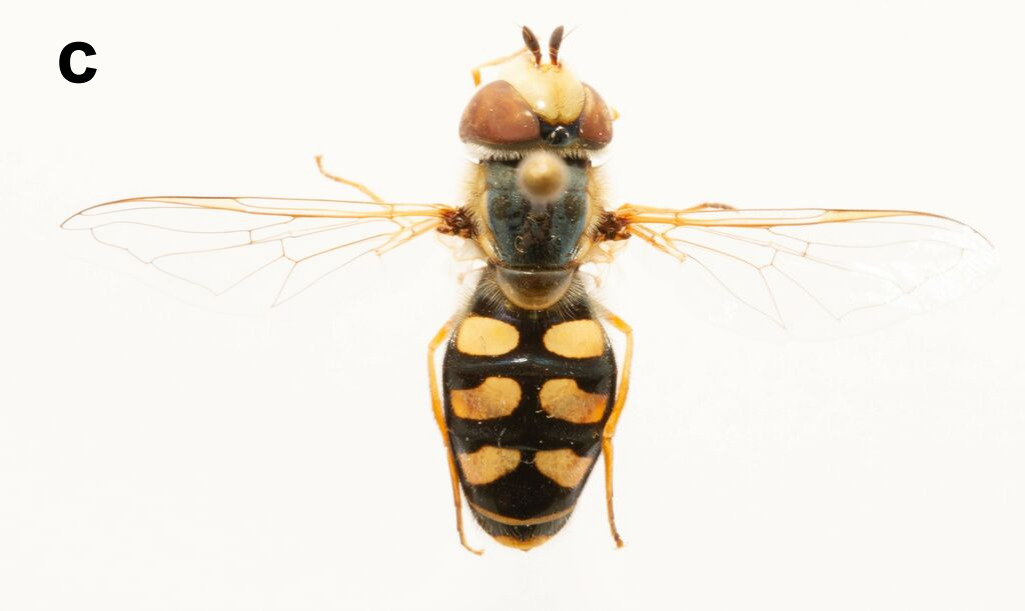
female in dorsal view

**Figure 38d.**
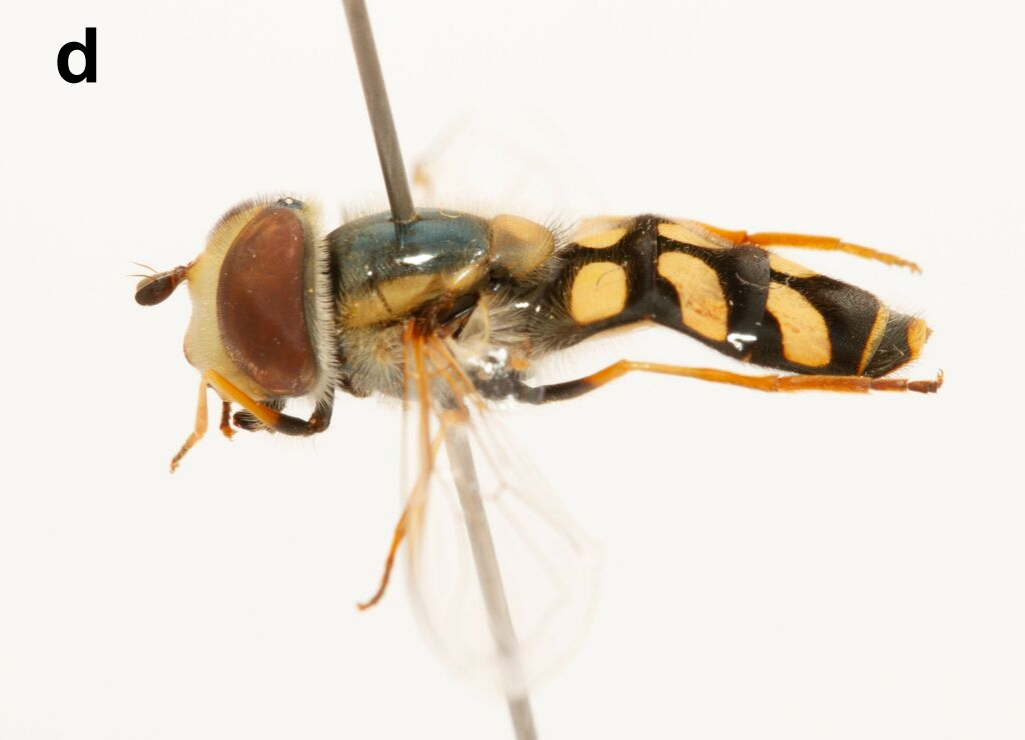
female in profile

**Figure 39a.**
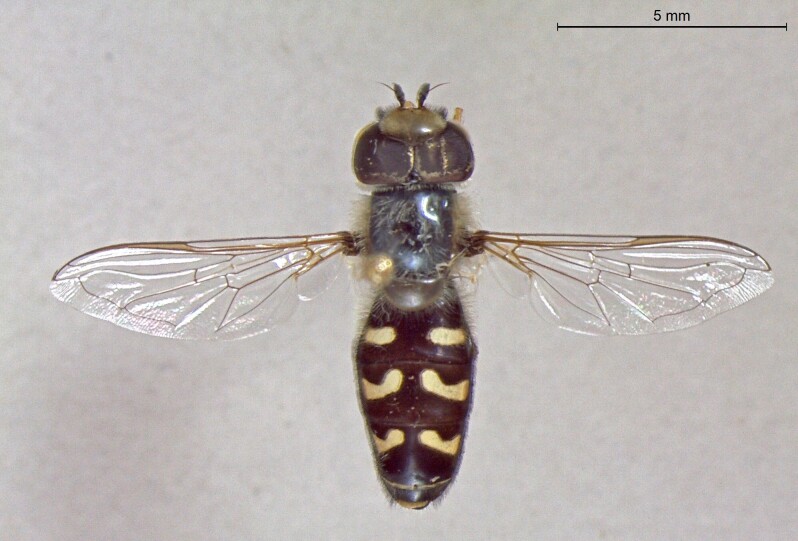
male in dorsal view

**Figure 39b.**
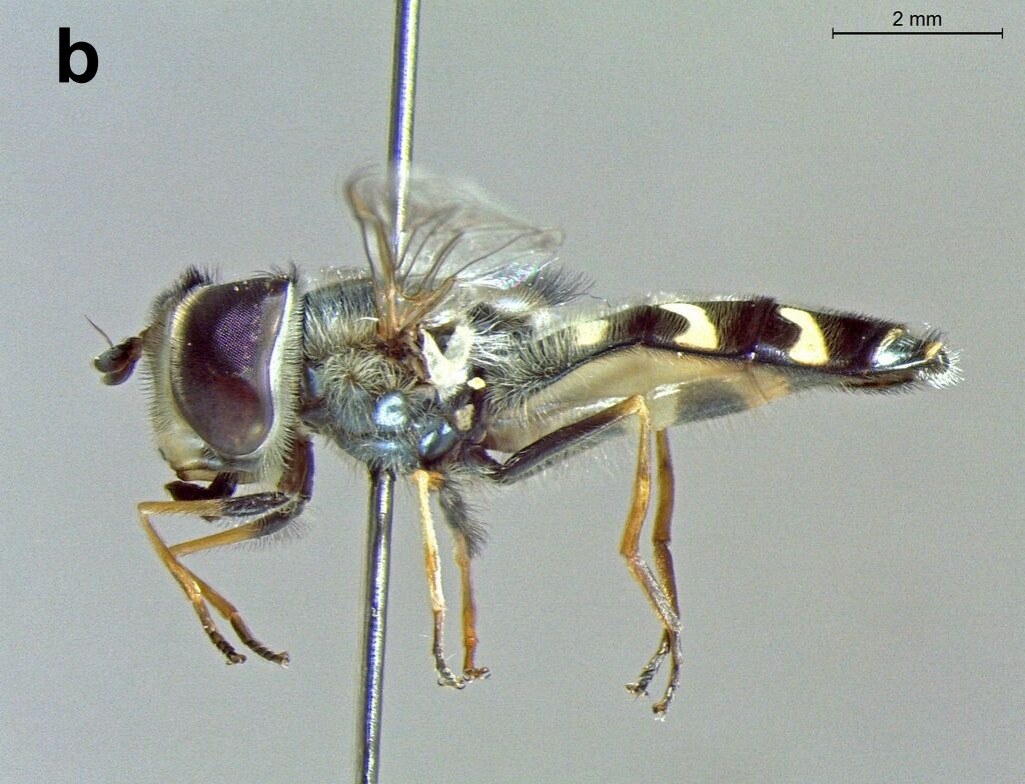
male in profile

**Figure 39c.**
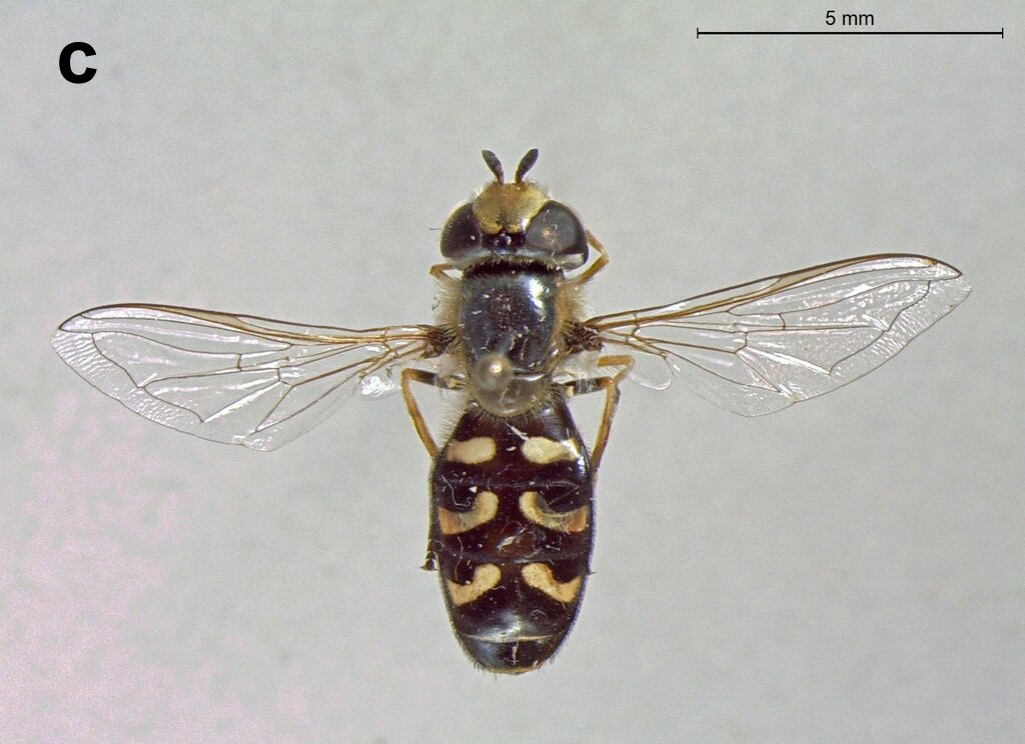
female in dorsal view

**Figure 39d.**
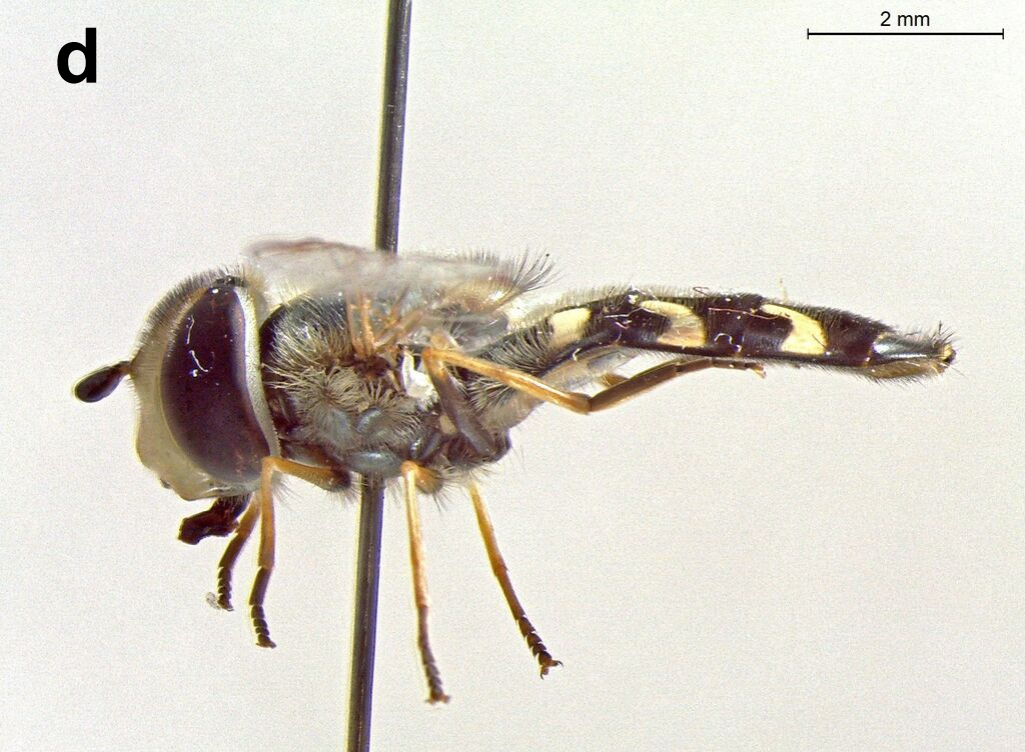
female in profile

**Figure 40a.**
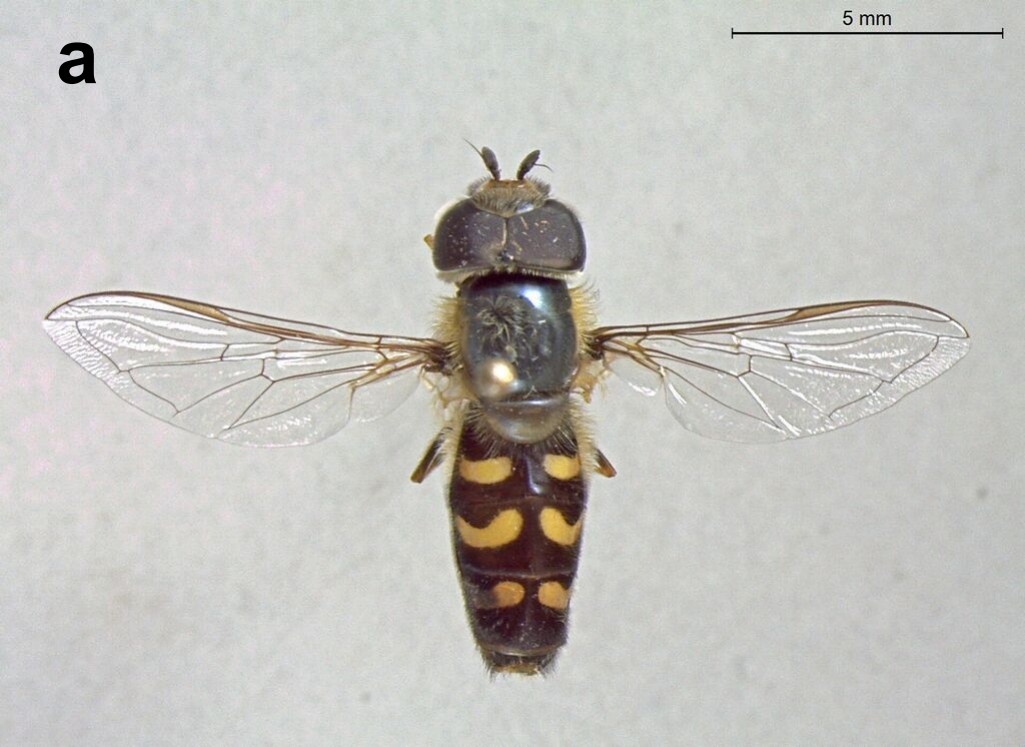
male in dorsal view

**Figure 40b.**
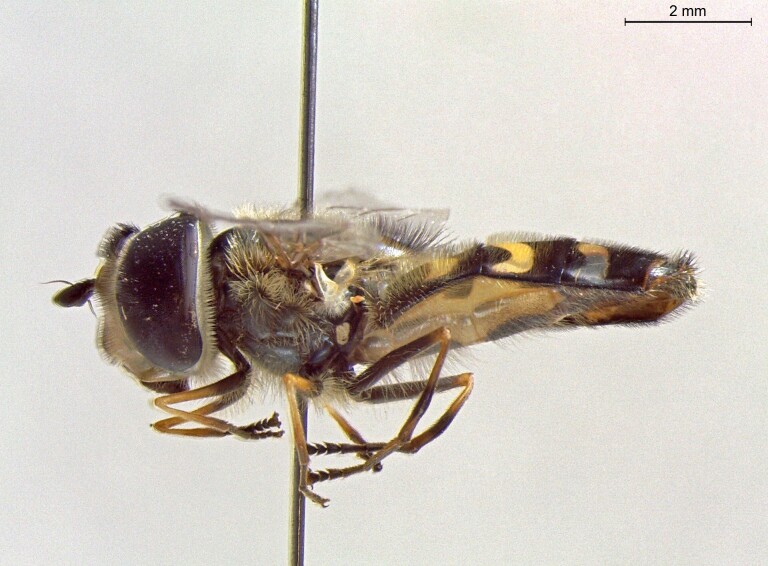
male in profile

**Figure 40c.**
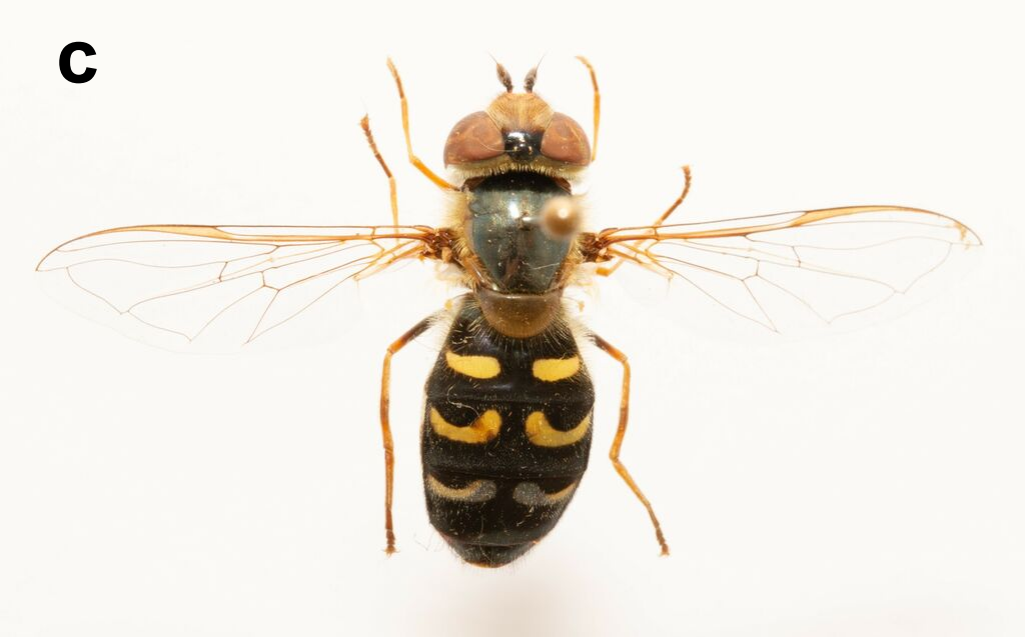
female in dorsal view

**Figure 40d.**
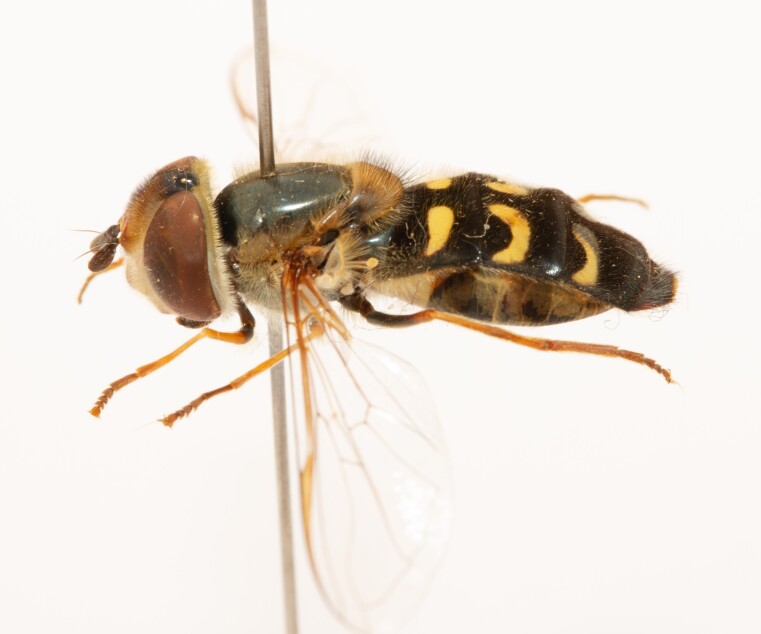
female in profile

**Figure 41a.**
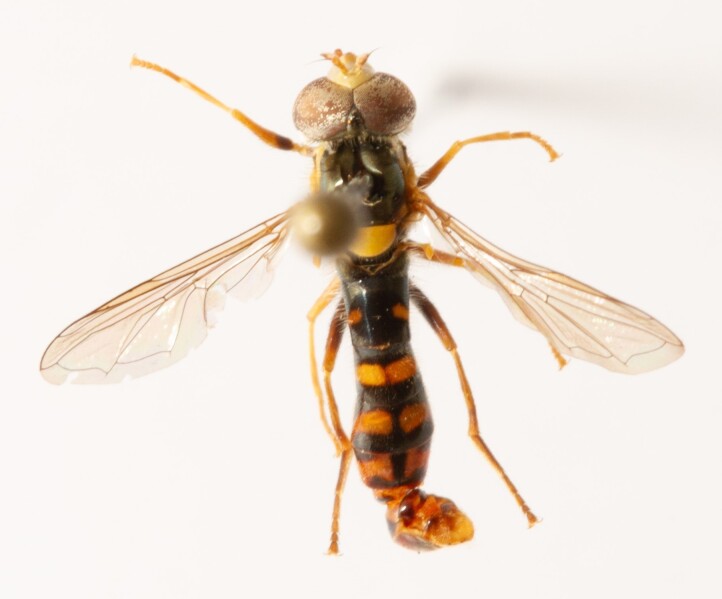
male in dorsal view

**Figure 41b.**
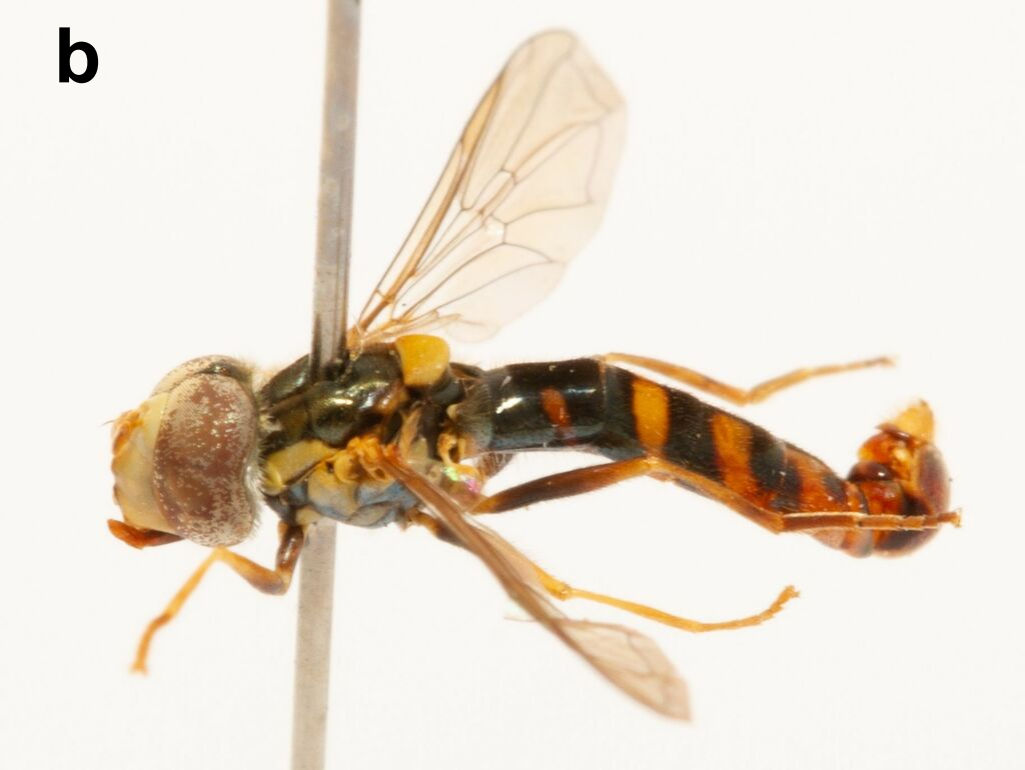
male in profile

**Figure 41c.**
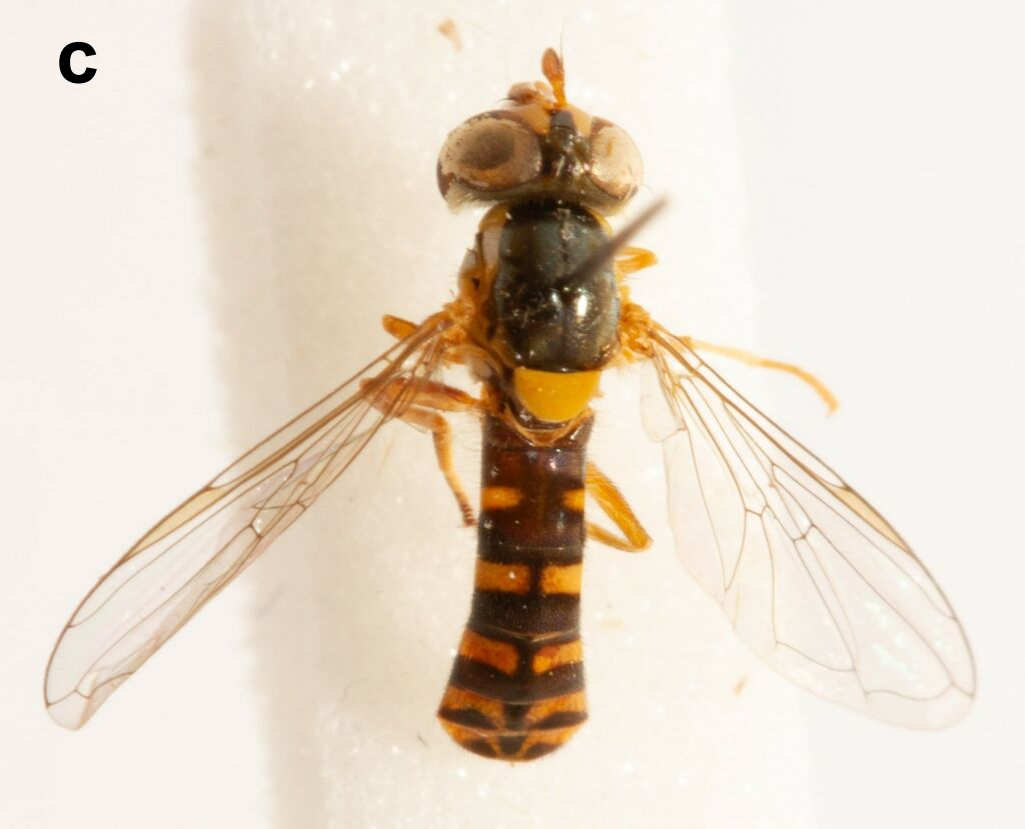
female in dorsal view

**Figure 41d.**
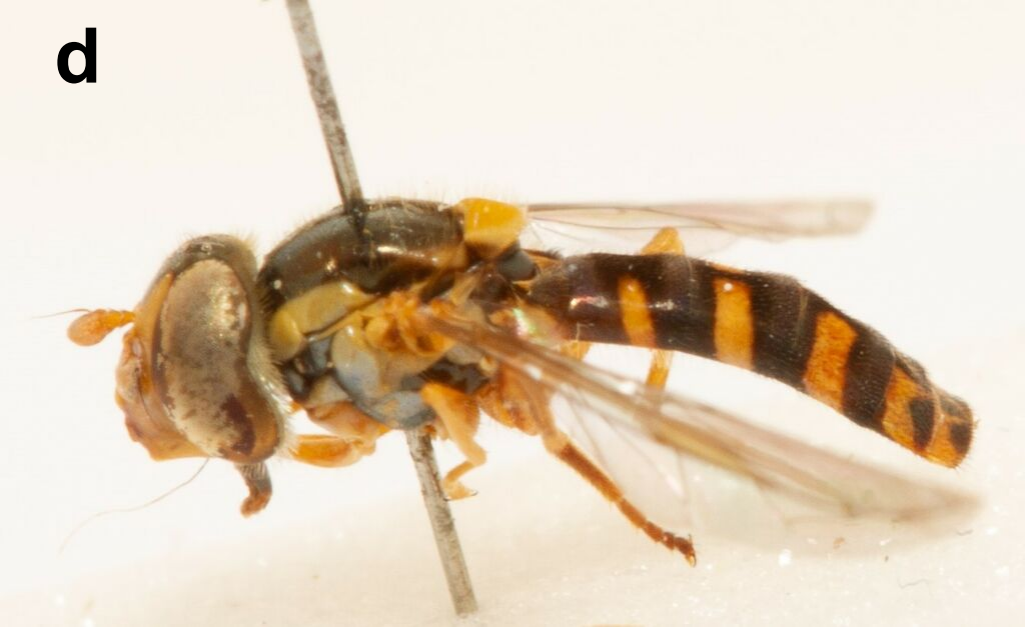
female in profile

**Figure 42a.**
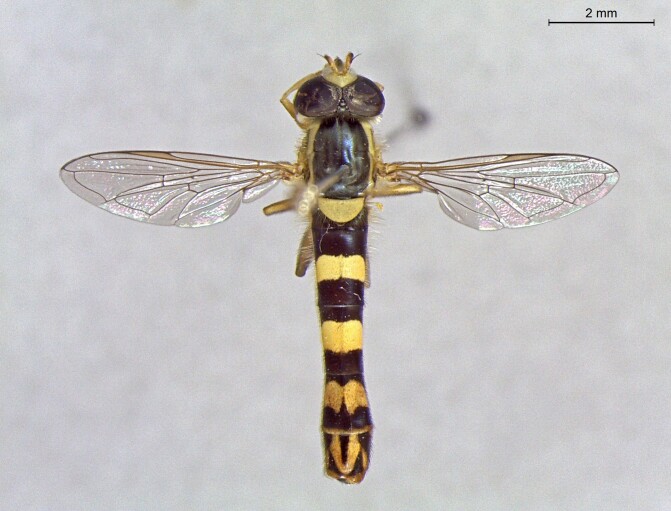
male in dorsal view

**Figure 42b.**
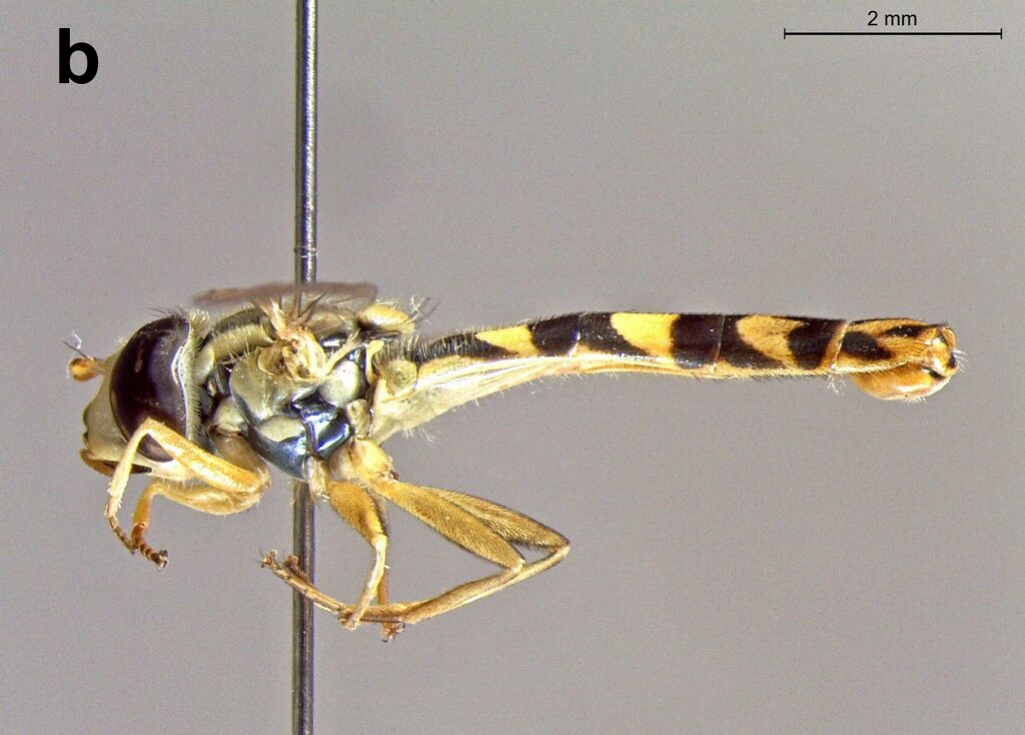
male in profile

**Figure 42c.**
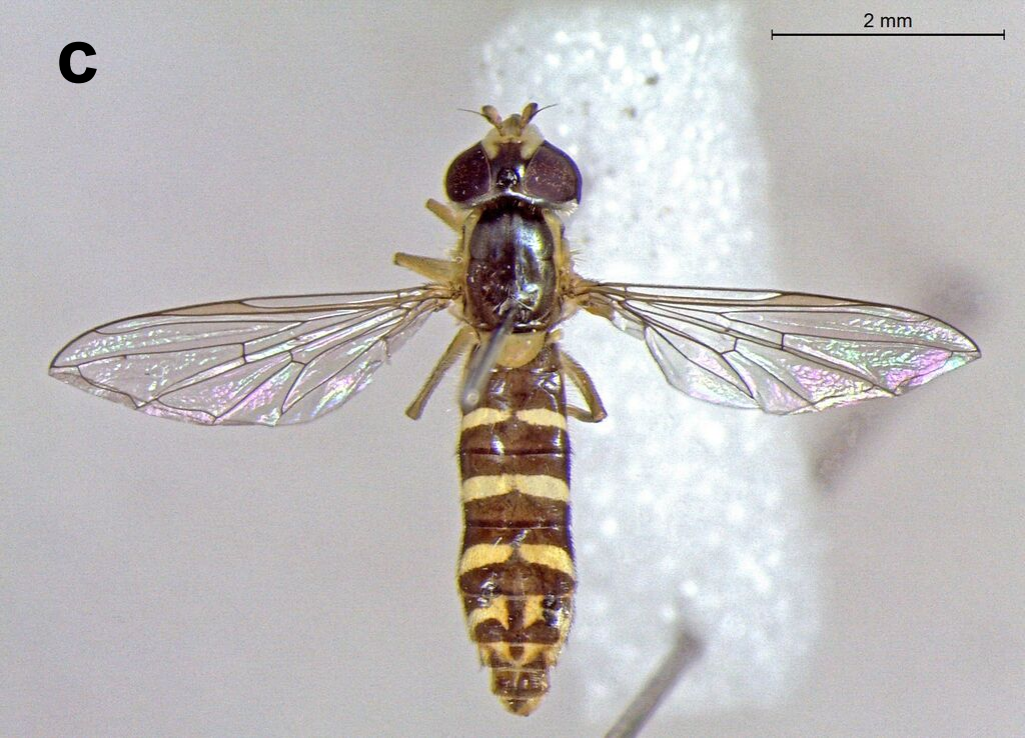
female in dorsal view

**Figure 42d.**
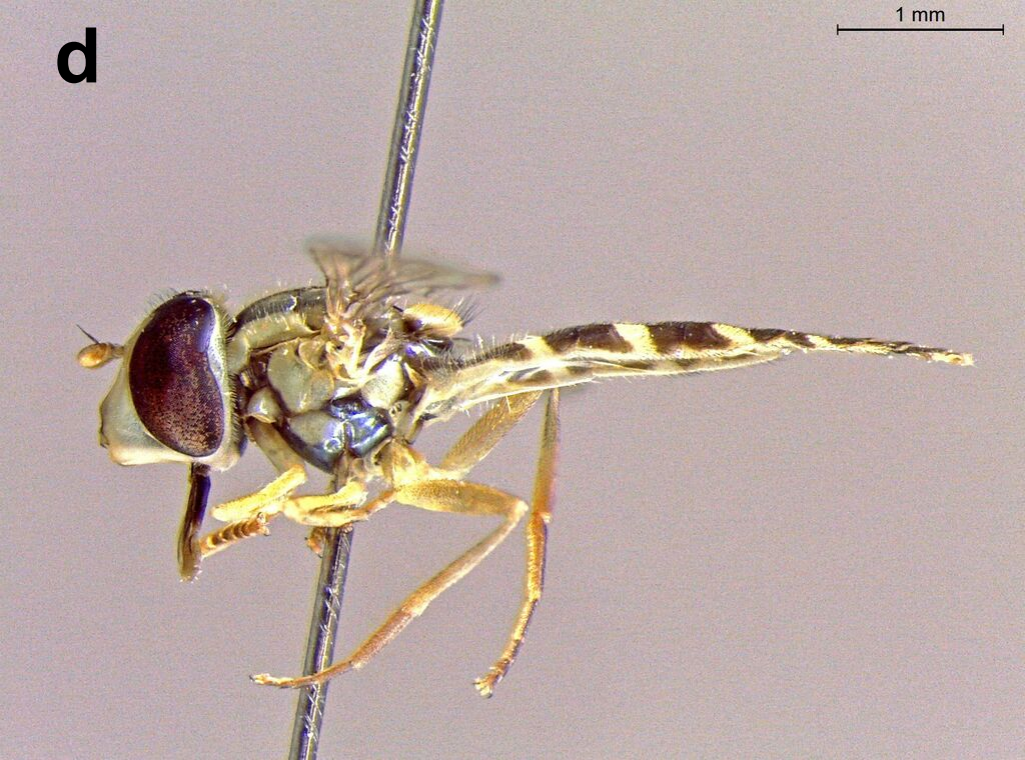
female in profile

**Figure 43a.**
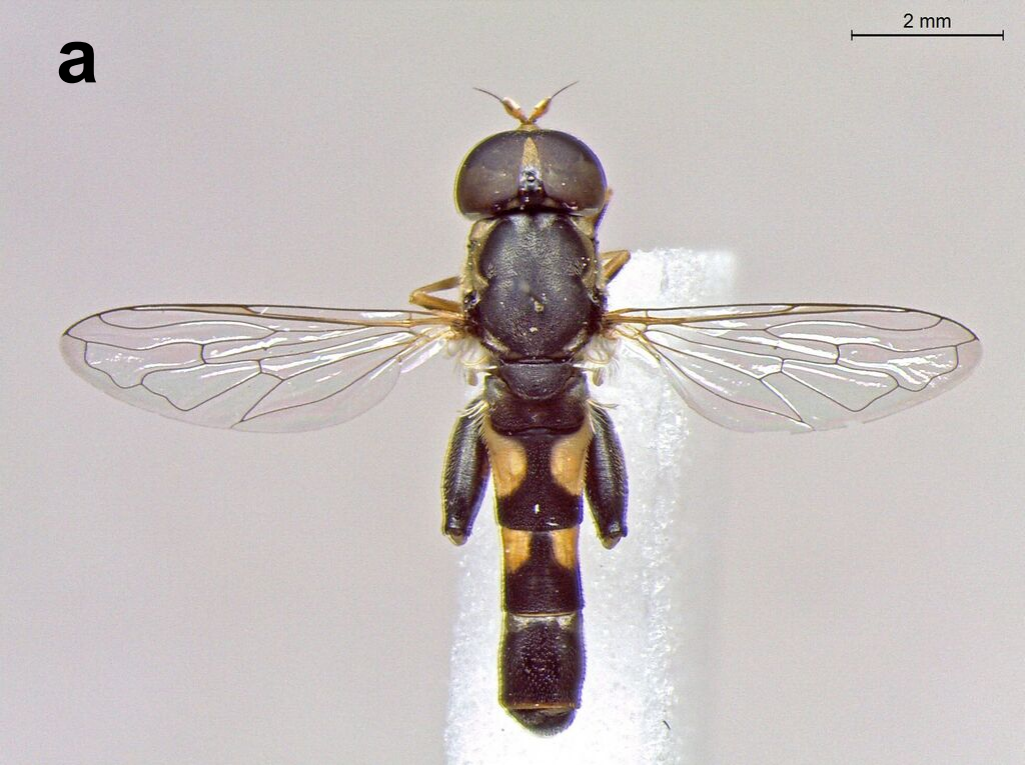
male in dorsal view

**Figure 43b.**
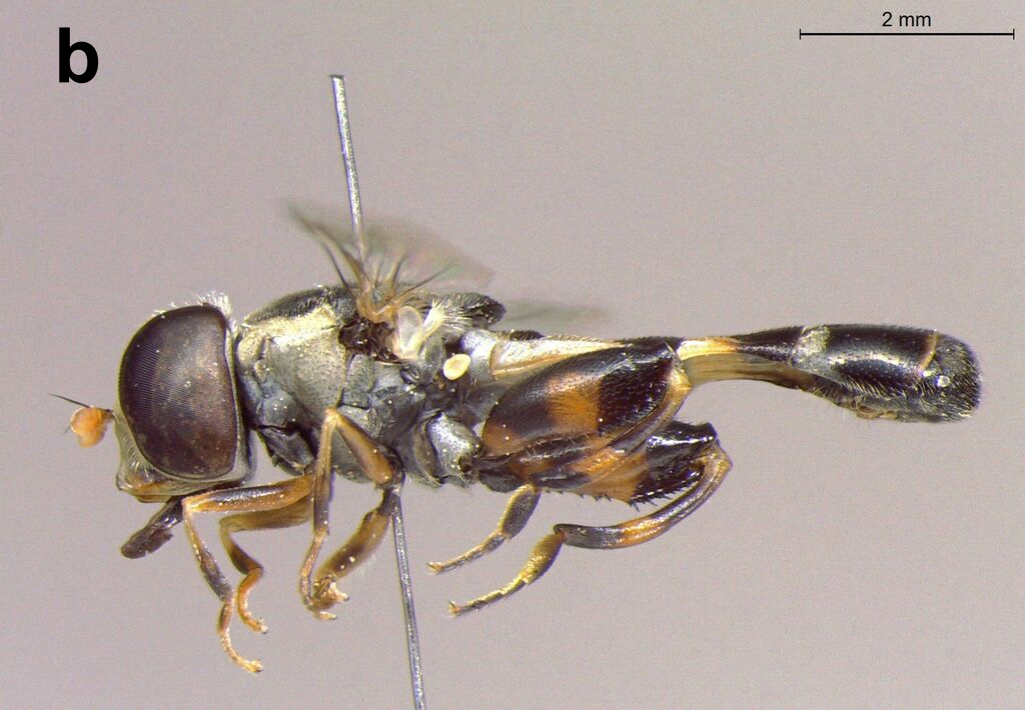
male in profile

**Figure 43c.**
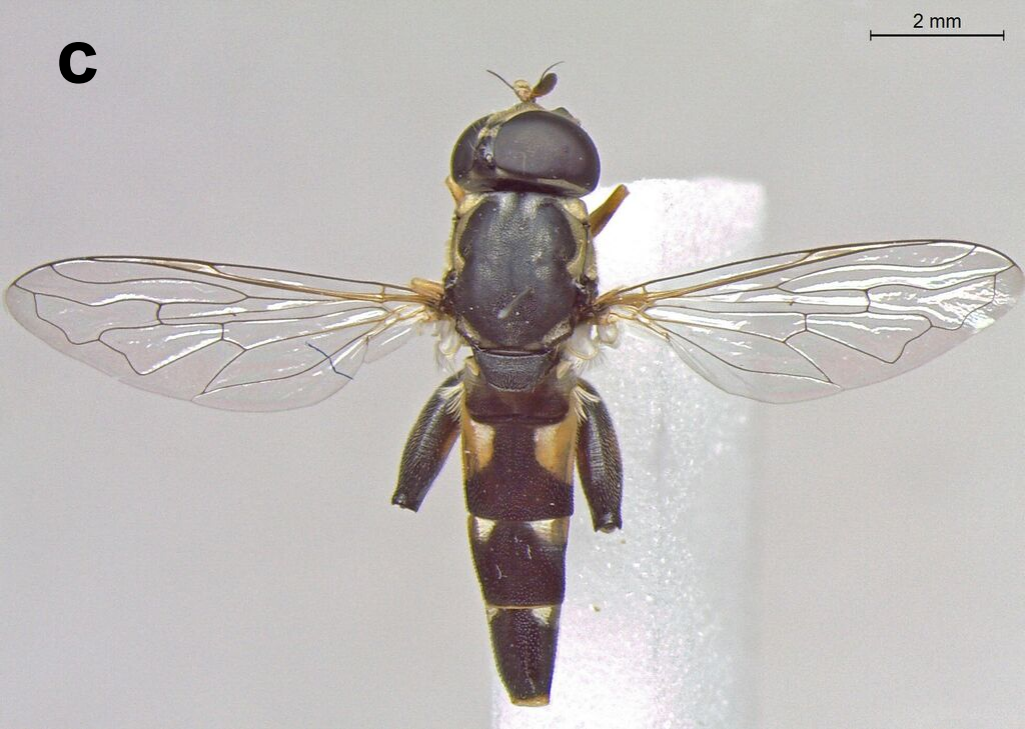
female in dorsal view

**Figure 43d.**
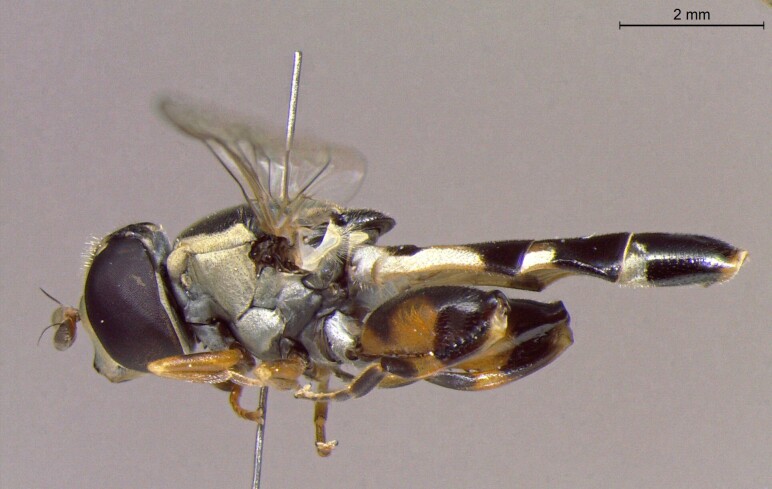
female in profile

**Figure 44a.**
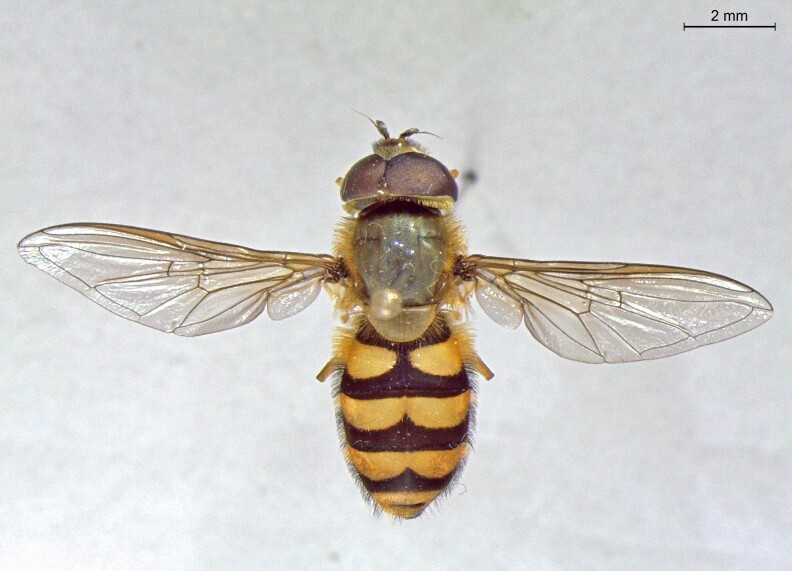
male in dorsal view

**Figure 44b.**
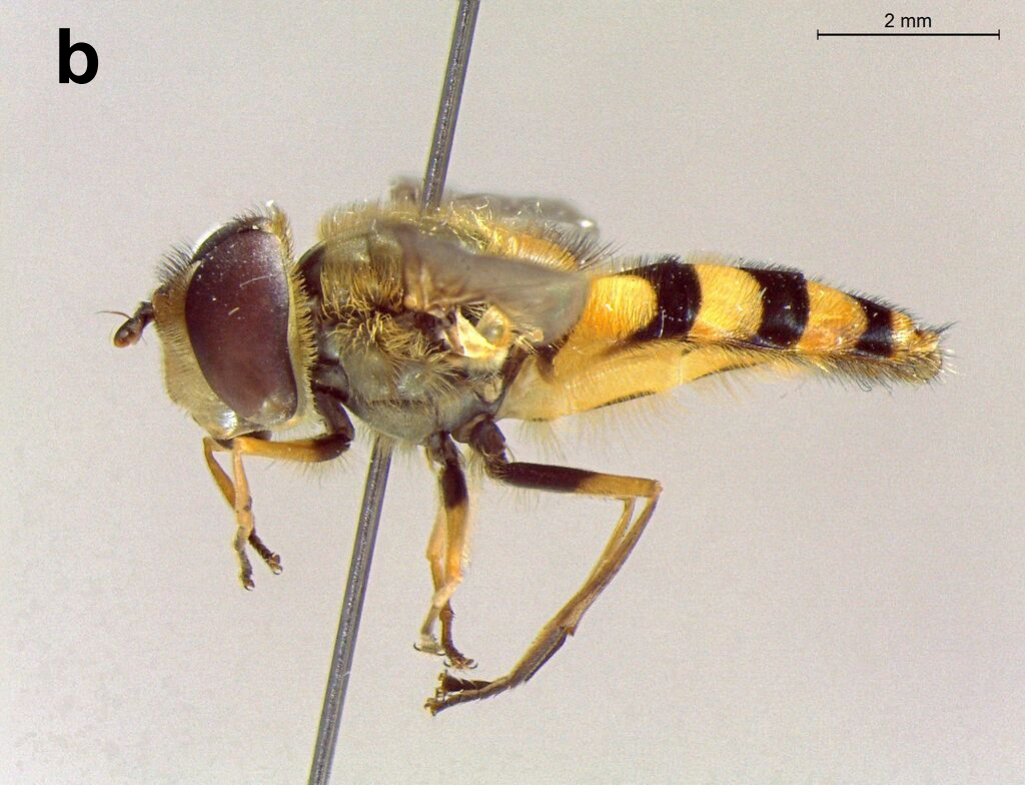
male in profile

**Figure 44c.**
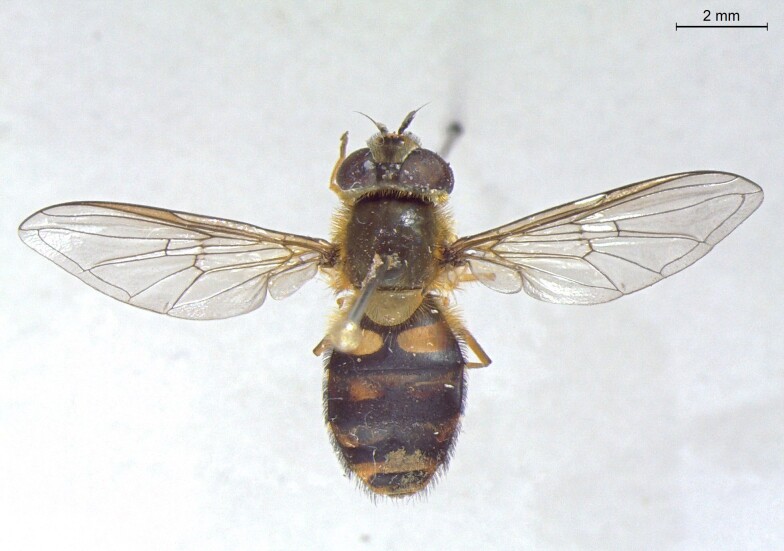
female in dorsal view

**Figure 44d.**
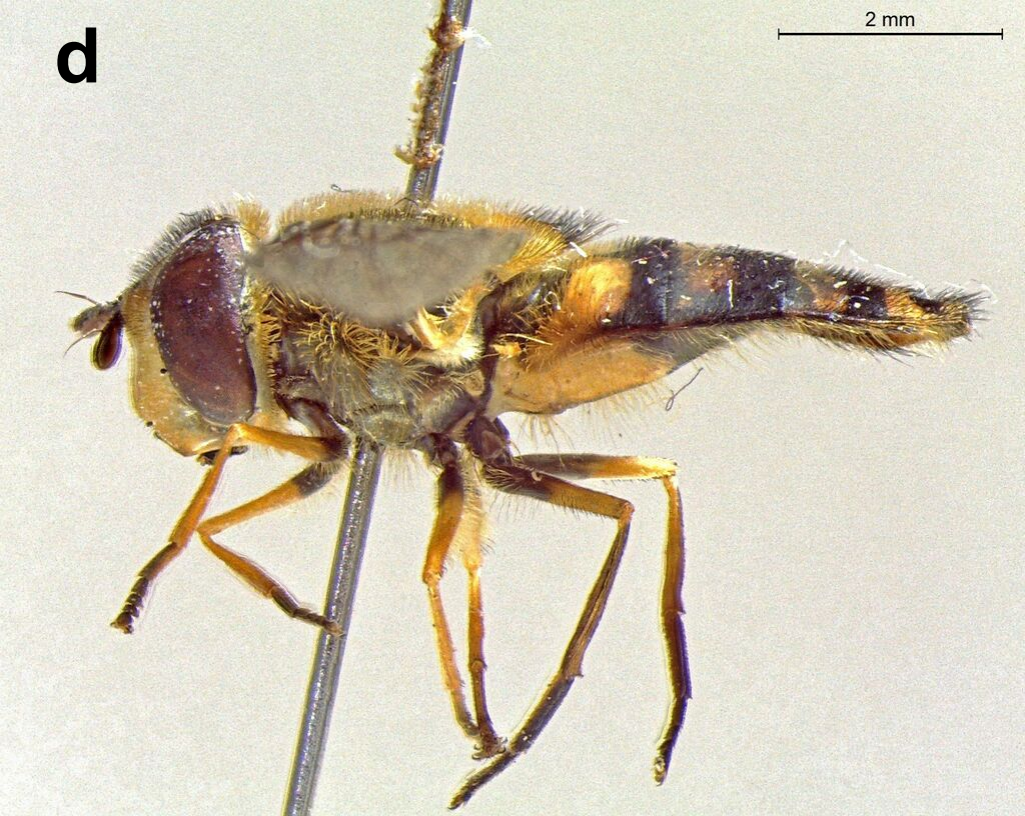
female in profile

**Figure 45a.**
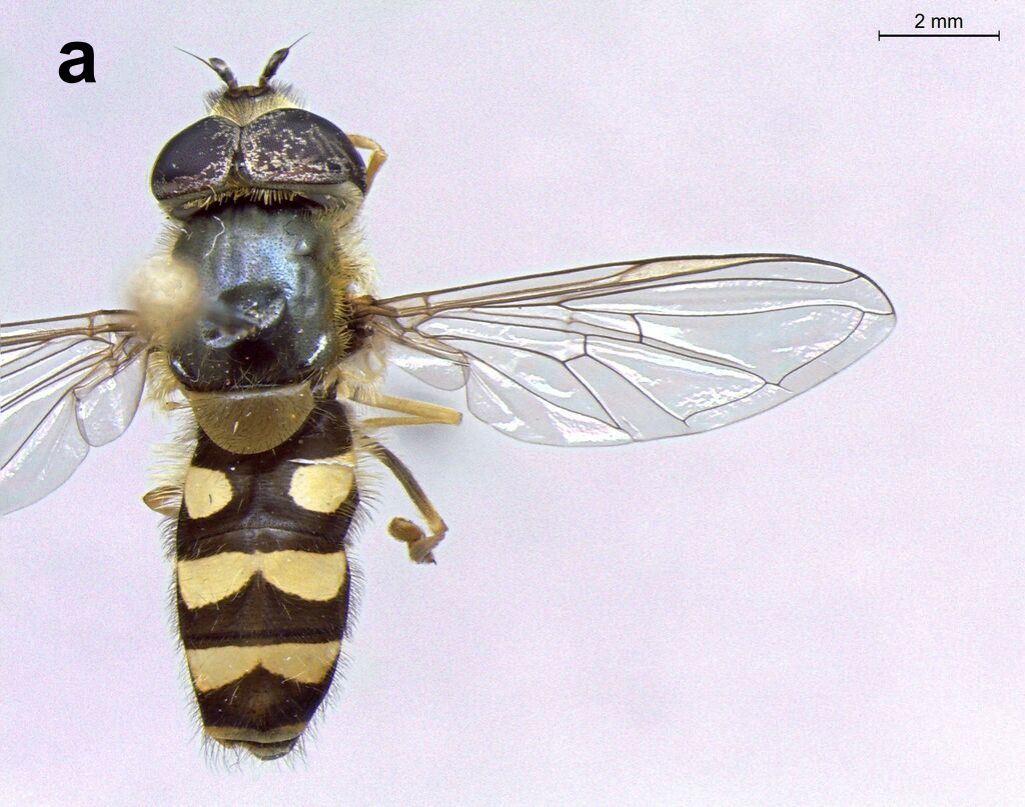
male in dorsal view

**Figure 45b.**
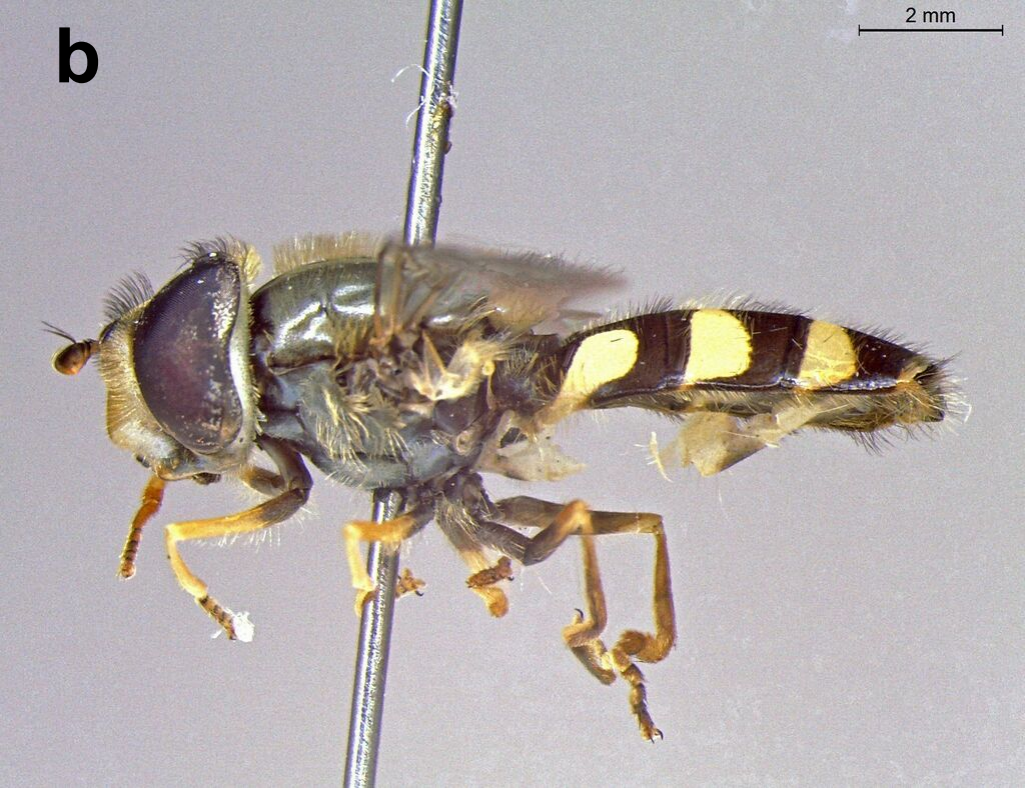
male in profile

**Figure 45c.**
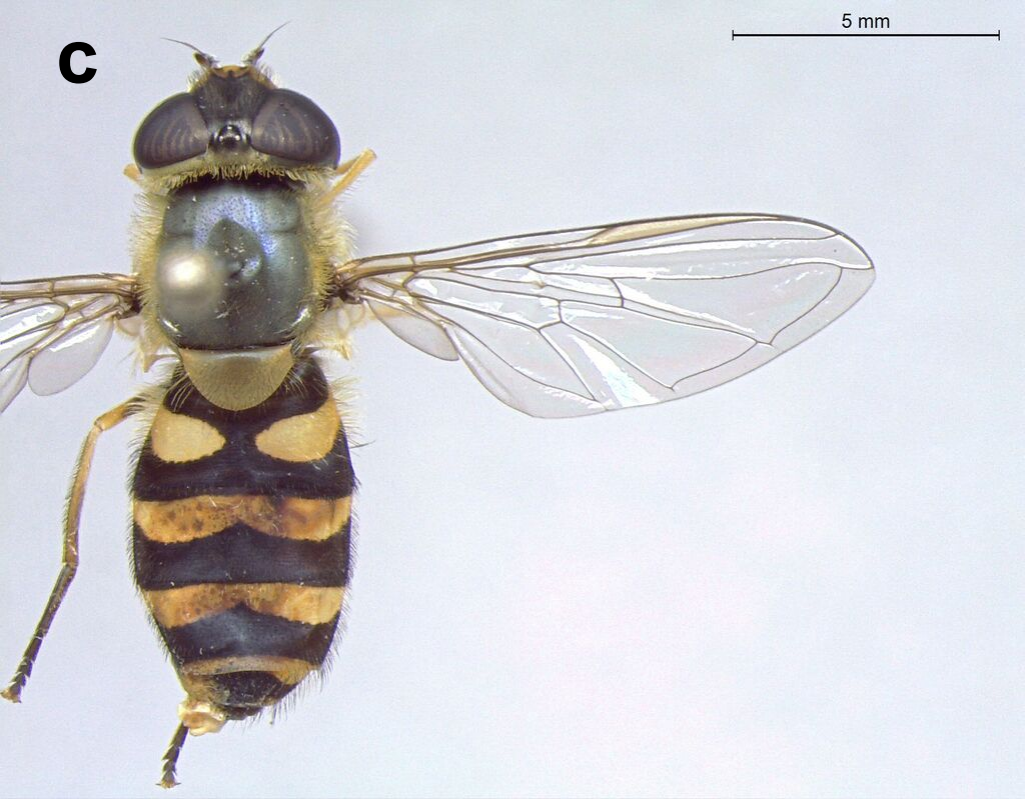
female in dorsal view

**Figure 45d.**
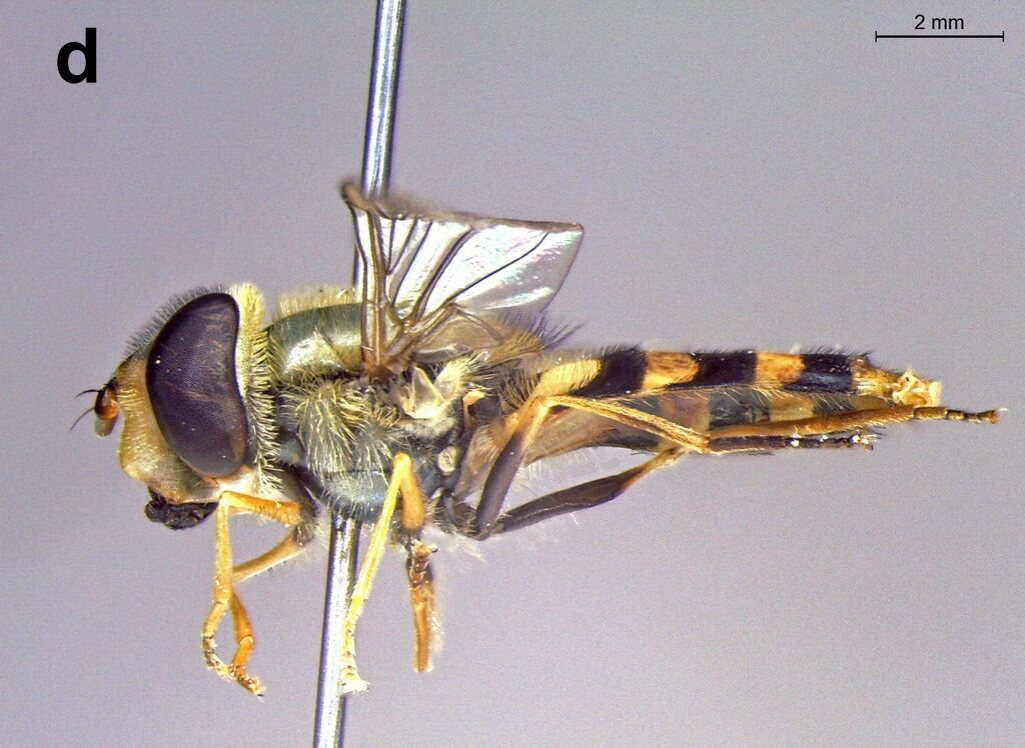
female in profile

**Figure 46a.**
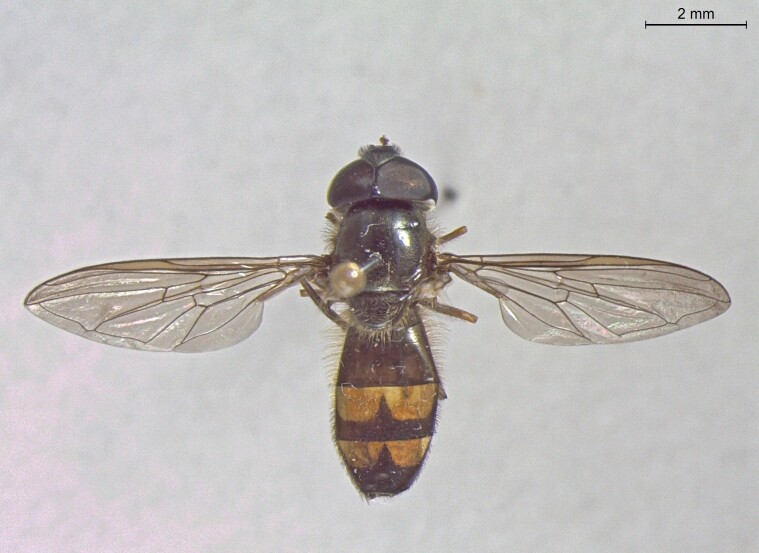
male in dorsal view

**Figure 46b.**
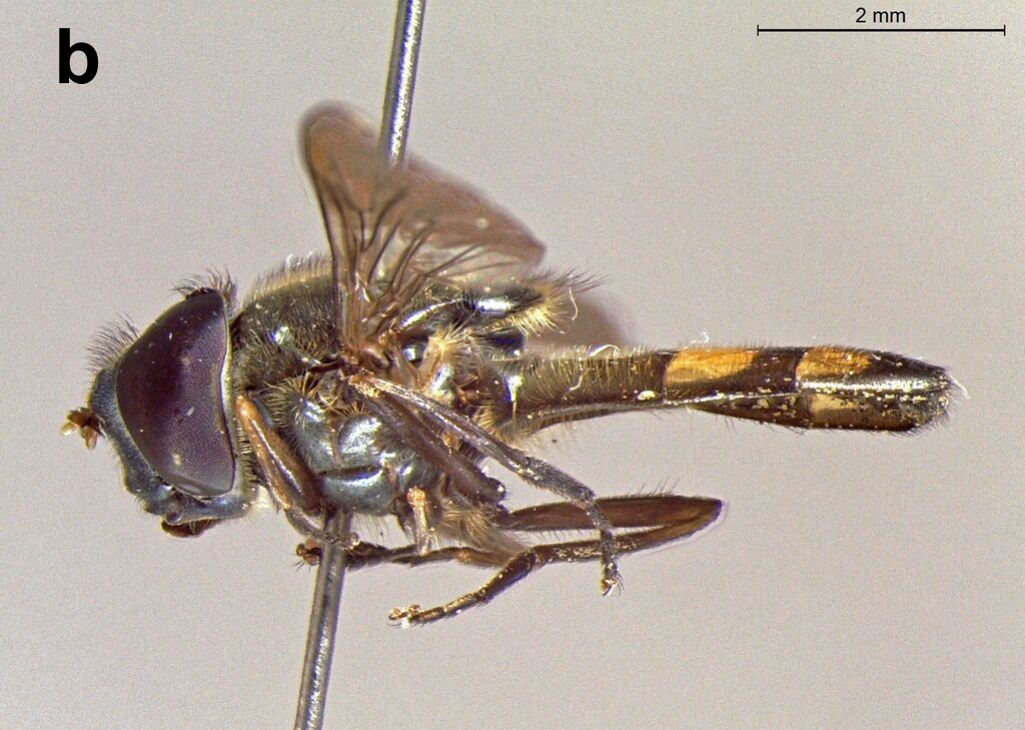
male in profile

**Figure 46c.**
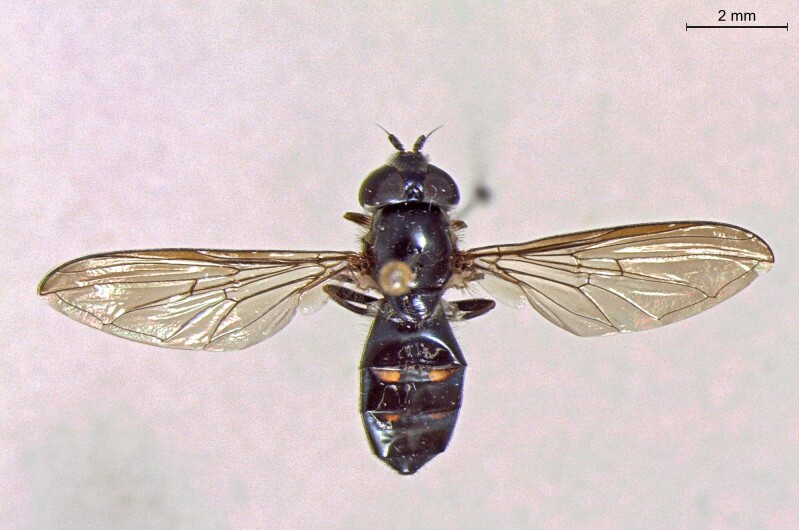
female in dorsal view

**Figure 46d.**
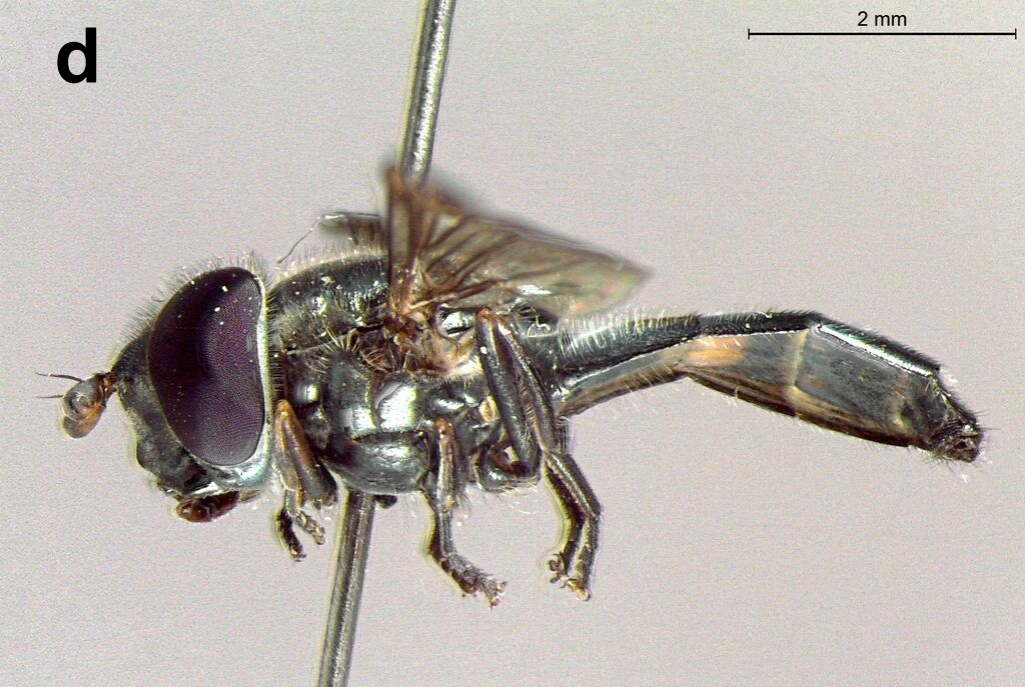
female in profile

**Figure 47a.**
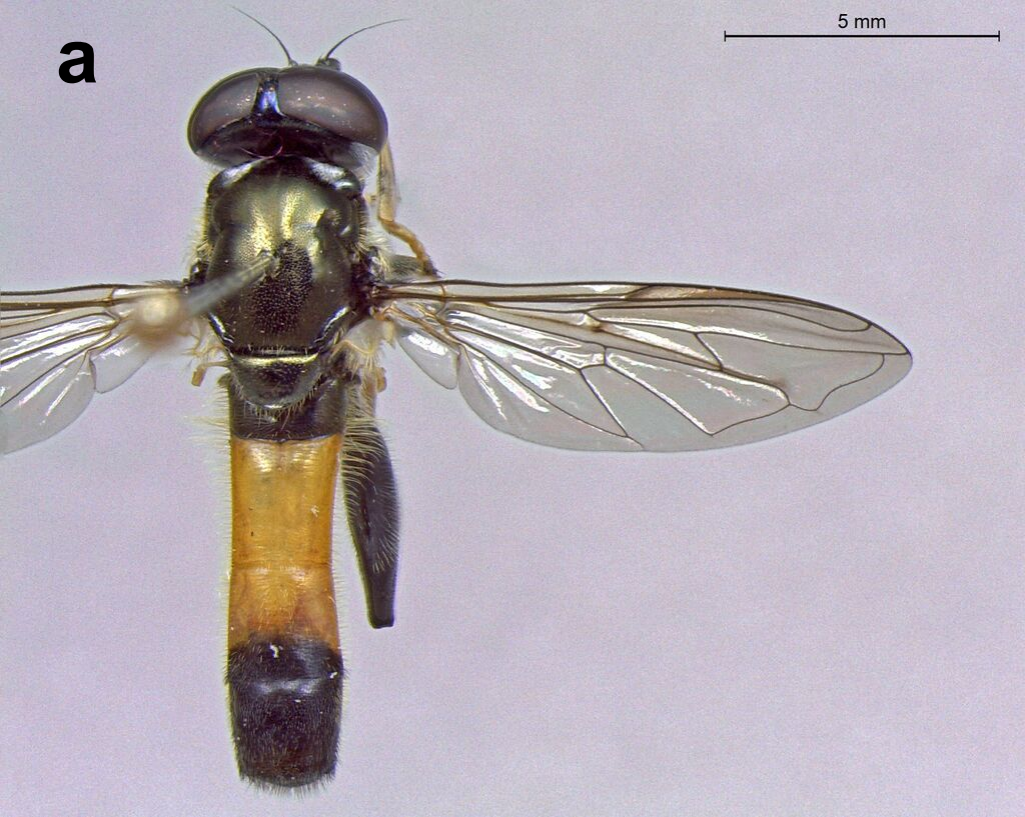
male in dorsal view

**Figure 47b.**
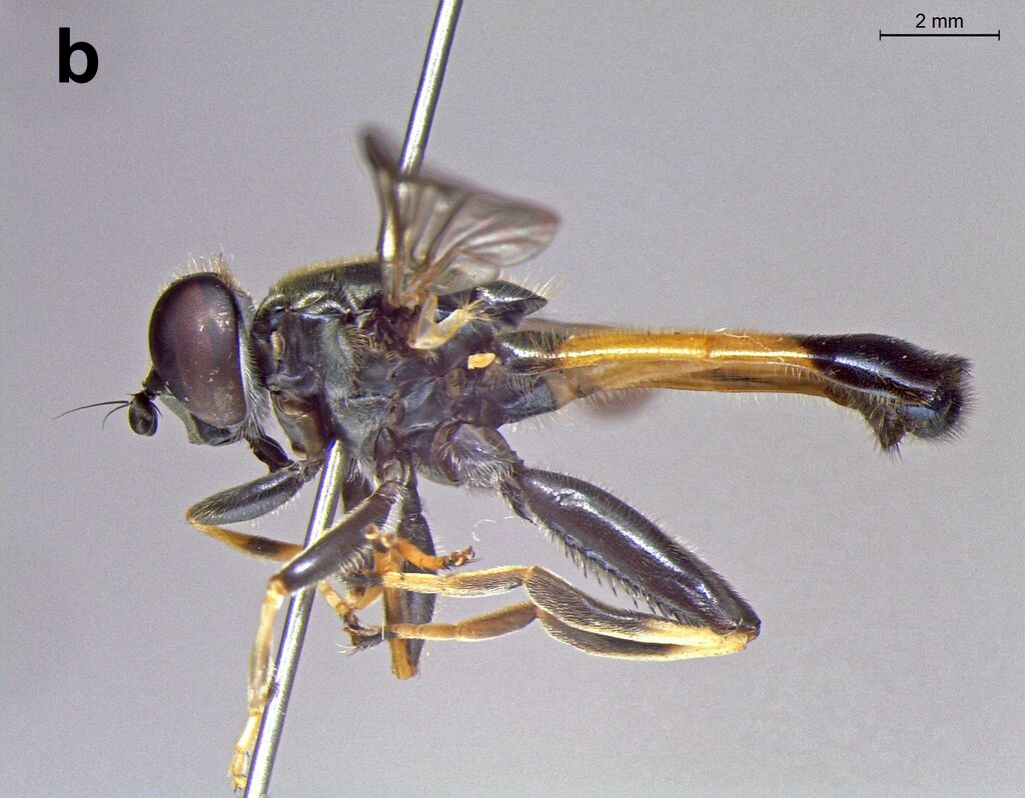
male in profile

**Figure 47c.**
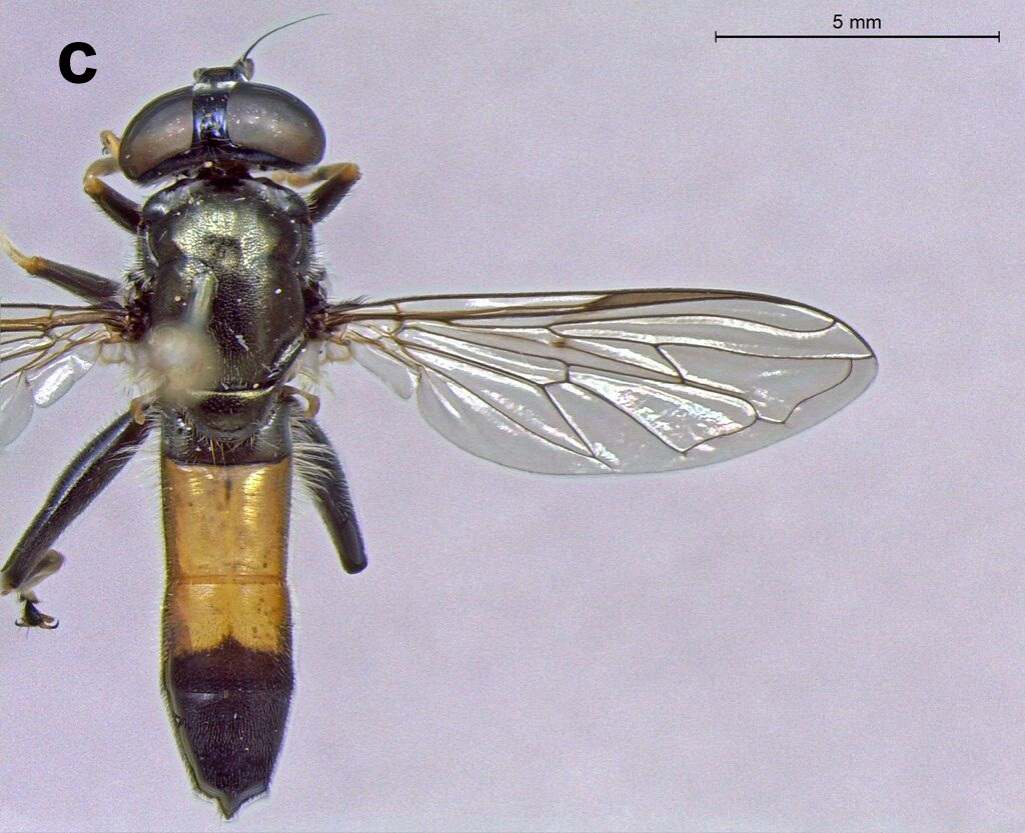
female in dorsal view

**Figure 47d.**
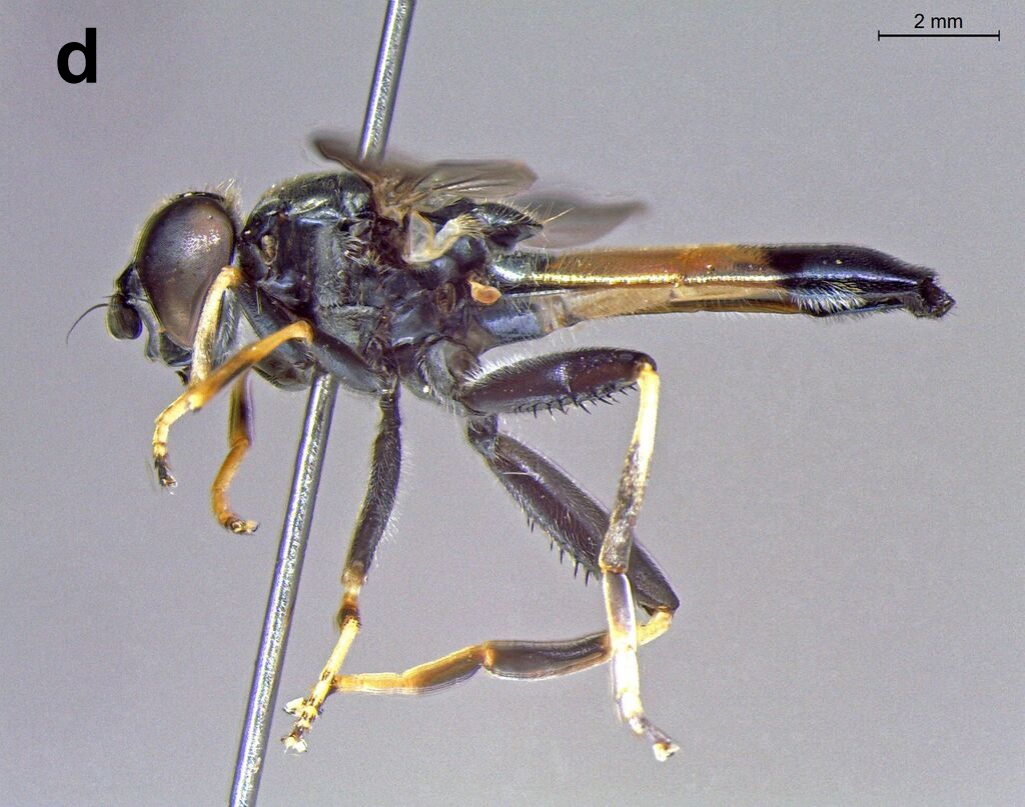
female in profile

**Table 1.** List of the Syrphidae species from Madeira, their distribution in the Archipelago (M – Madeira Island, PS – Porto Santo Island and surrounding islets, D – Desertas Islands) and representative photos of adult males and females (in dorsal and lateral views).

**Species**	**M**	**PS**	**D**	**Photos**
*Episyrphusbalteatus* (De Geer, 1776)	●	●	●	Fig. 22
*Eristalinusaeneus* (Scopoli, 1763)	●	●		Fig. 23
*Eristalinustaeniops* (Wiedemann, 1818)	●			Fig. 24
*Eristalistenax* (Linnaeus, 1758)	●	●	●	Fig. 25
*Eumerushispidus* Smit et al., 2004	●	●		Fig. 26
*Eupeodescorollae* (Fabricius, 1794)	●	●	●	Fig. 27
*Eupeodesluniger* (Meigen, 1822)	●	●	●	Fig. 28
*Eupeodesnuba* (Wiedemann, 1830)	●			Fig. 29
*Ischiodonaegyptius* (Wiedemann, 1830)	●	●	●	Fig. 30
*Melanostomamellinum* (Linnaeus, 1758)	●	●		Fig. 31
*Melanostomawollastoni* Wakeham-Dawson et al., 2004	●			Fig. 32
*Meliscaevaauricollis* (Meigen, 1822)	●			Fig. 33
*Milesiacrabroniformis* (Fabricius, 1775)	●			Fig. 34
*Myathropausta* (Wollaston, 1858)	●			Fig. 35
*Neoasciapodagrica* (Fabricius, 1775)	●			Fig. 36
*Paragusmundus* Wollaston, 1858	●	●		Fig. 37
*Scaevaalbomaculata* (Macquart, 1842)		●	●	Fig. 38
*Scaevapyrastri* (Linnaeus, 1758)	●	●	●	Fig. 39
*Scaevaselenitica* (Meigen, 1822)	●			Fig. 40
*Sphaerophoriarueppellii* (Wiedemann, 1830)	●	●		Fig. 41
*Sphaerophoriascripta* (Linnaeus, 1758)	●	●		Fig. 42
*Syrittapipiens* (Linnaeus, 1758)	●	●		Fig. 43
*Syrphustorvus* Osten-Sacken, 1875	●			Fig. 44
*Syrphusvitripennis* Meigen, 1822	●			Fig. 45
*Xanthandrusbabyssa* (Walker, 1849)	●			Fig. 46
*Xylotasegnis* (Linnaeus, 1758)	●			Fig. 47
